# Public omics data reuse in gastric cancer: from discovery abundance to translational evidence

**DOI:** 10.3389/fonc.2026.1974260

**Published:** 2026-10-26

**Authors:** Shuqiong Su, Cong Huang, Bo Yang, Yueli Huang, Panyang Yang, Aiqun Liu

**Affiliations:** 1 Department of Gastroenterology, Guangxi Medical University Cancer Hospital, Nanning, Guangxi, China; 2 Department of Chemotherapy, Guangxi Medical University First Affiliated Hospital, Nanning, Guangxi, China; 3 Department of Oncology, The People’s Hospital of Yichun City, Yichun, Jiangxi, China; 4 Department of Radiation Oncology, Sun Yat-sen University Cancer Center, Guangzhou, Guangdong, China

**Keywords:** bioinformatics, databases, factual, prognosis, reproducibility of results, stomach neoplasms

## Abstract

Public-omics data are now widely reused in gastric cancer bioinformatics. Resources such as The Cancer Genome Atlas stomach adenocarcinoma cohort (TCGA-STAD), the Gene Expression Omnibus (GEO), cBioPortal, proteogenomic cohorts, single-cell atlases, and spatial datasets make secondary analysis fast and inexpensive. They are used to study tumor biology, biomarkers, prognosis, therapy-response hypotheses, and tumor-microenvironment states. A major limitation in this literature is not data access. It is evidentiary dilution. Many studies reuse similar cohorts and analytical pipelines. They then produce additional gene lists or risk signatures without a corresponding improvement in independent validation, interpretation, or clinical relevance. This methodological narrative review synthesizes public-omics secondary analysis in gastric cancer from methodological, translational, and reproducibility-oriented perspectives, supported by reproducible PubMed database scans and public metadata checks conducted through 2026-06-30; both are descriptive signals and are not formal quality appraisals. The review has three aims. The first aim is to classify reusable public data resources and the recurring analytical workflows built on them. The second aim is to use prognostic modeling as a stress test for leakage, overfitting, weak validation, and overinterpreted clinical value. The third aim is to propose GC-PROVE, a five-domain appraisal framework covering problem anchoring, resource provenance, omics workflow transparency, validation hierarchy, and evidence restraint. Public-omics research is most useful when it produces auditable evidence, external validation, biological triangulation, and a clear clinical context.

## Highlights

- Public-omics reuse in gastric cancer is productive but increasingly repetitive; higher-value studies should add biological, methodological, or translational insight beyond additional gene lists.
- Prognostic signatures are the field’s clearest stress test because they are easy to produce and vulnerable to leakage, overfitting, weak validation, and overclaiming.
- GC-PROVE turns broad methodological advice into a practical appraisal mnemonic covering problem anchoring, resource provenance, workflow transparency, validation hierarchy, and evidence restraint.
- Single-cell, spatial, and foundation-model approaches add most value when they localize, validate, benchmark, or test bulk-derived signals.

## 1 Introduction

Gastric cancer remains a major contributor to global cancer morbidity and mortality, and its molecular heterogeneity complicates biomarker discovery, prognostic assessment, and treatment-response prediction (1–3). Public-omics resources have changed how investigators approach this problem. Instead of beginning every project by generating new sequencing data, researchers can reanalyze bulk RNA sequencing (bulk RNA-seq), microarray, methylation, mutation, copy-number, proteomic, single-cell, and spatial datasets generated by prior studies. This reuse allows independent groups to test hypotheses across cohorts, examine molecular subtypes, prioritize genes or pathways, infer immune and stromal composition, and generate candidate models for prognosis or therapy response.

The same resource base also exposes recurring weaknesses. Public-database studies often draw from overlapping cohorts, apply similar pipelines, and report statistically significant biomarkers or signatures without sufficient external validation or reproducible code. The difference between a candidate discovered in The Cancer Genome Atlas (TCGA) and a clinically useful biomarker is therefore substantial. A risk score that separates Kaplan-Meier curves in a retrospective dataset is not necessarily calibrated, externally transportable, or useful beyond established clinical predictors. A bulk immune-deconvolution estimate is not the same as direct histopathological or spatial evidence. A ligand-receptor pair inferred from single-cell expression does not prove functional signaling. These distinctions matter because public omics can accelerate translational research only when computational claims are matched to appropriate evidence. **Figure 1** and **Table 1** summarize the resource landscape and its main tradeoffs.

**Figure 1:**
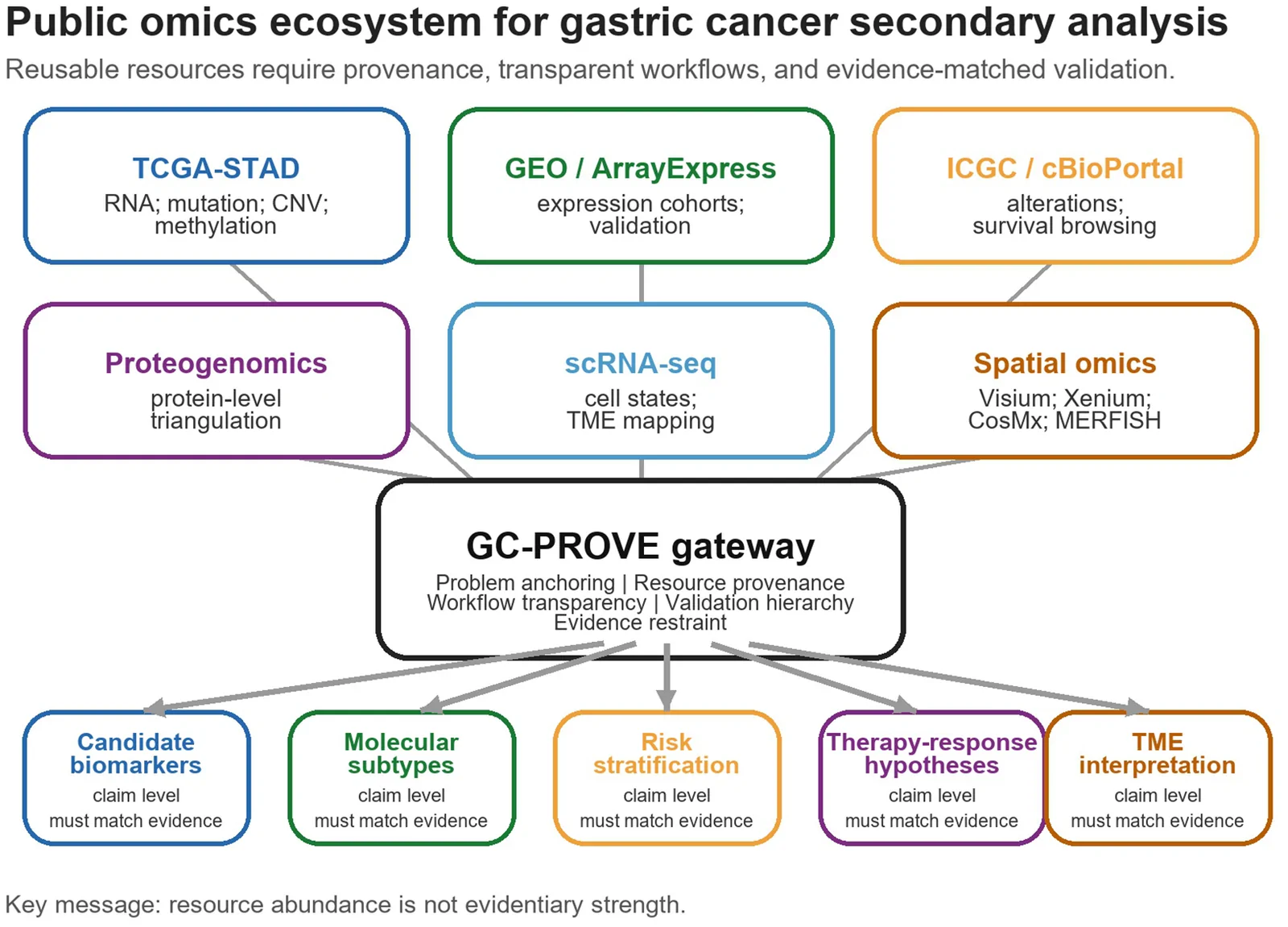
Public omics ecosystem for gastric cancer secondary analysis. Public resources enter a GC-PROVE gateway before supporting biomarker, subtype, prognostic, therapy-response, or tumor-microenvironment claims. The figure emphasizes that resource abundance is not equivalent to evidentiary strength. Abbreviations: GC-PROVE, problem anchoring, resource provenance, omics workflow transparency, validation hierarchy, and evidence restraint; TME, tumor microenvironment.

**Table 1:** Public databases and platforms for gastric cancer secondary analysis.

Resource or platform	Main data layers	Typical use	Key strength	Main limitation
TCGA-STAD	RNA-seq, mutation, copy-number variation, methylation, clinical annotation	Subtyping, differential expression, survival modeling, immune inference	Multi-layer molecular atlas	Limited treatment granularity and cohort-specific composition
GEO/ArrayExpress	Microarray, RNA-seq, methylation, selected scRNA-seq	External validation, meta-analysis, platform replication	Many independent cohorts	Heterogeneous preprocessing and metadata quality
Asian Cancer Research Group and other Asian cohorts	Expression and clinical outcome	Molecular subtype and survival context	Clinically informative subtype framework	Not always directly harmonized with TCGA pipelines
ICGC/cBioPortal	Mutation, copy-number variation, expression, survival browsing	Exploratory confirmation and alteration context	Accessible cross-study exploration	Web-tool analyses may be incompletely reproducible
Proteogenomic resources	Proteomics and phosphoproteomics	Protein-level pathway support	Biological triangulation beyond mRNA	Smaller cohorts and limited power for survival modeling
Single-cell repositories	scRNA-seq and cell annotations	Cell-state, lineage, TME, and communication analysis	Cellular resolution	Dissociation bias, batch effects, annotation uncertainty
Spatial platforms	Visium, Xenium, CosMx, MERFISH, Stereo-seq, imaging	Spatial validation and niche mapping	Tissue architecture	Limited regions, platform differences, complex preprocessing

Gastric cancer encompasses intestinal-type, diffuse-type, and mixed-type adenocarcinoma by Lauren classification; proximally and distally located tumors differ in etiology and prognosis; Asian and Western cohorts show substantial molecular and epidemiological differences; and the treatment landscape has evolved from surgery alone to perioperative chemotherapy, adjuvant therapy, and immune checkpoint blockade. TCGA-STAD and other public gastric cancer cohorts carry inherent limitations in treatment annotation, stage distribution, and geographic representation that secondary analysts must consider. Existing reviews have summarized gastric cancer molecular classification, precision oncology, transcriptomic signatures, single-cell applications, and spatial multi-omics (4–12). This review examines public-omics secondary analysis itself. It asks how public data are transformed into claims, where inference becomes vulnerable, and what standards could make future work more reliable.

We propose a practical organizing framework, GC-PROVE. The acronym emphasizes five requirements for more credible secondary analysis: problem anchoring, resource provenance, omics workflow transparency, validation hierarchy, and evidence restraint. Problem anchoring means that the analysis begins with a clinical or biological question rather than a fashionable gene set. Resource provenance means that datasets, sample filters, endpoints, preprocessing, and cohort overlap are auditable. Omics workflow transparency means that normalization, feature selection, batch correction, modeling, and sensitivity analyses are reported in sufficient detail for replication. Validation hierarchy means that computational findings are interpreted according to the level of evidence supporting them, from public discovery to external validation, tissue or protein support, functional testing, and clinical utility. Evidence restraint means that public-data associations are presented as hypotheses unless stronger validation is available. Section 2 describes the literature identification strategy; Section 3 catalogs reusable public data resources; Section 4 examines major analytical workflows using prognostic modeling as a stress test; Section 5 summarizes research applications; Section 6 analyzes methodological limitations; Section 7 proposes the GC-PROVE framework for future work; Section 8 discusses implications for authors, reviewers, and editors; and Section 9 concludes.

## 2 Literature identification strategy

This article is a methodological narrative review supported by reproducible database scans. It is not a systematic review, scoping review, or meta-analysis, and no PRISMA-style screening, dual-reviewer study selection, formal risk-of-bias scoring, or quantitative effect synthesis was performed. PubMed was searched on 2026-06–30 using combinations of gastric cancer terms with public database, TCGA, GEO, transcriptome, prognostic model, risk signature, immune infiltration, weighted gene co-expression network analysis (WGCNA), competing endogenous RNA (ceRNA), methylation, multi-omics, single-cell, spatial omics, machine learning, validation, and reproducibility terms. Searches covered 2019-01-01 through 2026-06-30. The core search strings are: (gastric cancer[Title/Abstract] OR stomach adenocarcinoma[Title/Abstract] OR stomach cancer[Title/Abstract]) AND (TCGA[Title/Abstract] OR GEO[Title/Abstract] OR public database[Title/Abstract] OR public datasets[Title/Abstract] OR bioinformatics[Title/Abstract] OR secondary analysis[Title/Abstract] OR transcriptome[Title/Abstract]); a second query replaced the second bracket with (risk signature[Title/Abstract] OR prognostic signature[Title/Abstract] OR prognostic model[Title/Abstract] OR nomogram[Title/Abstract] OR survival model[Title/Abstract]); parallel queries used single-cell, spatial omics, machine-learning, and reproducibility terms. The complete search strings, PubMed QueryTranslation outputs, retrieval dates, and sorting settings are provided in **Supplementary File S1**, and the search counts are provided in **Supplementary Table S1**.

References were selected to support a methodological narrative rather than to exhaustively enumerate every public-database study. Priority was given to foundational gastric cancer molecular resources, recent review articles, representative original studies across major workflow classes, gastric-cancer examples with external or biological validation, and methodological papers on prediction-model reporting, validation, batch effects, immune deconvolution, single-cell integration, cell-cell communication, and reproducible computational research. Studies were excluded from detailed discussion when they were unrelated to gastric cancer, lacked a clear public-omics component, repeated a common workflow without methodological relevance, or used language implying clinical readiness without adequate validation. Search strings, scan outputs, and supplementary comparison tables are provided with this submission; source text and generation scripts are retained by the authors and can be made available on reasonable request. OpenAI Codex (GPT-5; OpenAI/Codex desktop application) assisted with document parsing, language editing, figure and table preparation, formatting, and consistency checks; it did not generate patient-level data, determine database-scan counts, decide record inclusion or exclusion, or replace author review, literature appraisal, or PubMed verification. Scientific decisions, record appraisal, and PubMed verification remained with the authors, and the same use is declared formally in the Generative AI Declaration.

A supplementary public metadata scan using OpenAlex, Crossref, and Semantic Scholar was used only as a public metadata gap check (**Supplementary Table S4**). It was not an Embase or Web of Science search and should not be interpreted as a formal multi-database systematic search. The scan returned 135 deduplicated public-metadata records. **Supplementary Table S4** records PubMed indexing status, search-corpus membership, and topic eligibility as separate fields, because absence from the retrieved search corpus is not the same as absence from PubMed. On re-checking the records that the earlier scan had flagged as probably absent from PubMed, 121 of 135 proved to be indexed there, which is why these fields are now reported separately.

The PubMed scan was used to describe the volume of records returned by these queries. From 2019-01-01 through 2026-06-30, the search for public omics or bioinformatic secondary analysis in gastric cancer returned 4416 PubMed records, while the prognostic-signature/risk-model search returned 1812. Searches focused on single-cell and spatial gastric cancer omics returned 876 and 148 records, respectively, and the artificial intelligence (AI) or machine-learning public-omics prognosis search returned 636. These counts are query-dependent indicators, not systematic prevalence estimates; because the date window is fixed while PubMed continues to index records that fall within it, re-running the same queries at the revision date retrieves slightly more records (4,460, 1,840, 904, 159, and 662, respectively). Both sets of counts, and the complete PMID-level record sets behind them, are reported in **Supplementary File S2**. For the prognostic query, the re-run returned 1,840 records; 1,839 were parsed successfully for publication dates and one record could not be parsed. The difference between the 1,840 records returned and the 1,839 parsed is therefore a single unparsed record, and the record-level results for all of them are given in **Supplementary File S2**. Because a record can carry both an electronic publication year and a print publication year, the sum of the yearly values in **Supplementary Table S2** exceeds the total returned by the date-bounded query. The excess is of similar magnitude across all topics (7.7% for the prognostic query, 8.4% for single-cell, 9.5% for spatial, and 8.5% for the AI or machine-learning query), which is consistent with dual year assignment rather than a difference in query scope. This was tested directly rather than inferred or asserted. Of the 1,840 MEDLINE records returned by the prognostic query, 1,839 were parsed for publication dates and one record (PMID 36343147) could not be parsed; it is listed with a null year in the record-level data file. Of the 1,839 parsed records, 1,592 carry both an electronic publication year (ArticleDate) and a print or issue year (PubDate), while 247 carry only a print year. In 147 of the 1,592 records that carry both years the two years differ, which is 9.2% of those 1,592 records and 8.0% of all 1,839 parsed records. Counting each record once in its print year and again in its electronic year when the two differ gives a yearly total of 1,839 + 147, an excess of 8.0% over the date-bounded count, which matches the excess actually observed in the yearly series (7.7%). The PMID, electronic year, print year, MEDLINE entry year, and a differ/agree flag for every record are given in **Supplementary File S2**_year_assignment_record_level.csv in **Supplementary File S2**, so that the calculation can be reproduced record by record. The year-assignment rule, the per-topic record counts, and complete PMID-level record sets for every topic query are provided in **Supplementary File S2**. The complete search strings and their PubMed QueryTranslation outputs are provided in **Supplementary File S1**. The scans were used to contextualize the narrative and identify illustrative reporting gaps; they were not used to estimate field-wide prevalence, evaluate study quality, or support quantitative conclusions. A separate title/abstract-level screen was then performed on the 100 most recent PubMed records returned by the prognostic-signature query, sorted by date through 2026-06-30. This query deliberately does not require public-data reuse or omics analysis, so the screened records represent broad prognosis literature in gastric cancer and also include clinical, imaging, and cost-effectiveness studies. The screen therefore describes a broad prognosis-literature corpus rather than the prevalence of methodological practices within gastric cancer public-omics research. Within that corpus, mentions detected in titles or abstracts were as follows: external-validation mentions in 46/100 records, calibration in 36/100, decision-curve or net-benefit in 33/100, clinical-comparator in 67/100, experimental-validation in 30/100, and code-availability in 0/100. These values are illustrative text-mining signals about what is mentioned in titles and abstracts; they are not formal quality ratings and do not establish that the corresponding analyses were performed adequately. The 100 records were coded with predefined yes/no rules by a single author without duplicate coding. The absence of code-availability mentions should be interpreted as an abstract-level lower bound, because code statements usually appear in full-text declarations rather than abstracts; zero mentions do not indicate that no code exists. The screened records are listed in **Supplementary Table S3**, and the coding rules, extraction procedure, retrieval date, and sorting settings are documented in **Supplementary File S3**.

Supportive publication trend data are provided as **Supplementary Figure S1** and **Supplementary Table S2**.

Supportive reporting-signal data are provided as **Supplementary Table S3**.

The per-topic counts in **Supplementary Table S1** and the term mentions in **Supplementary Table S3** are not directly comparable, because they describe different samples under different coding definitions and different denominators. For **Supplementary Table S1**, the sample is the 100 most recent PubMed records returned by each topic query and the denominator is 100; the column records how many of those 100 records are themselves studies of that topic. For the prognostic row the value is therefore 0, because none of the 100 most recent prognostic records is primarily an external-validation study. For **Supplementary Table S3**, the sample is the 100 most recent records returned by the prognostic query alone and the denominator is again 100; the column records how many records use the corresponding term anywhere in the title or abstract, so the external-validation value is 46. A record can therefore mention external validation without being an external-validation study. The two results answer different questions — whether a paper is about external validation, and whether a paper talks about it — and the samples, coding definitions, and denominators for both are set out in **Supplementary File S3** so that either result can be reproduced.

A scope boundary between descriptive signals and formal quality assessment should be stated explicitly, because the two are easily conflated. The database scans reported here (**Supplementary Tables S1**– **Supplementary Tables S4**, **Supplementary Tables S7**, **Supplementary Figure S1**) are descriptive. They are produced by fixed queries run over a fixed date window, they are aggregated over record sets rather than over individual studies, they depend on query phrasing and on PubMed indexing status at the retrieval date, and the title/abstract-level coding was performed by a single author without duplicate coding. They describe what a defined corpus returns and what its titles and abstracts mention. They therefore cannot be used to rank, score, or judge any individual study, they do not establish whether a reported analysis was performed correctly or completely, and no scan value in this review should be read as a quality rating of any specific paper discussed in Sections 3-5. Formal quality assessment is a different activity: it is applied study by study, it requires reading the full text, it uses structured instruments such as PROBAST for risk of bias and TRIPOD or TRIPOD+AI for reporting completeness, and it is ideally performed by more than one assessor. This review does not perform formal quality assessment of the literature it discusses. The per-study appraisals in Section 7 are likewise author judgments recorded against the GC-PROVE domains rather than validated risk-of-bias scores, and each is reported with the specific source features on which it rests so that readers can accept or reject it.

## 3 Public data resources for gastric cancer secondary analysis

### 3.1 Bulk genomic and transcriptomic resources

The TCGA stomach adenocarcinoma cohort (TCGA-STAD) remains the most frequently reused multi-layer resource for gastric cancer. Its value lies in linking gene expression, mutation, copy-number alteration, DNA methylation, and clinical annotation in a publicly accessible framework (2). TCGA also established a widely used molecular classification, including Epstein-Barr virus-positive, microsatellite instability, genomically stable, and chromosomal instability subtypes. The Asian Cancer Research Group classification provided an additional clinically informative subtype framework, including groups associated with distinct outcomes (3). These resources remain foundational, but secondary analysts should remember that they were not designed to answer every downstream question. Treatment details, follow-up completeness, geographic representation, histologic composition, and endpoint definitions may not match the intended analysis.

A compact overview of the major data platforms and their limitations is given in **Table 1**.

The Gene Expression Omnibus (GEO) and related expression repositories are essential for external replication and cross-platform comparison (13). GEO contains many gastric cancer microarray and RNA-seq datasets, which can be used for differential expression, survival validation, immune deconvolution, pathway scoring, and meta-analysis. The advantage is breadth; the limitation is heterogeneity. Platforms, probe annotations, preprocessing pipelines, tissue sources, clinical metadata, and endpoint quality differ substantially across datasets. For this reason, a validation cohort from GEO is helpful but not automatically definitive. A strong study should describe accessions, inclusion criteria, normalization, gene mapping, missingness, endpoint definitions, and whether the validation cohort was chosen before or after model development.

The International Cancer Genome Consortium (ICGC), cBioPortal, and other public portals facilitate rapid exploration of mutation, copy-number, expression, and survival associations (14, 15). They support hypothesis prioritization and visual confirmation. The risk is that web-based exploration can produce a fragmented evidence trail. A candidate gene may be checked in several portals, but the exact sample filters, data versions, survival endpoints, and statistical settings may be difficult to reproduce. Public interfaces are best used as exploratory tools unless the underlying data and code are documented.

### 3.2 Proteomic, epigenomic, and multi-layer resources

Transcriptomic associations often require protein-level or epigenomic triangulation. DNA methylation and epigenomic analyses have been used to study preneoplastic lesions, circulating tumor DNA, viral-host genome interaction, subtype markers, early-detection candidates, and prognostic signals in gastric cancer (16–18). These analyses are mechanistically appealing because methylation can regulate gene expression and reflect cell lineage, microbial, viral, or tumor-state changes. Yet methylation markers are tissue-, platform-, and cell-composition-dependent, and expression correlation does not prove causality. Better epigenomic secondary analyses integrate methylation, expression, clinical context, and independent validation rather than treating methylation status as a stand-alone mechanism.

Proteogenomic resources add another layer of evidence. Transcript abundance is not always proportional to protein abundance, and many candidate biomarkers need tissue or protein confirmation before translational interpretation. Proteomics and phosphoproteomics can clarify pathway activation, post-transcriptional regulation, and druggable signaling states (19). Their limitations include smaller sample sizes, incomplete clinical annotation, and potential batch effects. In the GC-PROVE hierarchy, proteomic support does not by itself prove clinical utility, but it strengthens biological plausibility when aligned with transcriptomic and clinical evidence.

### 3.3 Single-cell, spatial, and foundation-model resources

Single-cell RNA sequencing (scRNA-seq) has changed the granularity of gastric cancer secondary analysis. Instead of treating a tumor as a bulk mixture, single-cell studies can map malignant epithelial states, fibroblast subsets, myeloid programs, T-cell exhaustion, endothelial phenotypes, metastatic niches, and premalignant transitions (20–25). These datasets help interpret bulk signatures and the tumor microenvironment (TME). A poor-prognosis bulk gene set may reflect malignant cells, inflammatory cells, fibroblasts, stromal abundance, or changing tumor purity. Single-cell evidence can help locate the cellular source of a signal and prevent overinterpreting a bulk association as tumor-intrinsic.

Spatial omics extends single-cell logic by preserving tissue architecture. Recent gastric cancer spatial studies and reviews show how spatially resolved transcriptomics and imaging can map tumor glands, invasive fronts, fibroblast-macrophage interactions, metabolic niches, and immune-suppressive microenvironments (11, 12, 26–29). Technologies such as Visium, Xenium, CosMx, MERFISH, Stereo-seq, and multiplex imaging differ in resolution, gene coverage, throughput, and tissue requirements. For this review, spatial data are treated as a context and validation layer. They can test whether signals inferred from bulk or single-cell data are anatomically plausible, but limited tissue regions and platform-specific preprocessing still constrain generalizability.

Foundation models such as Geneformer, scGPT, scFoundation, and Nicheformer suggest that large-scale pretraining may support cell-state embedding, annotation, and integration (30–34). Their relevance to gastric cancer public-omics is prospective. Published evaluations of these models have focused largely on cell-type annotation, batch integration, and perturbation prediction in benchmark atlases. The evaluation tasks reported in these studies are mainly cell-type annotation, batch integration, and perturbation prediction, assessed on benchmark atlases such as CellxGene, Tabula Sapiens, and the Human Cell Atlas. Several evaluations report limited zero-shot performance relative to transparent task-matched comparators, including marker-gene scoring, principal-component analysis followed by k-means or Leiden clustering, and logistic regression on highly variable genes. A further limitation is that the pretraining corpora of these models may overlap the evaluation atlases, so the evaluation data are not always unseen and transferability can appear greater than it is. These models may help reuse large single-cell repositories, but their contribution should be judged against transparent, task-matched baselines on data not used during pretraining. Foundation-model output should not substitute for external validation, interpretability, or biological testing.

## 4 Major analytical workflows

### 4.1 Differential expression, enrichment, and meta-analysis

Differential expression analysis remains the entry point for many public-omics studies. Typical workflows compare tumor with adjacent tissue, subtype with subtype, high-risk with low-risk groups, or treatment-related phenotypes, followed by enrichment analysis and candidate-gene prioritization. Its main advantages are interpretability and relative ease of cross-dataset validation. Its weakness is that differential expression can reflect tumor purity, stromal abundance, sample processing, inflammation, or histologic composition rather than causal tumor biology. A differentially expressed gene should therefore be treated as a candidate signal, not a driver or clinical biomarker.

Meta-analysis across GEO cohorts can improve consistency across cohorts, but only if platform differences and cohort definitions are handled carefully. Probe-to-gene mapping, duplicate probes, batch effects, normalization, and missing clinical variables can change results. Batch-correction tools such as ComBat and surrogate variable analysis are useful but require appropriate design matrices and transparent reporting (35, 36). Analysts should report sensitivity analyses across cohorts rather than relying only on a pooled estimate.

### 4.2 Prognostic modeling, risk signatures, and AI as a stress test

Prognostic modeling is the central case study for this review because it is common, clinically attractive, and methodologically vulnerable. Many gastric cancer public-database studies follow a recognizable template: choose a biological theme, collect candidate genes, screen features by univariate Cox regression, select variables using the least absolute shrinkage and selection operator (LASSO)-Cox, stepwise regression, random forest, XGBoost, or another machine-learning method, construct a risk score, divide patients into high- and low-risk groups, and report Kaplan-Meier curves, time-dependent area under the receiver operating characteristic curve (AUC), the concordance index, nomograms, calibration, and sometimes decision-curve analysis. Recent gastric cancer examples include hypoxia-, mitochondrial-, pyroptosis-, lncRNA-, microbiome-, migrasome-, metabolism-, and single-cell-informed signatures (37–47).

The recurrent failure modes that warrant early reviewer attention are summarized in (**Box 1**). The number of published signatures is not the same as cumulative progress. A new signature may be statistically significant while adding little to clinical knowledge if it is built from overlapping cohorts, weakly validated, biologically vague, or not compared with TNM stage and other clinical predictors. The Transparent Reporting of a multivariable prediction model for Individual Prognosis Or Diagnosis (TRIPOD) statement, its TRIPOD+AI extension, the Prediction model Risk Of Bias ASsessment Tool (PROBAST), and modern prediction-model validation guidance are directly relevant to these studies (48–52). Discrimination, calibration, and clinical utility are separate properties. A high AUC does not guarantee accurate risk estimation, and accurate risk estimation does not guarantee decision-relevant benefit. Decision-curve analysis helps evaluate net benefit, but it must be interpreted in a clinically meaningful context (53).

Box 1 **Thirteen red flags in public-omics manuscripts.**

1. No clearly stated biological or clinical question before pipeline execution.

2. Unclear dataset accessions, sample filters, endpoint definitions, or cohort overlap.

3. Feature selection, cutoff selection, batch correction, or hyperparameter tuning performed outside a locked training workflow.

4. External validation described, but the model is rebuilt or reselected in the validation cohort.

5. Discrimination metrics reported without calibration, decision-curve analysis, or clinical comparators.

6. Risk groups defined by *post hoc* optimal cutoffs without sensitivity analysis.

7. Co-expression modules from weighted gene co-expression network analysis (WGCNA) interpreted as regulatory mechanisms without independent replication.

8. Competing endogenous RNA (ceRNA) networks interpreted as established regulation without direct binding, stoichiometry, or perturbation evidence.

9. Cell-cell communication inferred from ligand-receptor co-expression and interpreted as measured signaling.

10. Immune or stromal deconvolution estimates interpreted as immune function.

11. Single-cell or spatial data used illustratively rather than inferentially, without source attribution or tissue-level validation.

12. No code, package versions, model formula, coefficients, feature list, or random seed.

13. Conclusions imply clinical utility despite retrospective public-data evidence only.

Artificial intelligence and machine-learning methods extend these requirements rather than relax them. Random forests, support-vector machines, XGBoost, deep learning, multimodal models, and foundation-model-derived embeddings may improve nonlinear representation or integrate heterogeneous data layers. However, flexible algorithms also increase the need for separated feature selection, nested resampling during tuning, transparent hyperparameters, calibration assessment, comparison with clinical predictors, locked external validation, and reproducible code. In this review, AI is therefore held to the same requirements as any other public-omics workflow, with four additional obligations. First, the feature set must be fixed before any external data are seen. Second, hyperparameter tuning must be nested inside the training data rather than performed on the full dataset. Third, the training, tuning, and test partitions must be reported explicitly so that a reader can reconstruct them. Fourth, a simpler baseline model must be reported alongside the complex model, so that any gain in discrimination or calibration can be attributed to the method rather than to added flexibility.

This generic workflow is summarized in **Figure 2**, which also shows the decision points that determine whether the analysis remains interpretable. Three of these points are worth stating explicitly. First, development and validation data should be separated at the patient level, not at the sample or cell level, so that no individual contributes to both. Second, any data-dependent transformation, including normalization parameters, batch correction, imputation, and feature scaling, should be estimated on the training data and then applied unchanged to validation data; where model selection is performed, tuning should use nested resampling within the training data. Third, an untouched external validation, in which the locked model is applied to a new cohort, should be distinguished from subsequent recalibration or model updating, which uses the validation data and therefore no longer constitutes an external test. Biological interpretation and functional experiments are not compulsory stages for every prognostic model; their role depends on the intended claim and should be linked to it explicitly. The main failure modes addressed by these points are shown in **Figure 3**.

**Figure 2:**
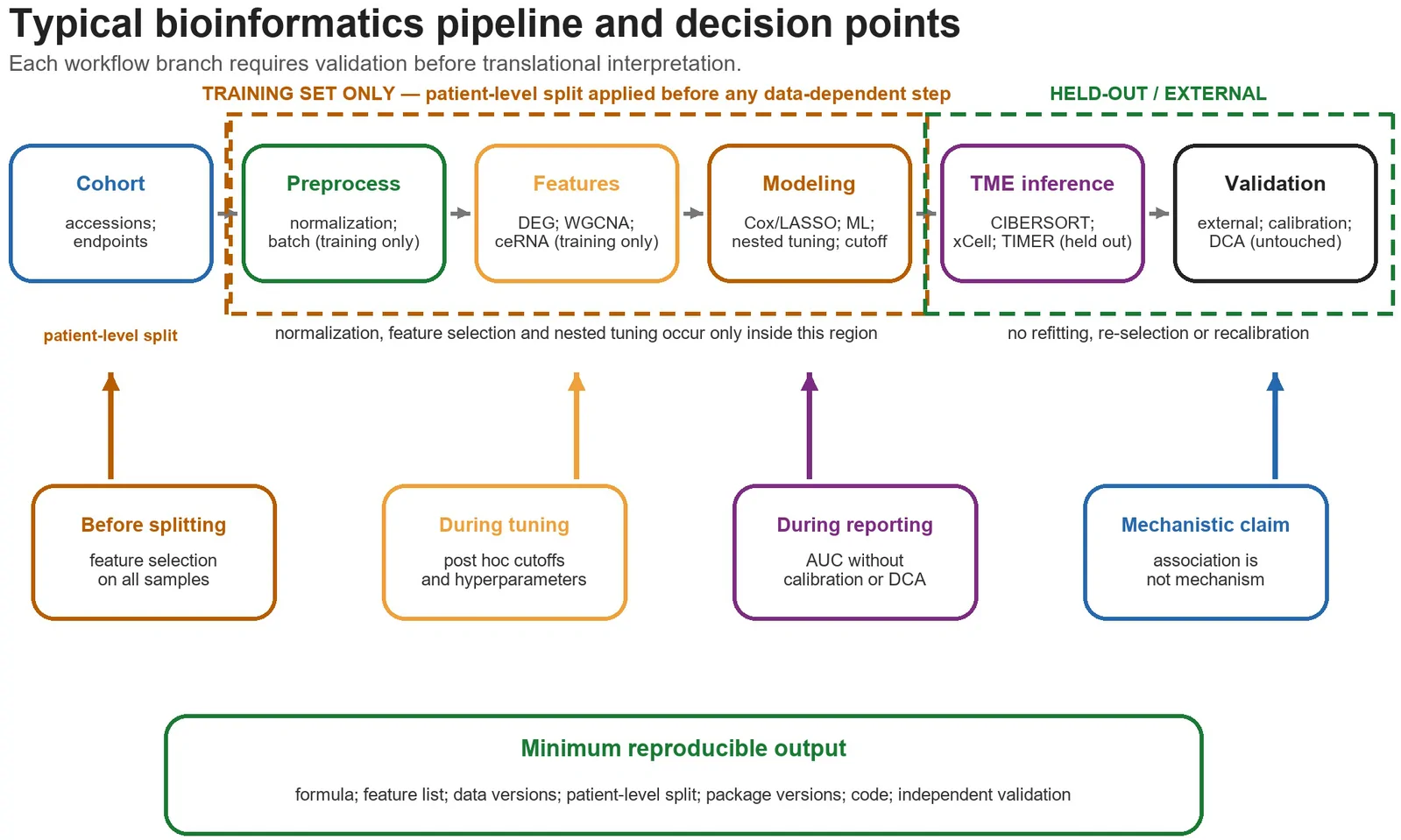
Typical bioinformatics pipeline and decision points. Public-omics workflows move from cohort definition and preprocessing to feature discovery, modeling, immune inference, validation, and mechanistic interpretation. The dashed regions mark the two data partitions. The training region contains every data-dependent step — normalization, feature selection, and hyperparameter tuning — and the patient-level split is applied before any of them, so that no information from held-out patients enters preprocessing, feature selection, or tuning. Tuning is nested inside the training data rather than performed on the full dataset. The held-out and external region is untouched by training: no refitting, feature re-selection, or recalibration should be applied there, so that external validation remains genuinely external. The figure also highlights where leakage, cutoff optimism, incomplete performance reporting, and biological overinterpretation can enter.

**Figure 3:**
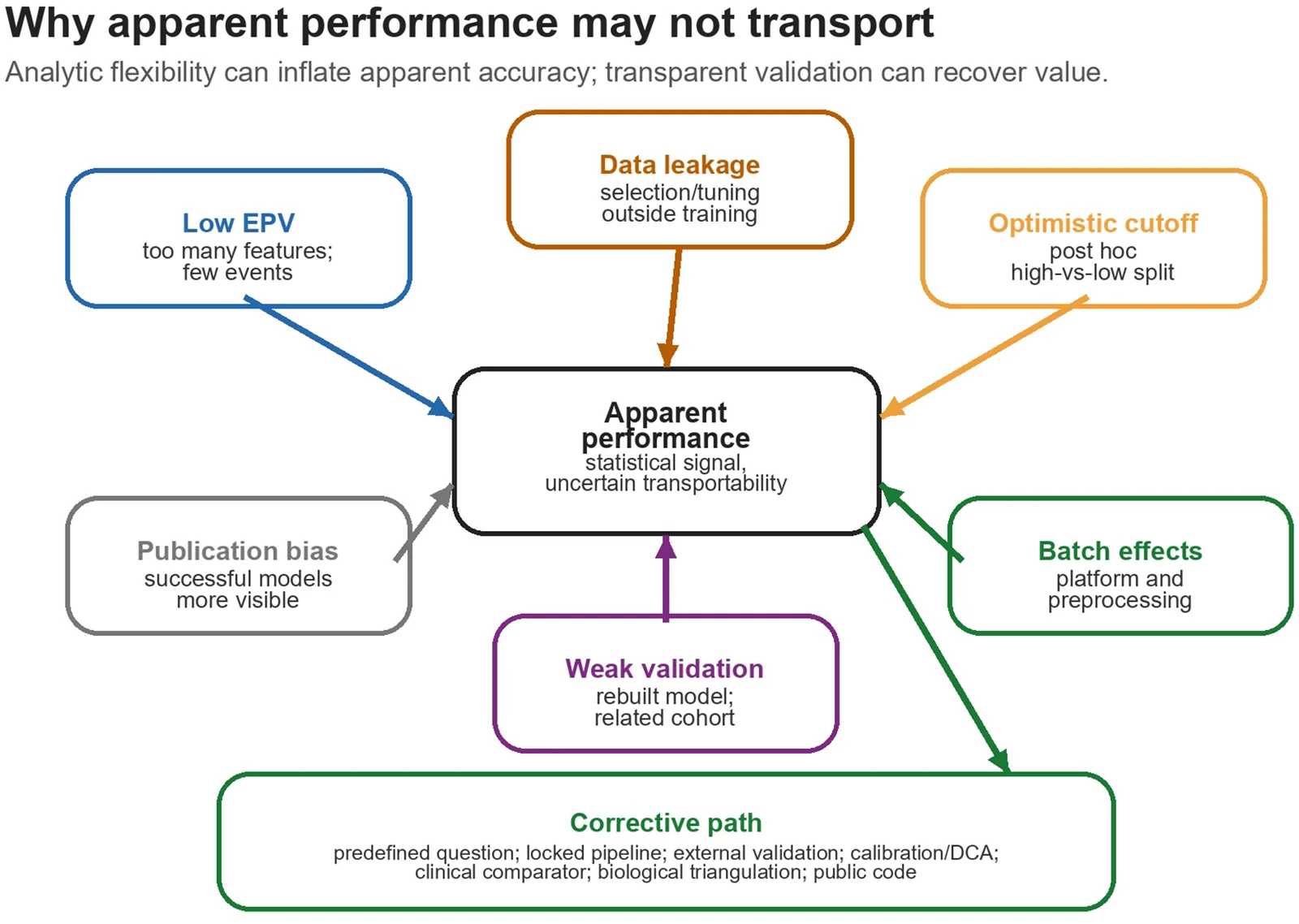
Why apparent performance may not transport. Common failure modes include low events per variable, data leakage, optimistic cutoffs, batch effects, weak validation, and publication bias. Corrective strategies include predefined questions, locked training pipelines, calibration, decision curves, clinical comparators, biological triangulation, and public code.

### 4.3 Case deconstruction: a representative workflow derived from public data

A representative gastric cancer risk-signature study can be dissected into six checkpoints. First, the training cohort is defined, often from TCGA-STAD, and candidate features are selected from a biological theme such as pyroptosis, hypoxia, immune infiltration, metabolism, or single-cell marker genes. Second, univariate Cox screening reduces the candidate set. Third, LASSO-Cox or another selection method constructs a smaller model. Fourth, a risk score is calculated as the weighted sum of selected features. Fifth, an optimal cutoff divides patients into high- and low-risk groups. Sixth, the model is evaluated in TCGA and one or more GEO cohorts, sometimes with a nomogram, calibration curve, and immune-infiltration analysis.

Each checkpoint can introduce optimism. If feature filtering is performed before train-test separation, validation information may leak into model development. If the cutoff is chosen after inspecting survival separation, Kaplan-Meier curves can exaggerate performance. Risk scores should preferably be evaluated as continuous predictors; when dichotomization is used, the threshold should be prespecified or justified, and validation cohorts should apply the cutoff defined in the training set without re-optimization. If multiple gene themes are tried but only the successful one is reported, publication bias becomes invisible. If the external cohort is used to adjust preprocessing, choose genes, or tune the cutoff, it is no longer a clean validation cohort. Re-estimating coefficients, reselecting features, or recalibrating thresholds in the validation cohort converts validation into redevelopment. The safeguards summarized in (**Box 2**) should be treated as minimum expectations. If the model is not compared with clinical predictors, its incremental value remains unclear. If code, coefficients, and package versions are unavailable, independent reproduction becomes difficult. This deconstruction is not meant to discredit every risk signature; it shows why prognostic modeling is a stress test for the entire public-omics field.

Box 2 **Key cautions for prognostic-signature claims.**

This box summarizes the cautions developed in Section 4.2-4.4 as a short checklist; the detailed rationale is given there.

1. Lock the analysis. Feature selection, tuning, and threshold determination should occur inside the training data only.

2. Do not dichotomize by default. Evaluate risk scores as continuous predictors; if high/low groups are used, prespecify the threshold and reuse it unchanged in validation.

3. Keep validation untouched. Re-estimating coefficients, reselecting features, or recalibrating thresholds in the validation cohort converts validation into redevelopment.

4. Report what the model adds. Discrimination alone is insufficient; report calibration, decision-curve analysis, and comparison with tumor-node-metastasis (TNM) stage and other clinical predictors.

5. State the evidence level. A retrospective public-data association supports hypothesis prioritization, not clinical use.

A representative comparison of study types and methodological roles is given in **Table 2**.

**Table 2:** Representative resources, studies, and methodological roles in the review.

Study or resource	Data/resource type	Methodological role	What it contributes	Caution for interpretation
TCGA gastric adenocarcinoma (2)	Multi-layer public atlas	Foundational subtype and resource context	Molecular classification and reusable omics layers	Not designed for all treatment-response questions
Asian Cancer Research Group molecular classification (3)	Expression and outcome cohort	Subtype/outcome comparator	Clinically informative subtypes	Requires harmonization with other datasets
Parallel single-cell and bulk analyses (71)	scRNA-seq plus bulk transcriptome	TME triangulation	Links cell states to bulk features	Cell annotation and cohort scale remain limiting
Spatially resolved multi-omics (11, 12, 26–29)	Spatial transcriptomics/proteomics	Spatial validation layer	Localizes metabolic and microenvironmental programs	Tissue-region coverage and platform effects matter
Gastric cancer spatial CAF studies (27, 29)	Spatial plus functional evidence	Mechanistic TME example	Links fibroblast states to immune modulation	Needs context-specific validation
Pyroptosis/single-cell-informed signature (40, 43)	Bulk plus single-cell integration	Prognostic-model case	Shows common modern signature pipeline	Requires locked external validation and clinical comparators
AI multimodal gastric cancer model (47)	Omics/AI integration	Emerging machine-learning example	Demonstrates richer multidimensional modeling	Higher reporting and validation burden
TRIPOD/TRIPOD+AI/PROBAST (48–50)	Reporting and risk-of-bias standards	Evaluation framework	Defines what transparent prediction modeling should report	Standards must be operationalized, not merely cited

### 4.4 Why apparent performance may not translate into clinical utility

Many signatures derived from public data do not generalize because the statistical and biological tasks are harder than the publication template suggests. The proportional-hazards assumption is frequently untested in signature studies despite its relevance for Cox models. Low events per variable (EPV) increase overfitting, and optimism-corrected internal validation (bootstrap or cross-validation) should supplement naïve performance estimates. Calibration slope, Brier score, and the concordance index with confidence intervals provide a more complete picture than any single metric. High-dimensional omics data make feature selection unstable. Batch effects can make a gene appear prognostic in one platform but not another. Optimistic cutoffs can transform continuous noise into striking survival curves. Repeated testing across gene sets, endpoints, and datasets can produce apparently coherent models that are fragile outside the discovery context. Publication bias then preferentially displays successful signatures while failed replications remain unpublished.

Public cohorts frequently differ in survival endpoints (overall survival, disease-free survival, recurrence-free survival, progression-free survival, disease-specific survival, and peritoneal recurrence), censoring mechanisms, follow-up duration, and treatment exposure; therefore, validation should apply the same endpoint definition whenever possible rather than treating all survival variables as interchangeable. Clinical translation requires more than statistical separation. A model should improve risk estimation beyond tumor-node-metastasis (TNM) stage, histology, performance status, treatment variables, and established molecular subtypes when those data are available. It should be calibrated in independent cohorts and should have a plausible intended use: surveillance intensity, trial stratification, treatment selection, or biological prioritization. Many models derived from public data are more appropriate for hypothesis generation than for clinical decision support. Public-data-derived signatures can still be useful when they define a clear question, preserve a locked model, demonstrate transportability, and provide biological or clinical evidence proportional to the claim.

### 4.5 Immune infiltration and tumor microenvironment inference

Immune and stromal inference has become central to gastric cancer public-omics analysis. ESTIMATE, CIBERSORT, xCell, TIMER, and related tools estimate tumor purity, immune infiltration, stromal content, and cell-type composition from bulk expression data (54–58). These tools are useful for generating tumor-microenvironment hypotheses, particularly when paired with subtype, survival, or therapy-response analyses. They also connect naturally to single-cell and spatial data, which can localize inferred immune or stromal programs to specific cell states and tissue regions.

The limitation is that deconvolution estimates are model-based, not direct measurements. Different tools can produce different estimates because they use different reference signatures, assumptions, and normalization procedures. Tumor purity, necrosis, stromal abundance, and platform effects can alter results. Immune checkpoint expression does not equal response to immune checkpoint blockade, and an inferred T-cell fraction does not reveal exhaustion, spatial exclusion, antigen presentation, or suppressive myeloid context. TME claims become stronger when bulk deconvolution is triangulated with single-cell data, spatial transcriptomics, multiplex imaging, immunohistochemistry, or clinically annotated immunotherapy cohorts.

### 4.6 WGCNA, ceRNA networks, methylation, and multi-omics

WGCNA and network-based analyses identify co-expression modules and hub genes associated with clinical traits or inferred immune states (59, 60). They are useful for module prioritization, but correlation does not establish regulation. Module preservation, independent replication, and biological triangulation are important. A module associated with immune infiltration is more credible if its genes map to immune or stromal cell types in single-cell data and to relevant tissue niches in spatial data.

Non-coding RNA and ceRNA analyses are another common workflow (61). These studies often construct lncRNA-miRNA-mRNA or circRNA-miRNA-mRNA axes from expression correlations and target databases. The biological idea is appealing, but stoichiometry, subcellular localization, RNA abundance, and direct binding are rarely established in public-data studies. ceRNA networks should therefore be framed as regulatory hypotheses requiring experimental validation, not as proven mechanisms.

Multi-omics integration can connect mutation, copy number, methylation, expression, proteomics, and immune context. Its value is highest when integration clarifies biology that a single layer cannot explain. Its weakness is opacity. Different algorithms handle missingness, scaling, feature selection, and cohort structure differently. Multi-omics papers should report why integration was needed, how missing data were handled, whether results replicate across cohorts, and whether the integrated model improves interpretation or prediction beyond simpler models.

### 4.7 Single-cell and spatial reanalysis

Single-cell and spatial reanalysis are increasingly important because they can resolve the cellular and anatomical origin of bulk signals. Single-cell atlases have mapped premalignant programs, lineage states, metastatic niches, fibroblast diversity, myeloid phenotypes, and immune suppression in gastric cancer (20–25). Spatial studies add tissue context, identifying where tumor-stromal and immune interactions occur (26–29). Tools such as CellChat and related ligand-receptor frameworks are useful for prioritizing communication hypotheses (62, 63).

These methods should not be treated as automatic validation. Dissociation can alter cell representation, and ambient RNA as well as doublets may distort cell-state interpretation. Integration can remove real biological variation or create artificial alignment. Cell annotation is partly subjective, and patient-level variation is obscured when thousands of cells are treated as independent biological replicates (pseudoreplication). Trajectory methods imply developmental order but do not prove lineage. Cell-cell communication tools infer potential interactions from co-expression and ligand-receptor databases; they do not directly measure signaling. Spatial data improve context but may be affected by region-of-interest selection, limited section depth, tissue processing artifacts, and platform-specific resolution constraints. The strongest use of high-resolution data in this review is triangulation: single-cell and spatial evidence can contextualize bulk signatures and prioritize mechanisms for validation.

Prognostic signature studies are compared in **Supplementary Table S5**, which records the theme, development cohort, validation design, endpoint, patient independence, clinical comparison, and reported performance assessment for each study as verified against the full text of the cited article; each entry also gives the source location within that article and its PubMed identifier. That table shows that validation designs differ substantially within this literature: some studies perform external validation in independent GEO cohorts, one performs validation only through a random training/testing split of the same TCGA cohort, and one uses a single-institution discovery and validation cohort rather than TCGA. The supplementary package, including the search logs, the PMID-level record sets, the coding rules, and the checklist, is archived at https://doi.org/10.5281/zenodo.23066111 (concept DOI, resolving to the latest version). Comparisons of validation quality should therefore be made at the level of individual study designs rather than by theme.

A detailed comparison of single-cell and spatial methods in gastric cancer is provided as **Supplementary Table S6**.

## 5 Applications in gastric cancer research

Public-omics secondary analysis has contributed to five broad application areas. First, it supports biomarker discovery. Differential expression, methylation, ncRNA, proteomic, single-cell, and spatial analyses can nominate diagnostic, prognostic, predictive, or therapeutic candidates. The term biomarker should be used carefully. Most public-database candidates are not validated clinical biomarkers; they are candidate markers requiring replication, protein-level confirmation, functional evidence, and assessment in clinically relevant specimens.

Several gastric cancer markers illustrate how far a public-omics candidate can be from clinical use, but they differ in intended use and in the strength of the supporting evidence. Treatment-selection markers with approved indications include HER2 amplification for anti-HER2 therapy and microsatellite instability-high (MSI-H) or mismatch repair deficiency (dMMR) for immune checkpoint blockade; programmed death-ligand 1 (PD-L1) combined positive score (CPS) is used to guide checkpoint blockade in defined settings. Claudin 18.2 (CLDN18.2) is a treatment-selection marker with a defined assay and cutoff in approved indications, and fibroblast growth factor receptor 2b (FGFR2b) remains largely investigational in gastric cancer. Epstein-Barr virus (EBV) status is primarily a molecular-subtype and prognostic classifier rather than a treatment-selection marker. For each marker, the treatment setting, assay, cutoff, jurisdiction, and evidence date determine clinical utility, and none of these can be inferred from a public-omics association alone. Several further distinctions are essential for evidence restraint: a candidate marker is not a validated clinical biomarker; a prognostic association is not a predictive marker unless it is linked to treatment benefit; an association observed in treated patients, or a response demonstrated experimentally, does not by itself establish treatment-effect prediction, which requires an appropriate treatment comparator and a justified interaction analysis; an immune-infiltration estimate is not immune function; and a ligand-receptor inference is not proof of signaling. Decision-curve analysis should be tied to a defined clinical decision and a meaningful threshold range rather than reported as a generic metric. **Table 3** maps common claim types to the minimum evidence required and to examples of unsafe claims; it distinguishes a treatment-response association from treatment-effect prediction as separate claim types with different minimum evidence. The marker-level details are as follows, with each value reflecting the evidence reviewed for this article as of 2026. HER2 amplification is used for treatment selection in advanced or metastatic gastric and gastro-esophageal junction adenocarcinoma; it is assessed by immunohistochemistry with *in-situ* hybridization confirmation; the cutoff is IHC 3+, or IHC 2+ with ISH positivity; and the marker is approved for this indication by the FDA, the EMA, and the NMPA. MSI-H or dMMR status is used for treatment selection in advanced solid tumors including gastric cancer; it is assessed by polymerase chain reaction, next-generation sequencing, or immunohistochemistry; because the result is categorical there is no numerical cutoff; and it is approved in the same jurisdictions. PD-L1 combined positive score is used to guide checkpoint blockade in defined advanced or metastatic settings; it is assessed by immunohistochemistry with the 22C3 or 28–8 assay; the cutoff depends on the regimen and the jurisdiction, and is generally a combined positive score of at least 1 or at least 5; and it is approved by the FDA, the EMA, and the NMPA. Claudin 18.2 is used for treatment selection in first-line HER2-negative advanced gastric and gastro-esophageal junction adenocarcinoma; it is assessed by immunohistochemistry; the cutoff is moderate-to-strong membranous staining in at least 75% of tumor cells; and it is approved in Japan and China, with regulatory review continuing in other jurisdictions. FGFR2b remains investigational in gastric cancer, and no assay or cutoff is yet standardized. Epstein-Barr virus status is a molecular-subtype and prognostic classifier rather than a treatment-selection marker. The corresponding grid, recording treatment setting, assay, cutoff, jurisdiction, and evidence date for every marker, is provided as **Supplementary Table S7**.

**Table 3:** Claim type vs. minimum evidence in gastric cancer public-omics secondary analysis.

Claim type	Typical public-omics output	Minimum responsible evidence	Unsafe claim
Candidate biomarker	Differential expression, methylation, or protein association	Independent cohort replication; tissue or protein support	“Validated clinical biomarker”
Prognostic signature	Risk score, Kaplan-Meier separation, AUC	Locked external validation; calibration; clinical comparator; decision-curve analysis	“Clinically useful prediction model”
Treatment-response association	Association with a drug-response signature or with outcome in treated patients	Treatment-exposed cohort; pre-specified analysis; experimental validation of the marker	“Predicts response to therapy” stated from a single-arm or retrospective association
TME inference	CIBERSORT, xCell, or TIMER deconvolution estimates	Cross-tool sensitivity analysis; scRNA-seq, spatial, or immunohistochemistry support	“Immune mechanism proven”
ceRNA network	Expression correlations plus target-database predictions	Direct binding evidence; perturbation; stoichiometry; localization data	“Regulatory axis confirmed”
Cell-cell communication	Ligand-receptor co-expression	Spatial proximity; protein evidence; functional perturbation	“Functional signaling demonstrated”
Foundation-model output	Embeddings or in silico predictions	Benchmarking against transparent baselines; external transfer validation	“AI discovers biological mechanism”
Treatment-effect prediction	A marker-by-treatment interaction estimated in a randomized or otherwise treatment-comparable setting	Randomized comparison or a justified interaction analysis with an appropriate treatment comparator; external replication of the interaction	“Predicts benefit from therapy” inferred from prognostic association or from a treated-only cohort

Second, public data support molecular subtyping. TCGA and Asian Cancer Research Group classifications remain central references, and many secondary analyses reinterpret subtypes through immune, stromal, metabolic, epigenetic, or single-cell lenses (2, 3, 5, 64). Subtyping is useful when it clarifies heterogeneity, prognosis, or treatment relevance. It is weaker when labels are assigned inconsistently or when a new subtype does not add information beyond established classifications.

Third, prognosis and risk stratification are highly productive but require restraint. Risk models can prioritize aggressive disease biology and identify pathways for study. They become more relevant to clinical translation when they outperform clinical variables, remain calibrated in external cohorts, and show decision-relevant net benefit. Without these features, a statistically significant score is a retrospective association.

Fourth, public omics can generate therapy-response hypotheses. Studies infer chemotherapy sensitivity, immune-checkpoint relevance, immunotherapy response, antiangiogenic combinations, and drug-resistance mechanisms from transcriptomic, immune, and single-cell data (65–70). These analyses are useful when clearly framed as hypothesis generation. They should not equate checkpoint expression, deconvolution, or cell-line drug-sensitivity scores with clinical response unless treatment-exposed patient cohorts or experimental models support the claim.

Fifth, public omics has deepened tumor-microenvironment interpretation. Bulk deconvolution, single-cell transcriptomics, and spatial methods collectively show that gastric cancer progression and therapy response are shaped by malignant epithelial states, fibroblasts, macrophages, neutrophils, T cells, microbial factors, and tissue architecture (25, 26, 29, 71). The opportunity is to move from isolated immune markers toward integrated TME models; the risk is overinterpreting inferred cells, predicted ligand-receptor pairs, or computational niches as directly observed mechanisms.

## 6 Methodological and translational limitations

The first limitation is cohort heterogeneity. Public datasets differ in inclusion criteria, histologic composition, tumor stage, anatomic site, geography, tissue processing, sequencing platform, normalization, treatment exposure, endpoint definition, and follow-up. These differences may reflect real biology, but they can also introduce confounding and batch effects. Analysts should avoid mechanically merging datasets and should report cohort-specific sensitivity analyses.

The second limitation is data leakage and overfitting. Public-omics datasets often include many candidate variables and relatively few events. The entry points for leakage — feature selection, cutoff optimization, normalization, batch correction, and hyperparameter tuning that uses information from validation data — and the four obligations that close them are set out in Section 4.2 and summarized in (**Box 2**); they are not repeated here. Overfitting is amplified when multiple themes and modeling approaches are tried until a favorable result is found. The solution is not to abandon public data; it is to separate discovery, internal validation, and external validation through a locked analysis plan.

The third limitation is weak clinical utility. External validation should mean applying a fixed model to an independent cohort, not rebuilding the model in another dataset. Calibration and decision-curve analysis should complement discrimination metrics (51–53), and gastric cancer models should be compared with TNM stage, histology, treatment variables, and molecular subtypes when available; the appraisal questions that operationalize these requirements are given in Sections 4.3 and 4.4 and in **Table 4**. Without incremental value over clinical predictors, molecular signatures are unlikely to change practice.

**Table 4:** GC-PROVE practical appraisal guide for gastric cancer public-omics secondary analysis†.

GC-PROVE domain	Low	Partial	Preferred	Reviewer question
Problem anchoring	Pipeline- or gene-set-driven study without a clear question	Broad biological theme but vague clinical or mechanistic target	Explicit clinical, biological, or validation question with intended claim type	What decision, mechanism, or validation gap does the analysis address?
Resource provenance	Dataset names missing or unreconstructable	Accessions reported but filtering, endpoints, or overlap incompletely described	Accessions, versions, sample filters, endpoint definitions, missingness, and cohort overlap are auditable	Can another analyst reconstruct the cohort?
Omics workflow transparency	Preprocessing, feature selection, or model formula unavailable	Partial workflow reported, but code, versions, or tuning details incomplete	Normalization, gene mapping, batch correction, feature selection, model formula, coefficients, software versions, and computational environment are documented	Are preprocessing and modeling choices reproducible?
Validation hierarchy	Discovery cohort only or validation after model rebuilding	External or biological support present but model/thresholds are not fully locked	Locked external validation plus relevant tissue, protein, spatial, functional, or clinical support proportional to the claim	Is the claim appropriately limited by its validation level?
Evidence restraint	Conclusions imply clinical readiness from retrospective public data	Limitations mentioned but translational language remains stronger than evidence	Findings are framed as candidates or hypotheses unless clinical utility has been directly tested	Is clinical language matched to the evidence?
Prediction-model rigor	Discrimination or Kaplan-Meier separation only	Some calibration, decision-curve analysis, or clinical comparator is reported	Locked formula, calibration, decision-curve analysis, clinical comparator, and intended-use context are all reported	Does the model improve on clinical predictors in an interpretable context?
High-resolution omics rigor	Single-cell or spatial data used illustratively rather than inferentially	Cell annotation or spatial context discussed with limited validation	Integration choices, annotation uncertainty, spatial platform limits, and orthogonal support are explicitly addressed	Are cell states and spatial niches independently supported?
Reproducibility	No code, coefficients, cutoffs, package versions, or random seed	Partial code or parameters available	Executable code, model object, feature list, coefficients, cutoffs, versions, seeds, and derived materials are available	Could the central figure or risk score be reproduced?

The fourth limitation is biological interpretability. Differential expression does not prove driver status. WGCNA modules do not prove regulation. ceRNA networks do not prove RNA competition. Immune deconvolution does not prove cell function. Cell-cell communication inference does not prove signaling. Here, illustrative use means using single-cell or spatial data to echo a bulk result visually without source attribution, tissue-level validation, or orthogonal corroboration. Biological interpretation becomes stronger when multiple layers converge: a transcriptomic signal is localized to a cell type, spatially supported in tissue, associated with protein abundance or immunohistochemistry, and functionally tested.

The fifth limitation is reproducibility. Many studies do not provide complete code, package versions, preprocessing choices, model objects, coefficient formulas, cutoff definitions, or intermediate data. The minimum reproducible output is listed once, in (**Box 2**), and is not repeated here: executable code, data provenance, accession numbers, sample filters, model formulas, feature lists, random seeds when applicable, and software versions. Reproducibility determines whether a computational finding can be audited and extended.

The sixth limitation is redundancy and publication bias. Public-database studies often apply similar pipelines to theme-defined gene sets. Positive models are easier to publish than negative replication attempts. This creates discovery abundance without cumulative knowledge. A study makes a stronger contribution when it changes biological understanding, improves methodology, provides stronger validation, or clarifies translational relevance. Finally, this review is narrative and interpretive rather than quantitative; its purpose is to stimulate methodological discussion, not to provide formal quality ratings of individual studies.

## 7 Future directions: from discovery abundance to GC-PROVE

Future gastric cancer public-omics studies should begin with clinically or biologically anchored questions. Examples include whether molecular data improve prognosis beyond TNM stage, whether recurrent TME programs predict immunotherapy response in treated cohorts, whether epigenomic markers support early detection, or whether spatial neighborhoods explain resistance. Starting with a defined question reduces the temptation to build signatures around fashionable gene sets.

GC-PROVE was developed as an author-proposed heuristic by synthesizing recurring failure modes observed in gastric cancer public-omics studies with established principles from prediction-model reporting and appraisal (TRIPOD, TRIPOD+AI, PROBAST, PROBAST+AI), tumor-marker prognostic reporting (REMARK), reproducible computational research, biomarker validation hierarchies, and high-dimensional omics interpretation. It is not intended to replace these standards. Reporting completeness (what should be described) is distinct from methodological quality and risk of bias (how trustworthy a model is); GC-PROVE addresses the former for gastric cancer public-omics while deferring to PROBAST-type tools for the latter. **Table 5** sets out which GC-PROVE elements are already covered by existing standards (48–50, 72, 73) and, more importantly, what GC-PROVE adds beyond a gastric-cancer-specific restatement of that guidance.

**Table 5:** Relationship of GC-PROVE to existing reporting and risk-of-bias standards.

GC-PROVE element	Already covered by existing standards (48–50, 72, 73)	What GC-PROVE adds beyond a domain-specific restatement
Problem anchoring	TRIPOD and PROBAST both require a clearly defined intended use and outcome; REMARK requires an explicitly stated hypothesis and a pre-specified marker	Appraisal starts before a model exists, so the requirement also applies to secondary analyses that never produce a prediction model (differential-expression, ceRNA, WGCNA, deconvolution, or cell-cell-communication studies), which TRIPOD, PROBAST, and REMARK do not cover; public-data reuse is explicitly classified as hypothesis generation or as a clinical claim.
Resource provenance	TRIPOD+AI requires data-source and participant-flow reporting	Adds cross-database cohort-overlap and duplicate-sample checks between TCGA-STAD and public GEO cohorts, plus reconstructable sample filters; TRIPOD+AI covers data-source reporting for one study but not the reuse of the same patients across public repositories.
Omics workflow transparency	TRIPOD+AI covers model specification and hyperparameters; REMARK covers assay method, specimen handling, and marker measurement	Adds omics-specific items absent from all four standards: gene mapping, the method and placement of batch correction, and whether feature selection occurred inside the locked training workflow.
Validation hierarchy	TRIPOD and PROBAST define development, internal, and external validation; REMARK specifies the validation of tumor marker prognostic studies	Adds an explicit ordering across evidence types that no single standard spans, from public discovery through external replication, tissue or protein support, and functional perturbation to clinical utility, so that the validation level can be matched to the claim.
Evidence restraint	PROBAST addresses applicability and bias in prediction studies	Extends applicability assessment from prediction models to non-model omics claims, including ceRNA axes, deconvolution estimates, ligand-receptor inference, and foundation-model embeddings, for which PROBAST and REMARK provide no items.
Prediction-model rigor	Fully covered by TRIPOD, TRIPOD+AI, PROBAST, PROBAST+AI	No new requirement. GC-PROVE defers entirely to TRIPOD+AI and PROBAST here and functions only as a triage prompt to apply them in full.
High-resolution omics rigor	Partly covered by reporting guidance for single-cell studies	Adds cautions outside the scope of all four standards: pseudoreplication of cells treated as independent samples, ambient RNA, doublets, and spatial region-of-interest selection bias.
Reproducibility	FAIR principles and journal code-availability policies; REMARK requires reporting of the marker assay and analysis sufficient for replication	Converts general FAIR and code-availability expectations into a concrete minimum artifact set for omics risk scores (model object, feature list, coefficients, cutoffs, versions, seeds) that can be checked directly during review.

Four features distinguish GC-PROVE from a gastric-cancer-specific restatement of TRIPOD, TRIPOD+AI, PROBAST, or REMARK. First, the unit of appraisal differs. TRIPOD, TRIPOD+AI, and PROBAST appraise a single prediction-model study once a model has been developed, and REMARK appraises a single tumor-marker prognostic study. GC-PROVE appraises the whole public-data reuse chain of a secondary analysis, beginning with problem anchoring and resource provenance before any model exists, so it can also be applied to studies that never produce a prediction model, including differential-expression, ceRNA, WGCNA, deconvolution, and cell-cell-communication analyses. Second, the range of claim types differs. TRIPOD and PROBAST are built for prediction models and REMARK for tumor markers; none provides items for co-expression modules, ceRNA axes, ligand-receptor inference, deconvolution estimates, or foundation-model embeddings, although all are common claim types in gastric cancer public-omics (**Table 3**). Third, the output differs. TRIPOD and TRIPOD+AI record whether an item was reported and PROBAST records a risk-of-bias judgment for a prediction model; GC-PROVE additionally requires the claim to be matched to the evidence actually available (**Table 3**, **4**), so that a study with locked external validation still cannot claim clinical utility without calibration, a clinical comparator, and decision-curve analysis. Fourth, the intended function differs. GC-PROVE compresses the appraisal into five domains that can be applied at editorial triage to decide whether a full TRIPOD+AI or PROBAST assessment is warranted, supported by a downloadable per-domain checklist; it is therefore a screening and routing layer that points to the appropriate full standard rather than a competing instrument. These claims are deliberately limited. GC-PROVE is an author-proposed heuristic with no validation study, no inter-rater reliability estimates, and no demonstrated effect on reporting quality; until such evidence exists it should be read as a structured prompt for authors, reviewers, and editors rather than as a validated measurement tool.

Problem anchoring asks what decision or mechanism the analysis addresses. Resource provenance requires accessions, sample filters, endpoint definitions, cohort overlap checks, and preprocessing details. Omics workflow transparency requires documented normalization, feature selection, batch correction, model tuning, and sensitivity analyses. Validation hierarchy distinguishes public discovery from external replication, tissue/protein support, functional validation, and clinical utility. Evidence restraint requires authors to label computational findings as candidates unless stronger evidence exists.

The framework is shown in **Figure 4**. GC-PROVE is a mnemonic, not a validated scoring instrument. Its five core domains (the first five rows of **Table 4**) address problem anchoring, resource provenance, workflow transparency, validation hierarchy, and evidence restraint; the final three rows extend the appraisal to prediction-model rigor, high-resolution omics quality, and reproducibility. The framework is intended to organize reporting and review questions and does not replace established standards such as TRIPOD, TRIPOD+AI, PROBAST, or formal risk-of-bias tools. A downloadable GC-PROVE appraisal checklist is provided as a supplementary aid for authors and reviewers.

**Figure 4:**
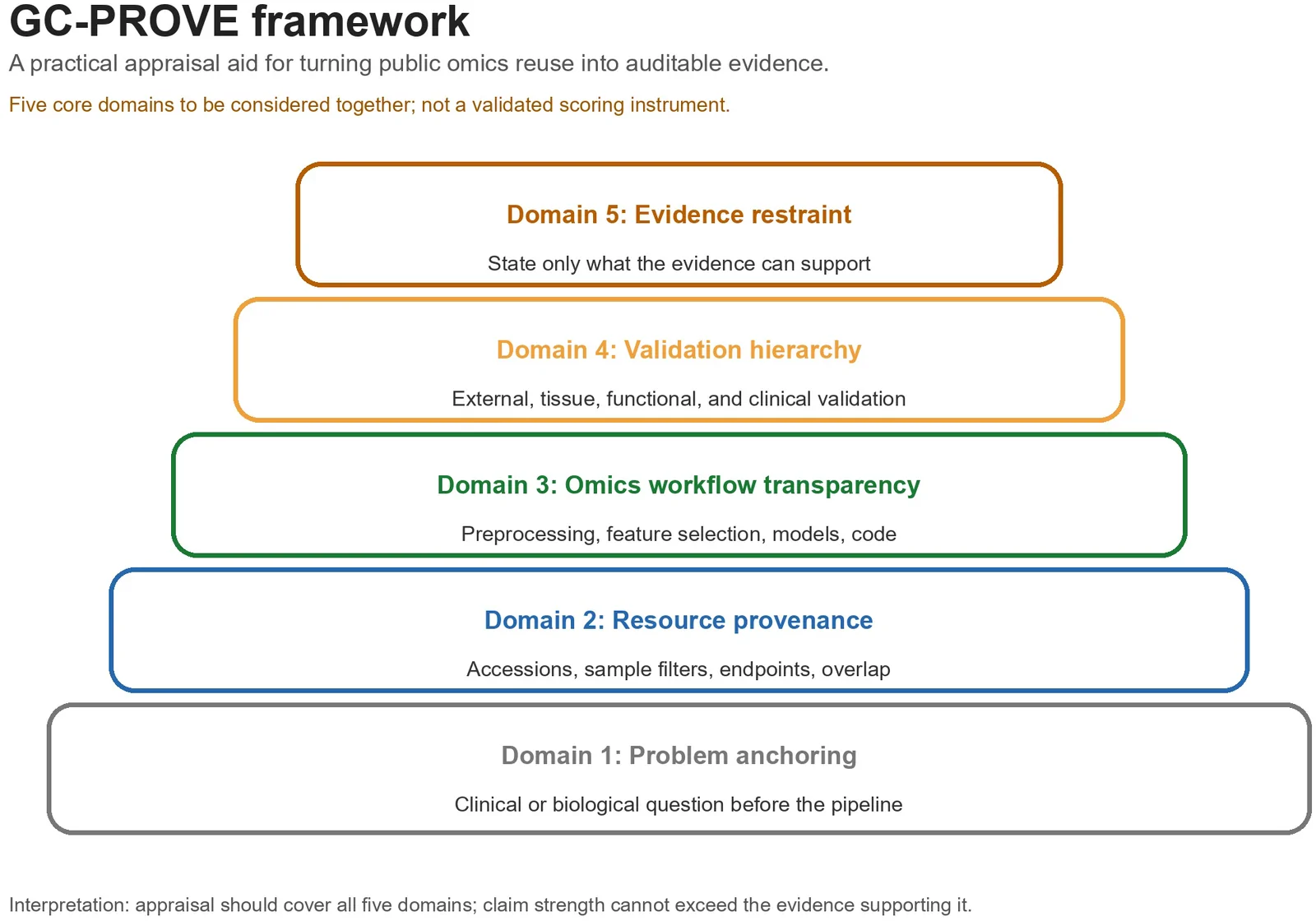
GC-PROVE framework. GC-PROVE is presented as five core domains: problem anchoring, resource provenance, omics workflow transparency, validation hierarchy, and evidence restraint. The figure labels these as Domain 1–5, not Level 1–5. The five domains are intended to be considered together; GC-PROVE is an author-proposed mnemonic, not a validated scoring instrument.

Hypothesis prioritization is itself a legitimate endpoint when it is stated explicitly and kept within the evidence available.

Five published studies illustrate how GC-PROVE produces traceable judgments. They were selected because their validation designs, claim types, and reporting completeness differ, and each is documented against the full text of the source article in **Supplementary Table S5**. The first study (Chang 2023, reference 37) anchors a mitochondrial-gene risk score to a stated biological question, reports a locked LASSO-Cox formula, applies it to two independent external cohorts (GSE66229 and GSE15459), and reports calibration and decision-curve analysis; under GC-PROVE it scores favorably on resource provenance, workflow transparency, validation hierarchy, and prediction-model rigor. Its main residual limitation is that the model is not compared against a molecular subtype or a full clinical model, which limits the claim to a candidate prognostic stratification rather than demonstrated incremental utility. The second study (Jiang 2025, reference 42) also defines a clear question and reports calibration and decision-curve analysis, but its validation is a random training/testing split of the same TCGA cohort rather than an independent cohort. Under GC-PROVE this design places the work at the discovery end of the validation hierarchy, and the appropriate claim is hypothesis prioritization rather than external validation. A third study (Li 2024, reference 40) derives a pyroptosis-related signature in TCGA-STAD, validates it in the independent Asian Cancer Research Group cohort (GSE62254), and reports calibration, but uses single-cell data for contextualization rather than as an independent test of the cellular claim; resource provenance and validation hierarchy are favorable, high-resolution omics rigor is partial because the single-cell component localizes the signal without validating it, and prediction-model rigor is partial because calibration is reported without decision-curve analysis.

A fourth study (Cai 2024, reference 41) addresses a clinically specific endpoint, peritoneal recurrence, and validates in a separate patient set from the same institution with additional interactive analysis against TCGA, but reports no calibration or decision-curve analysis and uses a small paired-tissue discovery set. Problem anchoring and resource provenance are favorable, whereas validation hierarchy is partial because the validation set is not an independent multicenter cohort; the defensible claim is therefore a candidate marker for peritoneal-recurrence risk requiring external multicenter validation. This case also shows where REMARK, rather than TRIPOD, is the governing reporting standard and where GC-PROVE contributes only the public-data reuse and claim-matching layer. A fifth study (Wang 2025, reference 47) integrates TCGA-STAD, CPTAC, and several single-cell resources in a deep-learning prognostic framework but reports only an internal held-out validation set, with no calibration, decision-curve analysis, or clinical comparator. Resource provenance is favorable, but the study sits at the discovery end of the validation hierarchy and prediction-model rigor and reproducibility are the binding constraints; this is precisely the profile for which GC-PROVE should trigger, rather than replace, a full TRIPOD+AI and PROBAST assessment. The five entries differ only in their documented design features, which shows that GC-PROVE judgments can be traced to specific reported evidence rather than to an overall impression, and that a favorable judgment on one domain does not compensate for a weak one on another. As noted in Section 2, these appraisals are per-study judgments based on full-text reading and are categorically different from the aggregate database-scan signals.

Single-cell, spatial, foundation-model, and perturbation-aware tools will likely reshape secondary analysis during 2026-2030. These tools should be used to answer defined biological or clinical questions, not to add complexity for its own sake. Single-cell data should identify the cellular origin of bulk signals. Spatial data should test anatomical plausibility and tissue-level interactions. Foundation models should be benchmarked against transparent baselines and used for tasks where they demonstrably improve integration, annotation, perturbation prediction, or generalization. Perturbation-aware omics is especially important because the field is moving from describing cell states to asking how those states might change after genetic, pharmacologic, or microenvironmental intervention. Accordingly, tools and datasets related to gene regulatory networks, Perturb-seq, CRISPR screens, and in silico perturbation should be treated as hypothesis-prioritization systems that require experimental confirmation. High-resolution analysis should not repeat the weaknesses of bulk studies at higher dimensionality. The evidence hierarchy and near-term roadmap are shown in **Figure 5**, and the corresponding appraisal rubric is listed in **Table 4**.

**Figure 5:**
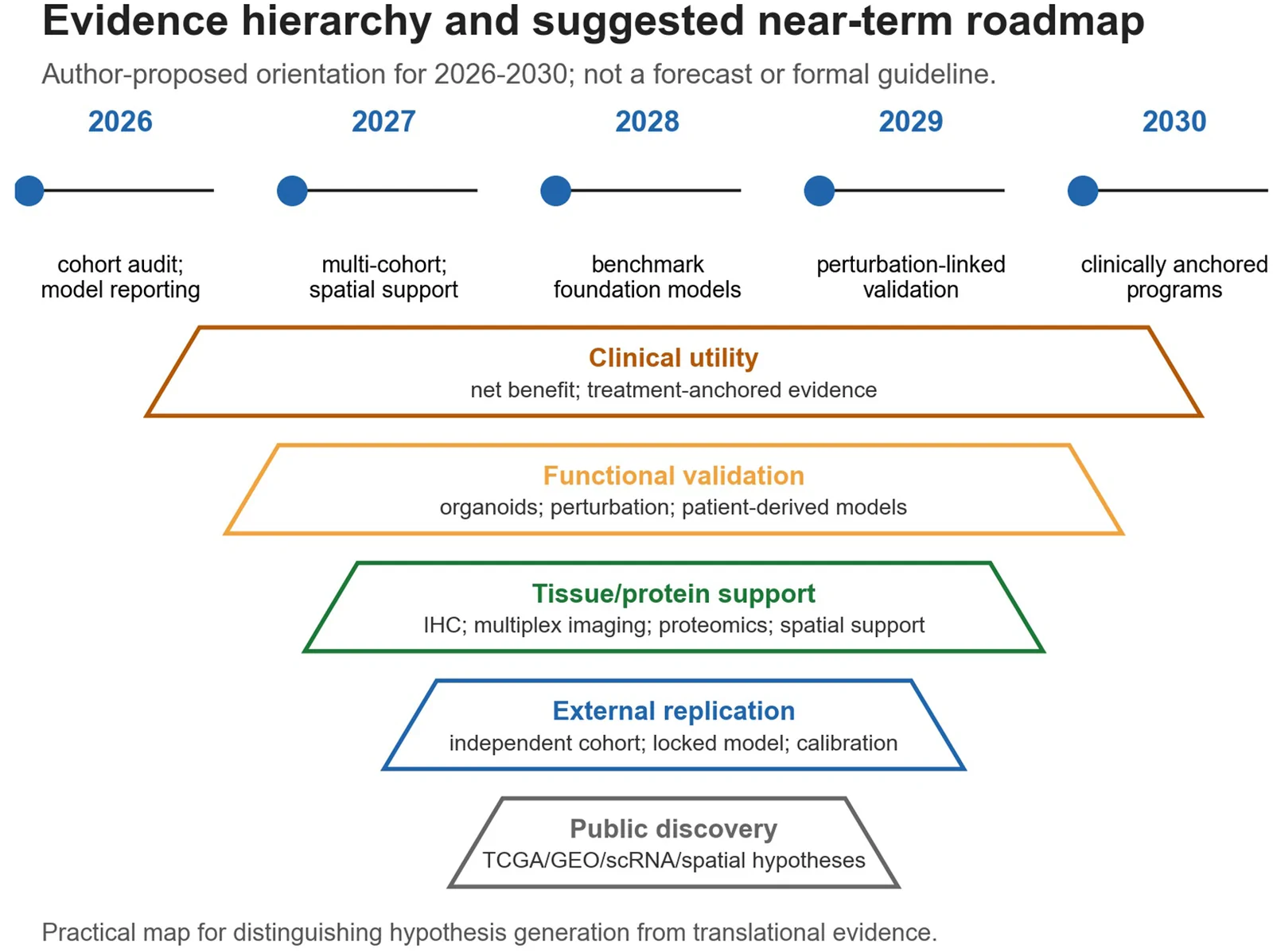
Evidence hierarchy and suggested near-term roadmap for public-omics secondary analysis. The hierarchy distinguishes public discovery from external replication, tissue/protein support, functional validation, and clinical utility. The roadmap is an author-proposed orientation for 2026-2030, emphasizing cohort auditing, multi-cohort and spatial triangulation, benchmarked foundation models, perturbation-linked validation, and clinically anchored research programs rather than a prediction of field development.

Finally, future work should connect computational hypotheses to experiments and clinical cohorts. qPCR, immunohistochemistry, multiplex imaging, organoids, perturbation screens, patient-derived models, and treatment-annotated cohorts can help determine whether a public-database signal reflects actionable biology. Future publications should give greater weight to locked external validation, failed replication, benchmark datasets, and well-documented negative results. Unsuccessful external validation or failure to reproduce a reported association is a legitimate result: it can prevent reuse of unstable signatures or redirect attention to better-supported biology. Public-omics secondary analyses are most useful when they make the next experiment, validation cohort, or clinical question more precise.

## 8 Implications for authors, reviewers, and editors

Authors should design secondary analyses as evidence-building studies rather than as template-driven analyses. A publishable public-omics study should define the biological or clinical problem before selecting gene sets, specify dataset accessions and sample filters, lock the modeling pipeline before validation, and state what type of evidence the analysis can and cannot provide. When the intended claim is prognostic, a fixed model formula, calibration, clinical comparator, and external validation should be treated as core outputs rather than optional additions.

For reviewers, GC-PROVE provides a concise appraisal tool. The key questions are whether the study asks a nontrivial question, whether the public cohorts are auditable and non-overlapping, whether feature selection and validation are separated, whether biological interpretation is supported by orthogonal evidence, and whether the conclusions remain within the evidence level. These questions distinguish studies that merely rename a familiar pipeline from studies that improve cumulative knowledge.

For editors, the framework can help manage the rapid proliferation of public-database signatures. Manuscripts may be more informative when they contribute a stronger validation design, a clearer biological mechanism, a reusable analytical resource, a clinically anchored question, or a transparent benchmark. Conversely, a new risk score derived from familiar cohorts may not represent high-value evidence solely because it reports significant survival curves or favorable AUCs.

## 9 Conclusion

Public omics reuse now supports hypothesis generation across biomarker discovery, molecular subtyping, prognosis, therapy-response inference, and tumor-microenvironment mapping in gastric cancer. Progress should be judged by the credibility and reproducibility of the evidence chain, not by the number of additional signatures. Prognostic modeling illustrates the broader challenge: public data can generate clinically appealing models, but their value depends on avoiding leakage, demonstrating transportability, showing incremental utility, and resisting overinterpretation. The goal is auditable, biologically triangulated, and clinically anchored translational evidence. GC-PROVE can be used alongside established reporting and validation standards to evaluate public-omics secondary analyses and to strengthen the chain from computational discovery to translational relevance.
