# Supplementary material for: Public omics data reuse in gastric cancer: from discovery abundance to translational evidence
Source: Front Oncol. 2026 Oct 26;16:1974260. doi: 10.3389/fonc.2026.1974260 (PMC13746168; doi:10.3389/fonc.2026.1974260)
Supplement: Supplementary file 1 [file DataSheet1.zip › supplementary/Supplementary_Figure_S1_publication_trends.pdf]

Publication trends in gastric cancer public-omics topics

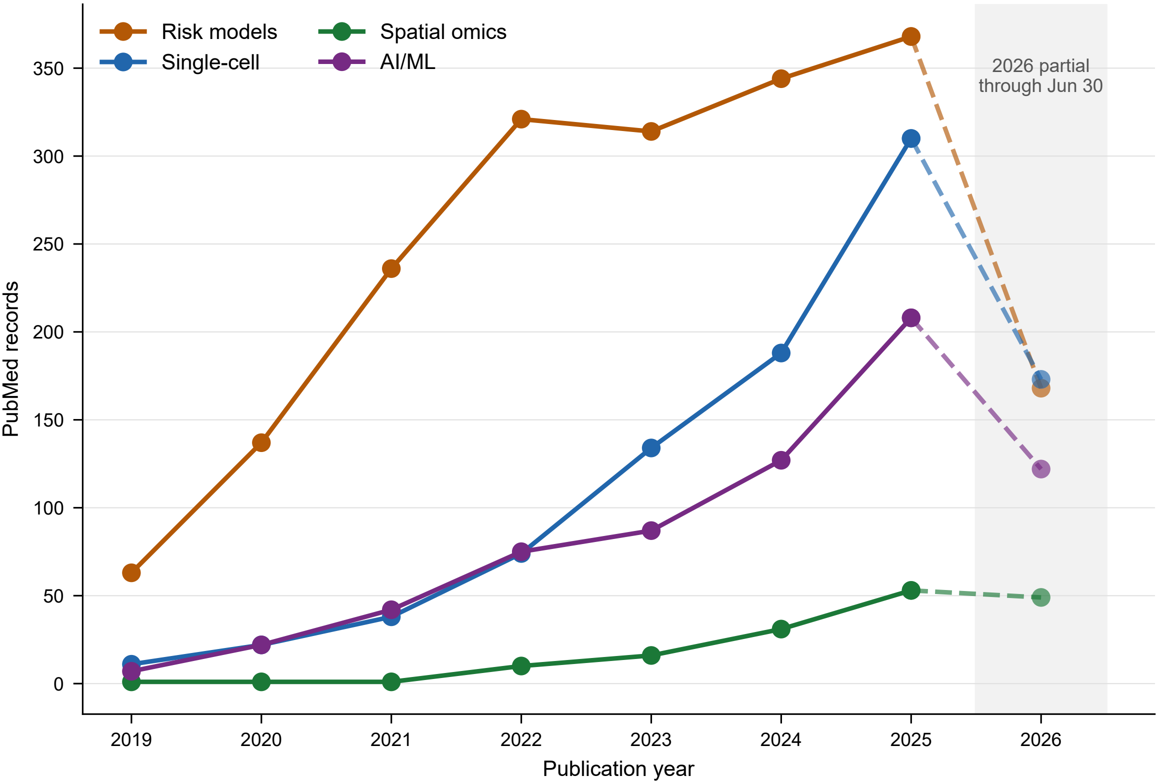

Counts are query-dependent supportive indicators; 2026 is not a complete publication year.
