# Supplementary material for: Public omics data reuse in gastric cancer: from discovery abundance to translational evidence
Source: Front Oncol. 2026 Oct 26;16:1974260. doi: 10.3389/fonc.2026.1974260 (PMC13746168; doi:10.3389/fonc.2026.1974260)
Supplement: Supplementary file 1 [file DataSheet1.zip › supplementary/Supplementary_File_S1_pubmed_search_log.docx]

# Supplementary File S1. PubMed Search Log

Search strings and PubMed QueryTranslation outputs used for the contextual database scans supporting the narrative review. Evidence window: 2026-06-30.

- Search date: 2026-06-30

- Database: PubMed via NCBI E-utilities.

- Date range: 2019-01-01 to 2026-06-30.

- Role: supportive evidence scan for a narrative review; not systematic-review screening.

# Public omics secondary analysis in gastric cancer

**Query**

```text

(("gastric cancer"[Title/Abstract] OR "stomach adenocarcinoma"[Title/Abstract] OR "stomach cancer"[Title/Abstract]) AND (TCGA[Title/Abstract] OR GEO[Title/Abstract] OR "public database"[Title/Abstract] OR "public datasets"[Title/Abstract] OR bioinformatics[Title/Abstract] OR "secondary analysis"[Title/Abstract] OR transcriptome[Title/Abstract]) NOT retracted publication[Publication Type]) AND ("2019/01/01"[Date - Publication] : "2026/06/30"[Date - Publication])

```

**QueryTranslation**

```text

((("gastric cancer"[Title/Abstract] OR "stomach adenocarcinoma"[Title/Abstract] OR "stomach cancer"[Title/Abstract]) AND ("TCGA"[Title/Abstract] OR "GEO"[Title/Abstract] OR "public database"[Title/Abstract] OR "public datasets"[Title/Abstract] OR "bioinformatics"[Title/Abstract] OR "secondary analysis"[Title/Abstract] OR "transcriptome"[Title/Abstract])) NOT "retracted publication"[Publication Type]) AND 2019/01/01:2026/06/30[Date - Publication]

```

# Prognostic signatures and risk models

**Query**

```text

(("gastric cancer"[Title/Abstract] OR "stomach adenocarcinoma"[Title/Abstract]) AND ("risk signature"[Title/Abstract] OR "prognostic signature"[Title/Abstract] OR "prognostic model"[Title/Abstract] OR nomogram[Title/Abstract] OR "survival model"[Title/Abstract]) NOT retracted publication[Publication Type]) AND ("2019/01/01"[Date - Publication] : "2026/06/30"[Date - Publication])

```

**QueryTranslation**

```text

((("gastric cancer"[Title/Abstract] OR "stomach adenocarcinoma"[Title/Abstract]) AND ("risk signature"[Title/Abstract] OR "prognostic signature"[Title/Abstract] OR "prognostic model"[Title/Abstract] OR "nomogram"[Title/Abstract] OR "survival model"[Title/Abstract])) NOT "retracted publication"[Publication Type]) AND 2019/01/01:2026/06/30[Date - Publication]

```

# External validation language in prognostic modeling

**Query**

```text

(("gastric cancer"[Title/Abstract] OR "stomach adenocarcinoma"[Title/Abstract]) AND ("risk signature"[Title/Abstract] OR "prognostic signature"[Title/Abstract] OR "prognostic model"[Title/Abstract] OR nomogram[Title/Abstract]) AND ("external validation"[Title/Abstract] OR "validation cohort"[Title/Abstract] OR "independent cohort"[Title/Abstract] OR GEO[Title/Abstract]) NOT retracted publication[Publication Type]) AND ("2019/01/01"[Date - Publication] : "2026/06/30"[Date - Publication])

```

**QueryTranslation**

```text

((("gastric cancer"[Title/Abstract] OR "stomach adenocarcinoma"[Title/Abstract]) AND ("risk signature"[Title/Abstract] OR "prognostic signature"[Title/Abstract] OR "prognostic model"[Title/Abstract] OR "nomogram"[Title/Abstract]) AND ("external validation"[Title/Abstract] OR "validation cohort"[Title/Abstract] OR "independent cohort"[Title/Abstract] OR "GEO"[Title/Abstract])) NOT "retracted publication"[Publication Type]) AND 2019/01/01:2026/06/30[Date - Publication]

```

# Single-cell gastric cancer omics

**Query**

```text

(("gastric cancer"[Title/Abstract] OR "stomach adenocarcinoma"[Title/Abstract]) AND ("single-cell"[Title/Abstract] OR scRNA[Title/Abstract] OR "single cell"[Title/Abstract]) NOT retracted publication[Publication Type]) AND ("2019/01/01"[Date - Publication] : "2026/06/30"[Date - Publication])

```

**QueryTranslation**

```text

((("gastric cancer"[Title/Abstract] OR "stomach adenocarcinoma"[Title/Abstract]) AND ("single-cell"[Title/Abstract] OR "scRNA"[Title/Abstract] OR "single-cell"[Title/Abstract])) NOT "retracted publication"[Publication Type]) AND 2019/01/01:2026/06/30[Date - Publication]

```

# Spatial omics in gastric cancer

**Query**

```text

(("gastric cancer"[Title/Abstract] OR "stomach adenocarcinoma"[Title/Abstract]) AND ("spatial transcriptomics"[Title/Abstract] OR "spatial omics"[Title/Abstract] OR "spatial multi-omics"[Title/Abstract] OR "multiplex immunofluorescence"[Title/Abstract] OR Xenium[Title/Abstract] OR Visium[Title/Abstract] OR CosMx[Title/Abstract] OR MERFISH[Title/Abstract] OR "Stereo-seq"[Title/Abstract]) NOT retracted publication[Publication Type]) AND ("2019/01/01"[Date - Publication] : "2026/06/30"[Date - Publication])

```

**QueryTranslation**

```text

((("gastric cancer"[Title/Abstract] OR "stomach adenocarcinoma"[Title/Abstract]) AND ("spatial transcriptomics"[Title/Abstract] OR "spatial omics"[Title/Abstract] OR "spatial multi-omics"[Title/Abstract] OR "multiplex immunofluorescence"[Title/Abstract] OR "Xenium"[Title/Abstract] OR "Visium"[Title/Abstract] OR "CosMx"[Title/Abstract] OR "MERFISH"[Title/Abstract] OR "Stereo-seq"[Title/Abstract])) NOT "retracted publication"[Publication Type]) AND 2019/01/01:2026/06/30[Date - Publication]

```

# AI and machine learning public-omics prognosis

**Query**

```text

(("gastric cancer"[Title/Abstract] OR "stomach adenocarcinoma"[Title/Abstract]) AND ("machine learning"[Title/Abstract] OR "artificial intelligence"[Title/Abstract] OR "deep learning"[Title/Abstract] OR XGBoost[Title/Abstract] OR transformer[Title/Abstract]) AND (prognosis[Title/Abstract] OR survival[Title/Abstract] OR "risk model"[Title/Abstract] OR "public database"[Title/Abstract] OR transcriptome[Title/Abstract] OR TCGA[Title/Abstract] OR GEO[Title/Abstract]) NOT retracted publication[Publication Type]) AND ("2019/01/01"[Date - Publication] : "2026/06/30"[Date - Publication])

```

**QueryTranslation**

```text

((("gastric cancer"[Title/Abstract] OR "stomach adenocarcinoma"[Title/Abstract]) AND ("machine learning"[Title/Abstract] OR "artificial intelligence"[Title/Abstract] OR "deep learning"[Title/Abstract] OR "XGBoost"[Title/Abstract] OR "transformer"[Title/Abstract]) AND ("prognosis"[Title/Abstract] OR "survival"[Title/Abstract] OR "risk model"[Title/Abstract] OR "public database"[Title/Abstract] OR "transcriptome"[Title/Abstract] OR "TCGA"[Title/Abstract] OR "GEO"[Title/Abstract])) NOT "retracted publication"[Publication Type]) AND 2019/01/01:2026/06/30[Date - Publication]

```

# Reproducibility and reporting in omics/bioinformatics

**Query**

```text

(("reproducible research"[Title/Abstract] OR reproducibility[Title/Abstract] OR "code availability"[Title/Abstract] OR "data leakage"[Title/Abstract] OR "batch effect"[Title/Abstract] OR "external validation"[Title/Abstract] OR calibration[Title/Abstract]) AND (omics[Title/Abstract] OR genomics[Title/Abstract] OR transcriptomics[Title/Abstract] OR bioinformatics[Title/Abstract] OR "prediction model"[Title/Abstract]) NOT retracted publication[Publication Type]) AND ("2019/01/01"[Date - Publication] : "2026/06/30"[Date - Publication])

```

**QueryTranslation**

```text

((("reproducible research"[Title/Abstract] OR "reproducibility"[Title/Abstract] OR "code availability"[Title/Abstract] OR "data leakage"[Title/Abstract] OR "batch effect"[Title/Abstract] OR "external validation"[Title/Abstract] OR "calibration"[Title/Abstract]) AND ("omics"[Title/Abstract] OR "genomics"[Title/Abstract] OR "transcriptomics"[Title/Abstract] OR "bioinformatics"[Title/Abstract] OR "prediction model"[Title/Abstract])) NOT "retracted publication"[Publication Type]) AND 2019/01/01:2026/06/30[Date - Publication]

```

# Interpretation of the 100-record screen

This title/abstract-level scan was illustrative. It was not dual-screened or used for formal quality appraisal or field-wide prevalence estimation. The screened records and predefined text-mining rules are provided for reproducibility.
