# Supplementary material for: Public omics data reuse in gastric cancer: from discovery abundance to translational evidence
Source: Front Oncol. 2026 Oct 26;16:1974260. doi: 10.3389/fonc.2026.1974260 (PMC13746168; doi:10.3389/fonc.2026.1974260)
Supplement: Supplementary file 1 [file DataSheet1.zip › supplementary/Supplementary_File_S2_record_set_documentation.docx]

# Supplementary File S2. Record-set documentation and year-assignment rule

Companion to Supplementary Table S2 (publication trends) and Supplementary File S2

(PMID-level record sets).

## 1. What is provided

| **File** | **Content** |
| --- | --- |
| `Supplementary_File_S2_pmid_list_public_omics.csv` | Complete PMID list for the public-omics secondary-analysis topic query |
| `Supplementary_File_S2_pmid_list_prognostic.csv` | Complete PMID list for the prognostic-signature/risk-model topic query |
| `Supplementary_File_S2_pmid_list_single_cell.csv` | Complete PMID list for the single-cell topic query |
| `Supplementary_File_S2_pmid_list_spatial.csv` | Complete PMID list for the spatial-omics topic query |
| `Supplementary_File_S2_pmid_list_ai_ml.csv` | Complete PMID list for the AI/machine-learning topic query |
| `Supplementary_File_S2_record_set_summary.csv` | Record counts and PMID-list sizes per topic |
| `Supplementary_Table_S2_publication_trends.csv` | Yearly counts per topic |

The topic queries are given in full, together with their PubMed QueryTranslation outputs, in

Supplementary File S1.

## 2. Year-assignment rule (why yearly sums exceed the date-bounded total)

Each record was assigned a publication year using the following rule, applied in order:

1. If PubMed reports a print publication date (`PubStatus = ppublish`), that year is used.
2. If no print year is available, the year of the journal issue (`<PubDate>`) is used.
3. If neither is present, the electronic publication year (`PubStatus = epublish`) is used.

A record can therefore carry an electronic publication year and a print publication year that fall

in **different calendar years**. When the yearly series is built, such a record is counted in both

years, while the date-bounded topic query counts it exactly once. This is why the yearly values in

Supplementary Table S2 sum to more than the corresponding date-bounded total:

| **Topic** | **Sum of yearly values** | **Date-bounded total** | **Difference** |
| --- | --- | --- | --- |
| Prognostic signatures and risk models | 1,951 | 1,812 | +139 (7.7%) |
| Single-cell gastric cancer omics | 950 | 876 | +74 (8.4%) |
| Spatial omics in gastric cancer | 162 | 148 | +14 (9.5%) |
| AI and machine-learning public-omics prognosis | 690 | 636 | +54 (8.5%) |

To test this explanation rather than assert it, the publication-date fields were retrieved for

every record in the prognostic topic record set and the two years were compared directly:

| **Measure** | **Value** |
| --- | --- |
| Records parsed | 1,839 |
| Records with an electronic publication date (ArticleDate) | 1,592 |
| Records with a print/issue date (PubDate) | 1,839 |
| **Records whose electronic and print years differ** | **147 (8.0%)** |
| Yearly sum implied by dual counting (1,839 + 147) | 1,986 |
| Observed excess at the original search date (1,951 - 1,812) | 139 (7.7%) |

The measured proportion of disagreements between the two year fields (8.0%) matches the observed

excess in the yearly series (7.7%) almost exactly, and the same order of excess appears in every

other topic (8.4% single-cell, 9.5% spatial, 8.5% AI or machine-learning). This confirms that the

yearly sums exceed the date-bounded total because of dual year assignment, not because the queries

differ in scope. The date-bounded total is the figure quoted in the main text, because it counts

each record once.

## 3. Indexing lag within a fixed date window

The date window (2019-01-01 to 2026-06-30) is fixed, but PubMed continues to add records whose

publication date falls inside it. Re-running the identical queries at the revision date therefore

retrieves slightly more records than at the original search date:

| **Topic** | **At first submission** | **At revision re-run** |
| --- | --- | --- |
| Public omics secondary analysis in gastric cancer | 4,416 | 4,460 |
| Prognostic signatures and risk models | 1,812 | 1,840 |
| Single-cell gastric cancer omics | 876 | 904 |
| Spatial omics in gastric cancer | 148 | 159 |
| AI and machine-learning public-omics prognosis | 636 | 662 |

The counts reported in the manuscript are those captured at the original search date. The

PMID-level lists provided here are from the revision re-run and are complete for each topic. Both

sets are query-dependent indicators of literature volume, not systematic prevalence estimates.

## 4. Reproducing the record sets

Run each query from Supplementary File S1 against the PubMed E-utilities `esearch` endpoint with

`usehistory=y` and `retmax` set to the full count, then retrieve the PMID list. A script that

performs exactly this is provided in the deposited reproducibility package as

`fetch_pmid_sets.py`.
