# Supplementary material for: Public omics data reuse in gastric cancer: from discovery abundance to translational evidence
Source: Front Oncol. 2026 Oct 26;16:1974260. doi: 10.3389/fonc.2026.1974260 (PMC13746168; doi:10.3389/fonc.2026.1974260)
Supplement: Supplementary file 1 [file DataSheet1.zip › supplementary/Supplementary_File_S3_coding_rules.docx]

# Supplementary File S3. Coding rules and extraction procedure for the reporting-signal screen

Companion to Supplementary Table S3 (signature reporting signals).

## 1. What the screen is, and what it is not

The screen is a **title/abstract-level text signal screen** performed on the 100 most recent PubMed

records returned by the prognostic-signature/risk-model query (see Supplementary File S1), sorted by

date in descending order through 2026-06-30.

It is **not** a quality appraisal and **not** a measure of the prevalence of methodological

practice in gastric cancer public-omics research. The prognosis query deliberately does not require

public-data reuse or omics analysis, so the screened corpus is broad prognosis literature in gastric

cancer and includes clinical, imaging, and cost-effectiveness studies. The frequencies below

describe what is *mentioned in titles and abstracts* within that corpus.

## 2. Retrieval settings

| **Setting** | **Value** |
| --- | --- |
| Database | PubMed |
| Query | Prognostic signatures and risk models (Supplementary File S1) |
| Date filter | 2019-01-01 to 2026-06-30 (Date - Publication) |
| Sort order | Date, descending |
| Records retrieved | 100 most recent |
| Retrieval date | 2026-06-30 |
| Output | PMID, year, title, DOI, and the six binary signal fields |

The 100 records and their per-record coding are listed in Supplementary Table S3.

## 3. Coding rules

Each record was coded in a single pass by one author. Coding was performed on the **title and

abstract only**; full texts were not screened. A field was coded `True` when at least one of the

listed terms or their close variants appeared in the title or abstract, and `False` otherwise. No

inference was made from the absence of a term.

| **Field** | **Coded `True` when the title or abstract contains** |
| --- | --- |
| `mentions_external_validation` | "external validation", "externally validated", "validation cohort", "independent cohort", "independent validation", "replicated in" |
| `mentions_calibration` | "calibration", "calibrated", "calibration curve" |
| `mentions_decision_curve` | "decision curve", "decision-curve analysis", "net benefit", "DCA" |
| `mentions_clinical_comparator` | "compared with" / "versus" / "in addition to" used with clinical variables, TNM stage, clinicopathological features, or an established model |
| `mentions_experimental_validation` | "qPCR", "RT-qPCR", "western blot", "immunohistochemistry", "in vitro", "in vivo", "cell line", "functional assay" |
| `mentions_code_availability` | "code", "script", "GitHub", "repository", "available at", "software package" used in a data- or code-availability sense |

**Single coding.** Coding was performed once by one author without duplicate independent coding and

without a formal disagreement-resolution step. The screen is therefore reported as an illustrative

signal rather than as a reproducible prevalence estimate. The coding fields are provided

per-record so that any reader can recode the corpus independently.

## 4. Extraction procedure

1. Run the prognostic-signature query with the date filter and sort the result by date, descending.
2. Export the first 100 records with their PMID, year, title, and DOI.
3. Apply the coding rules in Section 3 to each title and abstract.
4. Record each field as `True` or `False`; record no partial values.
5. Tabulate field frequencies across the 100 records.

## 5. Interpretation of the two reported frequencies

The two figures that reviewers asked to have reconciled come from **different samples and different

coding definitions** and are not directly comparable.

**Supplementary Table S1, `external_validation_mentions` column.** This column is a *topic

cross-tabulation*. For each topic row, the 100 screened records were classified by topic, and the

column records how many of those records are themselves *about* external validation (that is, the

record is a validation study or its central subject is external validation). For the prognostic

topic row the value is 0, meaning that none of the 100 most recent prognostic records screened is

primarily an external-validation study.

**Supplementary Table S3, `mentions_external_validation` column.** This column is a *term-mention

flag* applied to the same prognostic corpus. It records how many of the 100 records use

external-validation language anywhere in the title or abstract, regardless of whether validation is

the topic of the paper. The value is 46.

A record can therefore mention external-validation language without being an external-validation

study, and vice versa. The two frequencies answer different questions — "is this paper about

external validation?" versus "does this paper talk about external validation?" — and both are

reported because they carry different information.

- **Code-availability mentions.** Zero of 100 records mentioned code availability in the title or

abstract. This is an **abstract-level lower bound only**. Code statements typically appear in

full-text data-availability declarations, so zero mentions in titles and abstracts does **not**

indicate that no code exists for these studies. The zero value should be read as an observation

about the screened text, not as evidence about underlying practice.
