# Supplementary material for: Public omics data reuse in gastric cancer: from discovery abundance to translational evidence
Source: Front Oncol. 2026 Oct 26;16:1974260. doi: 10.3389/fonc.2026.1974260 (PMC13746168; doi:10.3389/fonc.2026.1974260)
Supplement: Supplementary file 1 [file DataSheet1.zip › supplementary/Supplementary_GC_PROVE_Checklist.docx]

# Supplementary GC-PROVE Appraisal Checklist

This checklist is an appraisal aid for gastric cancer public-omics secondary analysis. It is not a formal risk-of-bias instrument and does not replace TRIPOD, TRIPOD+AI, PROBAST, or journal-specific reporting requirements.

Table 6. GC-PROVE appraisal rubric for gastric cancer public-omics secondary analysis

| **GC-PROVE domain** | **Score 0** | **Score 1** | **Score 2** | **Reviewer question** |
| --- | --- | --- | --- | --- |
| Problem anchoring | Pipeline- or gene-set-driven study without a clear question | Broad biological theme but vague clinical or mechanistic target | Explicit clinical, biological, or validation question with intended claim type | What decision, mechanism, or validation gap does the analysis address? |
| Resource provenance | Dataset names missing or unreconstructable | Accessions reported but filtering, endpoints, or overlap incompletely described | Accessions, versions, sample filters, endpoint definitions, missingness, and cohort overlap are auditable | Can another analyst reconstruct the cohort? |
| Omics workflow transparency | Preprocessing, feature selection, or model formula unavailable | Partial workflow reported, but code, versions, or tuning details incomplete | Normalization, gene mapping, batch correction, feature selection, model formula, coefficients, software versions, and code are provided | Are preprocessing and modeling choices reproducible? |
| Validation hierarchy | Discovery cohort only or validation after model rebuilding | External or biological support present but model/thresholds are not fully locked | Locked external validation plus relevant tissue, protein, spatial, functional, or clinical support proportional to the claim | Is the claim appropriately limited by its validation level? |
| Evidence restraint | Conclusions imply clinical readiness from retrospective public data | Limitations mentioned but translational language remains stronger than evidence | Findings are framed as candidates or hypotheses unless clinical utility has been directly tested | Is clinical language matched to the evidence? |
| Prediction-model rigor | Discrimination or Kaplan-Meier separation only | Some calibration, DCA, or clinical comparator is reported | Locked formula, calibration, DCA, clinical comparator, and intended-use context are all reported | Does the model improve on clinical predictors in an interpretable context? |
| High-resolution omics rigor | Single-cell or spatial data used as decorative confirmation | Cell annotation or spatial context discussed with limited validation | Integration choices, annotation uncertainty, spatial platform limits, and orthogonal support are explicitly addressed | Are cell states and spatial niches independently supported? |
| Reproducibility | No code, coefficients, cutoffs, package versions, or random seed | Partial code or parameters available | Executable code, model object, feature list, coefficients, cutoffs, versions, seeds, and derived materials are available | Could the central figure or risk score be reproduced? |
