# Supplementary material for: Public omics data reuse in gastric cancer: from discovery abundance to translational evidence
Source: Front Oncol. 2026 Oct 26;16:1974260. doi: 10.3389/fonc.2026.1974260 (PMC13746168; doi:10.3389/fonc.2026.1974260)
Supplement: Supplementary file 1 [file DataSheet1.zip › supplementary/Supplementary_GC_PROVE_Checklist.pdf]

Supplementary GC-PROVE Appraisal Checklist

This checklist is an appraisal aid for gastric cancer public-omics secondary analysis. It is not a formal risk-of-bias instrument and does not replace TRIPOD, TRIPOD+AI, PROBAST, or journal-specific reporting requirements.

Table 6. GC-PROVE appraisal rubric for gastric cancer public-omics secondary analysis

GC-PROVE domain	Score 0	Score 1	Score 2	Reviewer question
Problem anchoring	Pipeline- or gene-set-driven study without a clear question	Broad biological theme but vague clinical or mechanistic target	Explicit clinical, biological, or validation question with intended claim type	What decision, mechanism, or validation gap does the analysis address?
Resource provenance	Dataset names missing or unreconstructable	Accessions reported but filtering, endpoints, or overlap incompletely described	Accessions, versions, sample filters, endpoint definitions, missingness, and cohort overlap are auditable	Can another analyst reconstruct the cohort?
Omics workflow transparency	Preprocessing, feature selection, or model formula unavailable	Partial workflow reported, but code, versions, or tuning details incomplete	Normalization, gene mapping, batch correction, feature selection, model formula, coefficients, software versions, and code are provided	Are preprocessing and modeling choices reproducible?
Validation hierarchy	Discovery cohort only or validation after model rebuilding	External or biological support present but model/thresholds are not fully locked	Locked external validation plus relevant tissue, protein, spatial, functional, or clinical support proportional to the claim	Is the claim appropriately limited by its validation level?
Evidence restraint	Conclusions imply clinical readiness from retrospective public data	Limitations mentioned but translational language remains stronger than evidence	Findings are framed as candidates or hypotheses unless clinical utility has been directly tested	Is clinical language matched to the evidence?
Prediction-model rigor	Discrimination or Kaplan-Meier separation only	Some calibration, DCA, or clinical comparator is reported	Locked formula, calibration, DCA, clinical comparator, and intended-use context are all reported	Does the model improve on clinical predictors in an interpretable context?
High-resolution omics rigor	Single-cell or spatial data used as decorative confirmation	Cell annotation or spatial context discussed with limited validation	Integration choices, annotation uncertainty, spatial platform limits, and orthogonal support are explicitly addressed	Are cell states and spatial niches independently supported?
Reproducibility	No code, coefficients, cutoffs, package	Partial code or parameters available	Executable code, model object, feature	Could the central figure or risk score be

	versions, or random seed		list, coefficients, cutoffs, versions, seeds, and derived materials are available	reproduced?
