# Supplementary material for: Public omics data reuse in gastric cancer: from discovery abundance to translational evidence
Source: Front Oncol. 2026 Oct 26;16:1974260. doi: 10.3389/fonc.2026.1974260 (PMC13746168; doi:10.3389/fonc.2026.1974260)
Supplement: Supplementary file 1 [file DataSheet1.zip › supplementary/Supplementary_Materials.docx]

**Supplementary Materials**

The supplementary files support a methodological narrative review and its contextual database scans. They are not PRISMA systematic-review products. All supplementary files, search logs, PMID-level record sets, record-level publication-date data and generation scripts are archived at https://doi.org/10.5281/zenodo.23066111 (concept DOI, which resolves to the latest version of the package).

| **Item** | **Content** | **Format** |
| --- | --- | --- |
| Supplementary File S1 | PubMed search strings and QueryTranslation outputs, supplied as an editable document. | S1 search log |
| Supplementary File S2 | Record-set documentation: year-assignment rule, per-topic counts at both retrieval dates, complete PMID-level record sets for all five queries, and the record-level publication-date data file (Supplementary_File_S2_year_assignment_record_level.csv) that supports the year-assignment explanation. | S2 documentation + 6 CSV files |
| Supplementary File S3 | Coding rules, extraction procedure, retrieval settings, and interpretation notes for the abstract-level reporting-signal screen, including the samples, coding definitions and denominators behind both Table S1 and Table S3. | S3 coding rules |
| Supplementary Table S1 | Supportive PubMed scan counts by query topic. | CSV + DOCX |
| Supplementary Table S2 | Supportive publication-trend data by year (see Supplementary File S2 for the year-assignment rule). | CSV + DOCX |
| Supplementary Table S3 | Title/abstract reporting-signal screen of the 100 most recent prognostic-signature records, with per-record coding. | CSV + DOCX |
| Supplementary Table S4 | Open public-metadata gap check, with PubMed indexing status, search-corpus membership, and topic eligibility recorded as separate fields. | CSV + DOCX |
| Supplementary Table S5 | Prognostic-signature comparison: theme, development cohort, validation design, endpoint, patient independence, clinical comparison, and reported performance assessment for each of 11 studies, verified against the full text of each cited article and linked to its PubMed identifier and source location. | CSV + DOCX |
| Supplementary Table S6 | Single-cell and spatial method comparison in gastric cancer. | CSV + DOCX |
| Supplementary Table S7 | Marker-level detail: treatment setting, assay, cutoff, jurisdiction and evidence date for each gastric cancer treatment-selection marker. | CSV + DOCX |
| Supplementary GC-PROVE Checklist | Practical appraisal aid for authors and reviewers; not a validated risk-of-bias instrument. | DOCX + PDF |
| Supplementary Figure S1 | Supportive publication trends for public-omics, prognostic-signature, single-cell, spatial-omics, and AI or machine-learning gastric cancer literature, 2019-2026. | PDF |
