# Supplementary material for: Public omics data reuse in gastric cancer: from discovery abundance to translational evidence
Source: Front Oncol. 2026 Oct 26;16:1974260. doi: 10.3389/fonc.2026.1974260 (PMC13746168; doi:10.3389/fonc.2026.1974260)
Supplement: Supplementary file 1 [file DataSheet1.zip › supplementary/Supplementary_Table_S1_pubmed_scan_counts.docx]

**Supplementary Table S1. Supportive PubMed scan counts.**

| **topic** | **query** | **pubmed_count** | **screened_records** | **query_translation** | **external_validation_mentions** | **single_cell_mentions** | **spatial_mentions** | **ai_ml_mentions** |
| --- | --- | --- | --- | --- | --- | --- | --- | --- |
| Public omics secondary analysis in gastric cancer | (("gastric cancer"[Title/Abstract] OR "stomach adenocarcinoma"[Title/Abstract] OR "stomach cancer"[Title/Abstract]) AND (TCGA[Title/Abstract] OR GEO[Title/Abstract] OR "public database"[Title/Abstract] OR "public datasets"[Title/Abstract] OR bioinformatics[Title/Abstract] OR "secondary analysis"[Title/Abstract] OR transcriptome[Title/Abstract]) NOT retracted publication[Publication Type]) AND ("2019/01/01"[Date - Publication] : "2026/06/30"[Date - Publication]) | 4416 | 100 | ((("gastric cancer"[Title/Abstract] OR "stomach adenocarcinoma"[Title/Abstract] OR "stomach cancer"[Title/Abstract]) AND ("TCGA"[Title/Abstract] OR "GEO"[Title/Abstract] OR "public database"[Title/Abstract] OR "public datasets"[Title/Abstract] OR "bioinformatics"[Title/Abstract] OR "secondary analysis"[Title/Abstract] OR "transcriptome"[Title/Abstract])) NOT "retracted publication"[Publication Type]) AND 2019/01/01:2026/06/30[Date - Publication] | 2 | 2 | 0 | 2 |
| Prognostic signatures and risk models | (("gastric cancer"[Title/Abstract] OR "stomach adenocarcinoma"[Title/Abstract]) AND ("risk signature"[Title/Abstract] OR "prognostic signature"[Title/Abstract] OR "prognostic model"[Title/Abstract] OR nomogram[Title/Abstract] OR "survival model"[Title/Abstract]) NOT retracted publication[Publication Type]) AND ("2019/01/01"[Date - Publication] : "2026/06/30"[Date - Publication]) | 1812 | 100 | ((("gastric cancer"[Title/Abstract] OR "stomach adenocarcinoma"[Title/Abstract]) AND ("risk signature"[Title/Abstract] OR "prognostic signature"[Title/Abstract] OR "prognostic model"[Title/Abstract] OR "nomogram"[Title/Abstract] OR "survival model"[Title/Abstract])) NOT "retracted publication"[Publication Type]) AND 2019/01/01:2026/06/30[Date - Publication] | 0 | 2 | 0 | 3 |
| External validation language in prognostic modeling | (("gastric cancer"[Title/Abstract] OR "stomach adenocarcinoma"[Title/Abstract]) AND ("risk signature"[Title/Abstract] OR "prognostic signature"[Title/Abstract] OR "prognostic model"[Title/Abstract] OR nomogram[Title/Abstract]) AND ("external validation"[Title/Abstract] OR "validation cohort"[Title/Abstract] OR "independent cohort"[Title/Abstract] OR GEO[Title/Abstract]) NOT retracted publication[Publication Type]) AND ("2019/01/01"[Date - Publication] : "2026/06/30"[Date - Publication]) | 604 | 100 | ((("gastric cancer"[Title/Abstract] OR "stomach adenocarcinoma"[Title/Abstract]) AND ("risk signature"[Title/Abstract] OR "prognostic signature"[Title/Abstract] OR "prognostic model"[Title/Abstract] OR "nomogram"[Title/Abstract]) AND ("external validation"[Title/Abstract] OR "validation cohort"[Title/Abstract] OR "independent cohort"[Title/Abstract] OR "GEO"[Title/Abstract])) NOT "retracted publication"[Publication Type]) AND 2019/01/01:2026/06/30[Date - Publication] | 1 | 6 | 0 | 10 |
| Single-cell gastric cancer omics | (("gastric cancer"[Title/Abstract] OR "stomach adenocarcinoma"[Title/Abstract]) AND ("single-cell"[Title/Abstract] OR scRNA[Title/Abstract] OR "single cell"[Title/Abstract]) NOT retracted publication[Publication Type]) AND ("2019/01/01"[Date - Publication] : "2026/06/30"[Date - Publication]) | 876 | 100 | ((("gastric cancer"[Title/Abstract] OR "stomach adenocarcinoma"[Title/Abstract]) AND ("single-cell"[Title/Abstract] OR "scRNA"[Title/Abstract] OR "single-cell"[Title/Abstract])) NOT "retracted publication"[Publication Type]) AND 2019/01/01:2026/06/30[Date - Publication] | 0 | 52 | 12 | 3 |
| Spatial omics in gastric cancer | (("gastric cancer"[Title/Abstract] OR "stomach adenocarcinoma"[Title/Abstract]) AND ("spatial transcriptomics"[Title/Abstract] OR "spatial omics"[Title/Abstract] OR "spatial multi-omics"[Title/Abstract] OR "multiplex immunofluorescence"[Title/Abstract] OR Xenium[Title/Abstract] OR Visium[Title/Abstract] OR CosMx[Title/Abstract] OR MERFISH[Title/Abstract] OR "Stereo-seq"[Title/Abstract]) NOT retracted publication[Publication Type]) AND ("2019/01/01"[Date - Publication] : "2026/06/30"[Date - Publication]) | 148 | 100 | ((("gastric cancer"[Title/Abstract] OR "stomach adenocarcinoma"[Title/Abstract]) AND ("spatial transcriptomics"[Title/Abstract] OR "spatial omics"[Title/Abstract] OR "spatial multi-omics"[Title/Abstract] OR "multiplex immunofluorescence"[Title/Abstract] OR "Xenium"[Title/Abstract] OR "Visium"[Title/Abstract] OR "CosMx"[Title/Abstract] OR "MERFISH"[Title/Abstract] OR "Stereo-seq"[Title/Abstract])) NOT "retracted publication"[Publication Type]) AND 2019/01/01:2026/06/30[Date - Publication] | 1 | 18 | 33 | 4 |
| AI and machine learning public-omics prognosis | (("gastric cancer"[Title/Abstract] OR "stomach adenocarcinoma"[Title/Abstract]) AND ("machine learning"[Title/Abstract] OR "artificial intelligence"[Title/Abstract] OR "deep learning"[Title/Abstract] OR XGBoost[Title/Abstract] OR transformer[Title/Abstract]) AND (prognosis[Title/Abstract] OR survival[Title/Abstract] OR "risk model"[Title/Abstract] OR "public database"[Title/Abstract] OR transcriptome[Title/Abstract] OR TCGA[Title/Abstract] OR GEO[Title/Abstract]) NOT retracted publication[Publication Type]) AND ("2019/01/01"[Date - Publication] : "2026/06/30"[Date - Publication]) | 636 | 100 | ((("gastric cancer"[Title/Abstract] OR "stomach adenocarcinoma"[Title/Abstract]) AND ("machine learning"[Title/Abstract] OR "artificial intelligence"[Title/Abstract] OR "deep learning"[Title/Abstract] OR "XGBoost"[Title/Abstract] OR "transformer"[Title/Abstract]) AND ("prognosis"[Title/Abstract] OR "survival"[Title/Abstract] OR "risk model"[Title/Abstract] OR "public database"[Title/Abstract] OR "transcriptome"[Title/Abstract] OR "TCGA"[Title/Abstract] OR "GEO"[Title/Abstract])) NOT "retracted publication"[Publication Type]) AND 2019/01/01:2026/06/30[Date - Publication] | 0 | 1 | 0 | 67 |
| Reproducibility and reporting in omics/bioinformatics | (("reproducible research"[Title/Abstract] OR reproducibility[Title/Abstract] OR "code availability"[Title/Abstract] OR "data leakage"[Title/Abstract] OR "batch effect"[Title/Abstract] OR "external validation"[Title/Abstract] OR calibration[Title/Abstract]) AND (omics[Title/Abstract] OR genomics[Title/Abstract] OR transcriptomics[Title/Abstract] OR bioinformatics[Title/Abstract] OR "prediction model"[Title/Abstract]) NOT retracted publication[Publication Type]) AND ("2019/01/01"[Date - Publication] : "2026/06/30"[Date - Publication]) | 14536 | 100 | ((("reproducible research"[Title/Abstract] OR "reproducibility"[Title/Abstract] OR "code availability"[Title/Abstract] OR "data leakage"[Title/Abstract] OR "batch effect"[Title/Abstract] OR "external validation"[Title/Abstract] OR "calibration"[Title/Abstract]) AND ("omics"[Title/Abstract] OR "genomics"[Title/Abstract] OR "transcriptomics"[Title/Abstract] OR "bioinformatics"[Title/Abstract] OR "prediction model"[Title/Abstract])) NOT "retracted publication"[Publication Type]) AND 2019/01/01:2026/06/30[Date - Publication] | 13 | 3 | 10 | 6 |
