# Supplementary material for: Public omics data reuse in gastric cancer: from discovery abundance to translational evidence
Source: Front Oncol. 2026 Oct 26;16:1974260. doi: 10.3389/fonc.2026.1974260 (PMC13746168; doi:10.3389/fonc.2026.1974260)
Supplement: Supplementary file 1 [file DataSheet1.zip › supplementary/Supplementary_Table_S2_publication_trends.docx]

**Supplementary Table S2. Supportive publication-trend data.**

| **topic** | **year** | **pubmed_count** | **query** |
| --- | --- | --- | --- |
| Risk models | 2019 | 63 | (("gastric cancer"[Title/Abstract] OR "stomach adenocarcinoma"[Title/Abstract]) AND ("risk signature"[Title/Abstract] OR "prognostic signature"[Title/Abstract] OR "prognostic model"[Title/Abstract] OR nomogram[Title/Abstract] OR "survival model"[Title/Abstract]) NOT retracted publication[Publication Type]) AND ("2019/01/01"[Date - Publication] : "2019/12/31"[Date - Publication]) |
| Risk models | 2020 | 137 | (("gastric cancer"[Title/Abstract] OR "stomach adenocarcinoma"[Title/Abstract]) AND ("risk signature"[Title/Abstract] OR "prognostic signature"[Title/Abstract] OR "prognostic model"[Title/Abstract] OR nomogram[Title/Abstract] OR "survival model"[Title/Abstract]) NOT retracted publication[Publication Type]) AND ("2020/01/01"[Date - Publication] : "2020/12/31"[Date - Publication]) |
| Risk models | 2021 | 236 | (("gastric cancer"[Title/Abstract] OR "stomach adenocarcinoma"[Title/Abstract]) AND ("risk signature"[Title/Abstract] OR "prognostic signature"[Title/Abstract] OR "prognostic model"[Title/Abstract] OR nomogram[Title/Abstract] OR "survival model"[Title/Abstract]) NOT retracted publication[Publication Type]) AND ("2021/01/01"[Date - Publication] : "2021/12/31"[Date - Publication]) |
| Risk models | 2022 | 321 | (("gastric cancer"[Title/Abstract] OR "stomach adenocarcinoma"[Title/Abstract]) AND ("risk signature"[Title/Abstract] OR "prognostic signature"[Title/Abstract] OR "prognostic model"[Title/Abstract] OR nomogram[Title/Abstract] OR "survival model"[Title/Abstract]) NOT retracted publication[Publication Type]) AND ("2022/01/01"[Date - Publication] : "2022/12/31"[Date - Publication]) |
| Risk models | 2023 | 314 | (("gastric cancer"[Title/Abstract] OR "stomach adenocarcinoma"[Title/Abstract]) AND ("risk signature"[Title/Abstract] OR "prognostic signature"[Title/Abstract] OR "prognostic model"[Title/Abstract] OR nomogram[Title/Abstract] OR "survival model"[Title/Abstract]) NOT retracted publication[Publication Type]) AND ("2023/01/01"[Date - Publication] : "2023/12/31"[Date - Publication]) |
| Risk models | 2024 | 344 | (("gastric cancer"[Title/Abstract] OR "stomach adenocarcinoma"[Title/Abstract]) AND ("risk signature"[Title/Abstract] OR "prognostic signature"[Title/Abstract] OR "prognostic model"[Title/Abstract] OR nomogram[Title/Abstract] OR "survival model"[Title/Abstract]) NOT retracted publication[Publication Type]) AND ("2024/01/01"[Date - Publication] : "2024/12/31"[Date - Publication]) |
| Risk models | 2025 | 368 | (("gastric cancer"[Title/Abstract] OR "stomach adenocarcinoma"[Title/Abstract]) AND ("risk signature"[Title/Abstract] OR "prognostic signature"[Title/Abstract] OR "prognostic model"[Title/Abstract] OR nomogram[Title/Abstract] OR "survival model"[Title/Abstract]) NOT retracted publication[Publication Type]) AND ("2025/01/01"[Date - Publication] : "2025/12/31"[Date - Publication]) |
| Risk models | 2026 | 168 | (("gastric cancer"[Title/Abstract] OR "stomach adenocarcinoma"[Title/Abstract]) AND ("risk signature"[Title/Abstract] OR "prognostic signature"[Title/Abstract] OR "prognostic model"[Title/Abstract] OR nomogram[Title/Abstract] OR "survival model"[Title/Abstract]) NOT retracted publication[Publication Type]) AND ("2026/01/01"[Date - Publication] : "2026/06/30"[Date - Publication]) |
| Single-cell | 2019 | 11 | (("gastric cancer"[Title/Abstract] OR "stomach adenocarcinoma"[Title/Abstract]) AND ("single-cell"[Title/Abstract] OR scRNA[Title/Abstract] OR "single cell"[Title/Abstract]) NOT retracted publication[Publication Type]) AND ("2019/01/01"[Date - Publication] : "2019/12/31"[Date - Publication]) |
| Single-cell | 2020 | 22 | (("gastric cancer"[Title/Abstract] OR "stomach adenocarcinoma"[Title/Abstract]) AND ("single-cell"[Title/Abstract] OR scRNA[Title/Abstract] OR "single cell"[Title/Abstract]) NOT retracted publication[Publication Type]) AND ("2020/01/01"[Date - Publication] : "2020/12/31"[Date - Publication]) |
| Single-cell | 2021 | 38 | (("gastric cancer"[Title/Abstract] OR "stomach adenocarcinoma"[Title/Abstract]) AND ("single-cell"[Title/Abstract] OR scRNA[Title/Abstract] OR "single cell"[Title/Abstract]) NOT retracted publication[Publication Type]) AND ("2021/01/01"[Date - Publication] : "2021/12/31"[Date - Publication]) |
| Single-cell | 2022 | 74 | (("gastric cancer"[Title/Abstract] OR "stomach adenocarcinoma"[Title/Abstract]) AND ("single-cell"[Title/Abstract] OR scRNA[Title/Abstract] OR "single cell"[Title/Abstract]) NOT retracted publication[Publication Type]) AND ("2022/01/01"[Date - Publication] : "2022/12/31"[Date - Publication]) |
| Single-cell | 2023 | 134 | (("gastric cancer"[Title/Abstract] OR "stomach adenocarcinoma"[Title/Abstract]) AND ("single-cell"[Title/Abstract] OR scRNA[Title/Abstract] OR "single cell"[Title/Abstract]) NOT retracted publication[Publication Type]) AND ("2023/01/01"[Date - Publication] : "2023/12/31"[Date - Publication]) |
| Single-cell | 2024 | 188 | (("gastric cancer"[Title/Abstract] OR "stomach adenocarcinoma"[Title/Abstract]) AND ("single-cell"[Title/Abstract] OR scRNA[Title/Abstract] OR "single cell"[Title/Abstract]) NOT retracted publication[Publication Type]) AND ("2024/01/01"[Date - Publication] : "2024/12/31"[Date - Publication]) |
| Single-cell | 2025 | 310 | (("gastric cancer"[Title/Abstract] OR "stomach adenocarcinoma"[Title/Abstract]) AND ("single-cell"[Title/Abstract] OR scRNA[Title/Abstract] OR "single cell"[Title/Abstract]) NOT retracted publication[Publication Type]) AND ("2025/01/01"[Date - Publication] : "2025/12/31"[Date - Publication]) |
| Single-cell | 2026 | 173 | (("gastric cancer"[Title/Abstract] OR "stomach adenocarcinoma"[Title/Abstract]) AND ("single-cell"[Title/Abstract] OR scRNA[Title/Abstract] OR "single cell"[Title/Abstract]) NOT retracted publication[Publication Type]) AND ("2026/01/01"[Date - Publication] : "2026/06/30"[Date - Publication]) |
| Spatial omics | 2019 | 1 | (("gastric cancer"[Title/Abstract] OR "stomach adenocarcinoma"[Title/Abstract]) AND ("spatial transcriptomics"[Title/Abstract] OR "spatial omics"[Title/Abstract] OR "spatial multi-omics"[Title/Abstract] OR "multiplex immunofluorescence"[Title/Abstract] OR Xenium[Title/Abstract] OR Visium[Title/Abstract] OR CosMx[Title/Abstract] OR MERFISH[Title/Abstract] OR "Stereo-seq"[Title/Abstract]) NOT retracted publication[Publication Type]) AND ("2019/01/01"[Date - Publication] : "2019/12/31"[Date - Publication]) |
| Spatial omics | 2020 | 1 | (("gastric cancer"[Title/Abstract] OR "stomach adenocarcinoma"[Title/Abstract]) AND ("spatial transcriptomics"[Title/Abstract] OR "spatial omics"[Title/Abstract] OR "spatial multi-omics"[Title/Abstract] OR "multiplex immunofluorescence"[Title/Abstract] OR Xenium[Title/Abstract] OR Visium[Title/Abstract] OR CosMx[Title/Abstract] OR MERFISH[Title/Abstract] OR "Stereo-seq"[Title/Abstract]) NOT retracted publication[Publication Type]) AND ("2020/01/01"[Date - Publication] : "2020/12/31"[Date - Publication]) |
| Spatial omics | 2021 | 1 | (("gastric cancer"[Title/Abstract] OR "stomach adenocarcinoma"[Title/Abstract]) AND ("spatial transcriptomics"[Title/Abstract] OR "spatial omics"[Title/Abstract] OR "spatial multi-omics"[Title/Abstract] OR "multiplex immunofluorescence"[Title/Abstract] OR Xenium[Title/Abstract] OR Visium[Title/Abstract] OR CosMx[Title/Abstract] OR MERFISH[Title/Abstract] OR "Stereo-seq"[Title/Abstract]) NOT retracted publication[Publication Type]) AND ("2021/01/01"[Date - Publication] : "2021/12/31"[Date - Publication]) |
| Spatial omics | 2022 | 10 | (("gastric cancer"[Title/Abstract] OR "stomach adenocarcinoma"[Title/Abstract]) AND ("spatial transcriptomics"[Title/Abstract] OR "spatial omics"[Title/Abstract] OR "spatial multi-omics"[Title/Abstract] OR "multiplex immunofluorescence"[Title/Abstract] OR Xenium[Title/Abstract] OR Visium[Title/Abstract] OR CosMx[Title/Abstract] OR MERFISH[Title/Abstract] OR "Stereo-seq"[Title/Abstract]) NOT retracted publication[Publication Type]) AND ("2022/01/01"[Date - Publication] : "2022/12/31"[Date - Publication]) |
| Spatial omics | 2023 | 16 | (("gastric cancer"[Title/Abstract] OR "stomach adenocarcinoma"[Title/Abstract]) AND ("spatial transcriptomics"[Title/Abstract] OR "spatial omics"[Title/Abstract] OR "spatial multi-omics"[Title/Abstract] OR "multiplex immunofluorescence"[Title/Abstract] OR Xenium[Title/Abstract] OR Visium[Title/Abstract] OR CosMx[Title/Abstract] OR MERFISH[Title/Abstract] OR "Stereo-seq"[Title/Abstract]) NOT retracted publication[Publication Type]) AND ("2023/01/01"[Date - Publication] : "2023/12/31"[Date - Publication]) |
| Spatial omics | 2024 | 31 | (("gastric cancer"[Title/Abstract] OR "stomach adenocarcinoma"[Title/Abstract]) AND ("spatial transcriptomics"[Title/Abstract] OR "spatial omics"[Title/Abstract] OR "spatial multi-omics"[Title/Abstract] OR "multiplex immunofluorescence"[Title/Abstract] OR Xenium[Title/Abstract] OR Visium[Title/Abstract] OR CosMx[Title/Abstract] OR MERFISH[Title/Abstract] OR "Stereo-seq"[Title/Abstract]) NOT retracted publication[Publication Type]) AND ("2024/01/01"[Date - Publication] : "2024/12/31"[Date - Publication]) |
| Spatial omics | 2025 | 53 | (("gastric cancer"[Title/Abstract] OR "stomach adenocarcinoma"[Title/Abstract]) AND ("spatial transcriptomics"[Title/Abstract] OR "spatial omics"[Title/Abstract] OR "spatial multi-omics"[Title/Abstract] OR "multiplex immunofluorescence"[Title/Abstract] OR Xenium[Title/Abstract] OR Visium[Title/Abstract] OR CosMx[Title/Abstract] OR MERFISH[Title/Abstract] OR "Stereo-seq"[Title/Abstract]) NOT retracted publication[Publication Type]) AND ("2025/01/01"[Date - Publication] : "2025/12/31"[Date - Publication]) |
| Spatial omics | 2026 | 49 | (("gastric cancer"[Title/Abstract] OR "stomach adenocarcinoma"[Title/Abstract]) AND ("spatial transcriptomics"[Title/Abstract] OR "spatial omics"[Title/Abstract] OR "spatial multi-omics"[Title/Abstract] OR "multiplex immunofluorescence"[Title/Abstract] OR Xenium[Title/Abstract] OR Visium[Title/Abstract] OR CosMx[Title/Abstract] OR MERFISH[Title/Abstract] OR "Stereo-seq"[Title/Abstract]) NOT retracted publication[Publication Type]) AND ("2026/01/01"[Date - Publication] : "2026/06/30"[Date - Publication]) |
| AI/ML | 2019 | 7 | (("gastric cancer"[Title/Abstract] OR "stomach adenocarcinoma"[Title/Abstract]) AND ("machine learning"[Title/Abstract] OR "artificial intelligence"[Title/Abstract] OR "deep learning"[Title/Abstract] OR XGBoost[Title/Abstract] OR transformer[Title/Abstract]) AND (prognosis[Title/Abstract] OR survival[Title/Abstract] OR "risk model"[Title/Abstract] OR "public database"[Title/Abstract] OR transcriptome[Title/Abstract] OR TCGA[Title/Abstract] OR GEO[Title/Abstract]) NOT retracted publication[Publication Type]) AND ("2019/01/01"[Date - Publication] : "2019/12/31"[Date - Publication]) |
| AI/ML | 2020 | 22 | (("gastric cancer"[Title/Abstract] OR "stomach adenocarcinoma"[Title/Abstract]) AND ("machine learning"[Title/Abstract] OR "artificial intelligence"[Title/Abstract] OR "deep learning"[Title/Abstract] OR XGBoost[Title/Abstract] OR transformer[Title/Abstract]) AND (prognosis[Title/Abstract] OR survival[Title/Abstract] OR "risk model"[Title/Abstract] OR "public database"[Title/Abstract] OR transcriptome[Title/Abstract] OR TCGA[Title/Abstract] OR GEO[Title/Abstract]) NOT retracted publication[Publication Type]) AND ("2020/01/01"[Date - Publication] : "2020/12/31"[Date - Publication]) |
| AI/ML | 2021 | 42 | (("gastric cancer"[Title/Abstract] OR "stomach adenocarcinoma"[Title/Abstract]) AND ("machine learning"[Title/Abstract] OR "artificial intelligence"[Title/Abstract] OR "deep learning"[Title/Abstract] OR XGBoost[Title/Abstract] OR transformer[Title/Abstract]) AND (prognosis[Title/Abstract] OR survival[Title/Abstract] OR "risk model"[Title/Abstract] OR "public database"[Title/Abstract] OR transcriptome[Title/Abstract] OR TCGA[Title/Abstract] OR GEO[Title/Abstract]) NOT retracted publication[Publication Type]) AND ("2021/01/01"[Date - Publication] : "2021/12/31"[Date - Publication]) |
| AI/ML | 2022 | 75 | (("gastric cancer"[Title/Abstract] OR "stomach adenocarcinoma"[Title/Abstract]) AND ("machine learning"[Title/Abstract] OR "artificial intelligence"[Title/Abstract] OR "deep learning"[Title/Abstract] OR XGBoost[Title/Abstract] OR transformer[Title/Abstract]) AND (prognosis[Title/Abstract] OR survival[Title/Abstract] OR "risk model"[Title/Abstract] OR "public database"[Title/Abstract] OR transcriptome[Title/Abstract] OR TCGA[Title/Abstract] OR GEO[Title/Abstract]) NOT retracted publication[Publication Type]) AND ("2022/01/01"[Date - Publication] : "2022/12/31"[Date - Publication]) |
| AI/ML | 2023 | 87 | (("gastric cancer"[Title/Abstract] OR "stomach adenocarcinoma"[Title/Abstract]) AND ("machine learning"[Title/Abstract] OR "artificial intelligence"[Title/Abstract] OR "deep learning"[Title/Abstract] OR XGBoost[Title/Abstract] OR transformer[Title/Abstract]) AND (prognosis[Title/Abstract] OR survival[Title/Abstract] OR "risk model"[Title/Abstract] OR "public database"[Title/Abstract] OR transcriptome[Title/Abstract] OR TCGA[Title/Abstract] OR GEO[Title/Abstract]) NOT retracted publication[Publication Type]) AND ("2023/01/01"[Date - Publication] : "2023/12/31"[Date - Publication]) |
| AI/ML | 2024 | 127 | (("gastric cancer"[Title/Abstract] OR "stomach adenocarcinoma"[Title/Abstract]) AND ("machine learning"[Title/Abstract] OR "artificial intelligence"[Title/Abstract] OR "deep learning"[Title/Abstract] OR XGBoost[Title/Abstract] OR transformer[Title/Abstract]) AND (prognosis[Title/Abstract] OR survival[Title/Abstract] OR "risk model"[Title/Abstract] OR "public database"[Title/Abstract] OR transcriptome[Title/Abstract] OR TCGA[Title/Abstract] OR GEO[Title/Abstract]) NOT retracted publication[Publication Type]) AND ("2024/01/01"[Date - Publication] : "2024/12/31"[Date - Publication]) |
| AI/ML | 2025 | 208 | (("gastric cancer"[Title/Abstract] OR "stomach adenocarcinoma"[Title/Abstract]) AND ("machine learning"[Title/Abstract] OR "artificial intelligence"[Title/Abstract] OR "deep learning"[Title/Abstract] OR XGBoost[Title/Abstract] OR transformer[Title/Abstract]) AND (prognosis[Title/Abstract] OR survival[Title/Abstract] OR "risk model"[Title/Abstract] OR "public database"[Title/Abstract] OR transcriptome[Title/Abstract] OR TCGA[Title/Abstract] OR GEO[Title/Abstract]) NOT retracted publication[Publication Type]) AND ("2025/01/01"[Date - Publication] : "2025/12/31"[Date - Publication]) |
| AI/ML | 2026 | 122 | (("gastric cancer"[Title/Abstract] OR "stomach adenocarcinoma"[Title/Abstract]) AND ("machine learning"[Title/Abstract] OR "artificial intelligence"[Title/Abstract] OR "deep learning"[Title/Abstract] OR XGBoost[Title/Abstract] OR transformer[Title/Abstract]) AND (prognosis[Title/Abstract] OR survival[Title/Abstract] OR "risk model"[Title/Abstract] OR "public database"[Title/Abstract] OR transcriptome[Title/Abstract] OR TCGA[Title/Abstract] OR GEO[Title/Abstract]) NOT retracted publication[Publication Type]) AND ("2026/01/01"[Date - Publication] : "2026/06/30"[Date - Publication]) |
