# Supplementary material for: Public omics data reuse in gastric cancer: from discovery abundance to translational evidence
Source: Front Oncol. 2026 Oct 26;16:1974260. doi: 10.3389/fonc.2026.1974260 (PMC13746168; doi:10.3389/fonc.2026.1974260)
Supplement: Supplementary file 1 [file DataSheet1.zip › supplementary/Supplementary_Table_S3_signature_reporting_signals.docx]

**Supplementary Table S3. Illustrative abstract-level reporting signals.**

| **pmid** | **year** | **title** | **doi** | **mentions_external_validation** | **mentions_calibration** | **mentions_decision_curve** | **mentions_clinical_comparator** | **mentions_experimental_validation** | **mentions_code_availability** |
| --- | --- | --- | --- | --- | --- | --- | --- | --- | --- |
| 41521567 | 2026 | Cost-effectiveness analysis of sugemalimab plus chemotherapy for the first-line treatment of advanced gastric cancer or gastroesophageal junction cancer. | 10.1080/21645515.2025.2611471 | False | False | False | False | False | False |
| 42068630 | 2026 | Integrative multi-omics analysis identifies stromal-immune crosstalk as a determinant of immunotherapy efficacy and establishes a prognostic signature in gastric cancer. | 10.1016/j.compbiolchem.2026.109095 | True | False | False | True | True | False |
| 42368553 | 2026 | A prognostic senescence-related long non-coding RNA signature identifies cold and hot tumors of stomach adenocarcinoma. | 10.3892/ol.2026.15705 | False | False | False | True | True | False |
| 42367221 | 2026 | Based on comprehensive analysis of ScRNA-seq and bulk RNA-seq, identification of palmitoylation-related genes as biomarkers to evaluate prognosis and immune landscape in stomach adenocarcinoma. | 10.1007/s10616-026-01014-7 | True | False | False | False | False | False |
| 42205459 | 2026 | CDX2-UPK1B-PIK3IP1-PI3K/AKT signaling axis regulates gastric cancer cell invasion and migration and influences patient prognosis. | 10.3892/etm.2026.13179 | False | False | False | False | True | False |
| 42127605 | 2026 | CT assessment of body composition and liver fat fraction predicts prognosis in gastric cancer. | 10.1016/j.ejso.2026.111855 | True | False | True | True | False | False |
| 42380878 | 2026 | Perioperative NLR/PLR dynamic phenotypes and prediction of postoperative recurrence in locally advanced gastric cancer: a retrospective cohort study. | 10.1186/s12885-026-16431-2 | False | False | False | False | False | False |
| 42371227 | 2026 | Expression, prognostic value and function of LMBR1L in gastric cancer. | 10.1007/s00438-026-02420-4 | False | False | False | True | True | False |
| 42365091 | 2026 | Comprehensive bioinformatics analysis identifies KYNU as a novel Helicobacter pylori -associated biomarker with prognostic and therapeutic potential in gastric cancer. | 10.1038/s41598-026-59676-4 | True | False | False | False | True | False |
| 42363944 | 2026 | Pharmacogenomic characterization of a uric acid metabolism-related signature associated with prognosis and drug sensitivity in gastric cancer. | 10.1007/s00210-026-05609-x | False | False | False | False | True | False |
| 42360660 | 2026 | Disulfidptosis-related signatures identify SPARC+/CYR61+ stromal cells mediating immunosuppression in gastric cancer. | 10.1007/s13402-026-01231-4 | False | False | False | False | False | False |
| 42360349 | 2026 | Dual-energy computed tomography-derived extracellular volume fraction and spectral quantitative parameters for predicting early recurrence after gastrectomy: insights from a multicenter study. | 10.1007/s00261-026-05645-4 | True | True | True | True | False | False |
| 42372557 | 2026 | Preoperative prediction of lymphovascular and perineural invasion in locally advanced gastric cancer via CT habitat analysis and deep learning: A dual-center study. | 10.1016/j.ejrad.2026.113041 | True | False | True | True | False | False |
| 42332305 | 2026 | A stemness-associated prognostic model identifies CXCR4 as a key regulator of M2 macrophage polarization via lactate in stomach adenocarcinoma. | 10.1007/s12672-026-05446-7 | False | False | False | False | True | False |
| 42326762 | 2026 | Exosome-Related Gene Signature Predicts Prognosis and Immunotherapy in Gastric Cancer. | 10.1155/ijog/1884334 | False | False | False | True | True | False |
| 42221812 | 2026 | Integration of single-cell regulon atlas and bulk RNA-seq for individualized prognostic prediction in stomach adenocarcinoma. | 10.1016/j.isci.2026.116100 | False | False | False | False | False | False |
| 42394695 | 2026 | Distinct roles of nutritional and inflammatory signatures in predicting pathological response versus long-term survival in locally advanced gastric cancer treated with neoadjuvant immunotherapy. | 10.3389/fonc.2026.1774606 | False | True | False | True | False | False |
| 42382127 | 2026 | Single-cell and pseudobulk analyses reveal hidden mitochondrial expression imbalance in gastric cancer. | 10.3389/fgene.2026.1826214 | False | False | False | False | False | False |
| 42327341 | 2026 | Cross-Strand Chimeric RNA Signature Predicts Prognosis and Identifies Tumor Immune Microenvironment Associations in Gastric Cancer. | 10.1155/humu/4428673 | True | False | False | True | True | False |
| 42307886 | 2026 | CXCL8 producing macrophages shape gastric cancer outcomes. | 10.1007/s12672-026-05452-9 | True | False | False | True | False | False |
| 42299544 | 2026 | Establishment and validation of the nomogram of prognostic risk factors in women with advanced gastric cancer. | 10.1097/MD.0000000000049248 | True | True | False | True | False | False |
| 42285781 | 2026 | Habitat and Peritumoral Radiomics with Clinical Variables for Predicting Lymphovascular Invasion in Gastric Cancer: A Cross-Sectional Study. | 10.1016/j.acra.2026.05.006 | True | True | True | True | False | False |
| 42369062 | 2026 | Preoperative electrolyte-based metabolic biomarkers for prognostic assessment in gastric cancer: a multicenter study. | 10.3389/fendo.2026.1852688 | True | True | True | True | False | False |
| 42277474 | 2026 | A novel composite score based on NLR, PLR, and PNI for constructing a nomogram to predict overall survival in patients with gastric cancer. | 10.1007/s12672-026-05337-x | True | True | True | True | False | False |
| 42344700 | 2026 | Optimizing gastric cancer treatment: the role of LODDs in lymph node staging. | 10.3389/fonc.2026.1828429 | False | True | True | True | False | False |
| 42306581 | 2026 | miR-1287-5p as a Biomarker for Predicting Lymph Node Metastasis in Gastric Cancer Patients. | 10.2147/IJGM.S609960 | False | True | False | True | False | False |
| 42282859 | 2026 | A Nomogram Integrating HOXA9 Expression and Clinicopathological Factors for Predicting Postoperative Recurrence in Gastric Cancer: A Single-Center Retrospective Study. | 10.2147/OTT.S596270 | False | False | False | True | True | False |
| 42251241 | 2026 | Construction of a nomogram for preoperative prediction of occult peritoneal metastasis in advanced gastric cancer based on circulating tumor cells and fibrinogen to albumin ratio. | 10.1007/s12672-026-05371-9 | False | False | True | True | False | False |
| 42327759 | 2026 | Macrophage morphology in the tumor microenvironment predicts metachronous liver metastasis in gastric cancer: establishment and validation of a predictive model. | 10.3389/fimmu.2026.1770436 | True | True | True | True | False | False |
| 42326319 | 2026 | Prognostic significance and model evaluation of the modified advanced lung cancer inflammation index in locally advanced resectable gastric cancer patients with neoadjuvant chemotherapy. | 10.3389/fonc.2026.1821468 | True | True | True | True | False | False |
| 42381781 | 2026 | Development and Validation of a Nomogram to Predict Liver Metastases in Patients With Gastric Cancer. | 10.1007/s13193-026-02604-9 | True | True | True | True | False | False |
| 42316957 | 2026 | Preoperative hypoalbuminemia as a prognostic factor in patients with gastric cancer undergoing radical gastrectomy: Construction of a nomogram. | 10.1177/03000605261457301 | False | False | False | True | False | False |
| 42226295 | 2026 | Immune-inflammatory-fibrinogen score as a novel prognostic biomarker in patients with gastric cancer undergoing radical gastrectomy: a multicenter study. | 10.1186/s12957-026-04439-8 | True | True | True | True | False | False |
| 42054940 | 2026 | Integrating multi-omics data for enhanced prognosis prediction in gastric cancer post-neoadjuvant therapy. | 10.1016/j.ejso.2026.111837 | True | False | False | True | False | False |
| 42019279 | 2026 | Deciphering IFN signaling in gastric cancer: A single-cell and bulk transcriptomic integration reveals CXCR4 as a key immunomodulator and prognostic determinant. | 10.1016/j.tranon.2026.102778 | False | False | False | False | True | False |
| 42013548 | 2026 | Single-Cell sequencing investigation of endoplasmic reticulum stress-related genes in gastric cancer prognostic models and identification of NOX5 as a novel therapeutic target. | 10.1016/j.tranon.2026.102780 | False | False | False | True | True | False |
| 41890218 | 2026 | Multi-omics analysis reveals AR as a potential prognostic factor and immune-related therapeutic target in gastric cancer. | 10.1016/j.bbrep.2026.102537 | False | False | False | False | False | False |
| 42305463 | 2026 | Liquid-liquid phase separation-related gene signature characterizes prognostic subtypes and therapeutic sensitivities in gastric cancer. | 10.21037/tcr-2025-1-2846 | False | True | True | True | False | False |
| 42294502 | 2026 | The expression and clinical significance of miR-635 and miR-519d in gastric cancer patients. | 10.3389/fgene.2026.1782820 | False | False | False | True | False | False |
| 42278393 | 2026 | MYC_V1-Related Genes Affect Gastric Cancer Proliferation by Regulating Energy Metabolism and Analysis of Therapeutic Targets. | 10.3390/ijms27114862 | False | True | False | True | True | False |
| 42211756 | 2026 | Integrative Multiomics Analysis Reveals Tumor-Associated Macrophage Heterogeneity and a Prognostic Signature in Gastric Cancer. | 10.1155/humu/6130883 | False | False | False | False | False | False |
| 42161649 | 2026 | [Construction of a predictive model for the efficacy of immune checkpoint inhibitors in locally advanced or metastatic gastric cancer and esophagogastric junction cancer]. | 10.3760/cma.j.cn441530-20250513-00186 | False | True | False | True | False | False |
| 42174453 | 2026 | Prognostic model for patients with hepatoid adenocarcinoma of the stomach. | 10.1186/s12876-026-04933-z | False | False | False | True | False | False |
| 42227538 | 2026 | Leveraging a Disulfidptosis-related LncRNA Prognostic Signature to Indicate Immune Features and Drug Sensitivity of Gastric Cancer Based on Machine Learning. | 10.2174/0115680096427239260209013858 | False | False | False | False | True | False |
| 42159885 | 2026 | Constructing and investigating a disulfidptosis-associated LncRNA signature for prognostic prediction in gastric cancer. | 10.1007/s12672-026-05209-4 | False | False | True | False | True | False |
| 42157210 | 2026 | A nomogram integrating p53 and Ki-67 expression for predicting recurrence risk in postoperative early gastric cancer. | 10.1186/s12957-026-04402-7 | True | True | True | True | False | False |
| 42141239 | 2026 | Integrated single-cell and bulk transcriptomic analyses unveil a necroptosis-related prognostic model and its association with tumor microenvironment remodeling in gastric cancer. | 10.1007/s12672-026-05109-7 | True | False | False | True | False | False |
| 41797008 | 2026 | Integrating machine learning of bulk and single cell RNA data to characterize immune mechanism of m6A related mitophagy genes in malignant gastric cancer. | 10.1016/j.cmpb.2026.109307 | False | False | False | False | True | False |
| 42134865 | 2026 | Investigating the Impact of Bisphenol A on Gastric Cancer Through Network Toxicology and Molecular Docking. | 10.1177/10915818261451203 | False | False | False | False | False | False |
| 42152648 | 2026 | Construction of an Extracellular Matrix-related Gene Model for Predicting Prognosis and Immune Features in Gastric Cancer. | 10.2174/0115665240429901251230094544 | False | False | False | True | False | False |
| 42107026 | 2026 | FDX1 as a predictive biomarker and therapeutic target for lymph node metastasis in gastric cancer. | 10.1007/s10238-026-02160-0 | True | False | False | False | True | False |
| 42106891 | 2026 | Exploring the prognostic role of senescence-related genes in gastric cancer through multi-omics integration and machine learning. | 10.1186/s40246-026-00979-y | False | False | False | False | True | False |
| 42180700 | 2026 | The predictive value of preoperative clinical laboratory indicators combined with sarcopenia for postoperative complications of gastric cancer. | 10.3389/fmed.2026.1740026 | False | True | True | True | False | False |
| 42116352 | 2026 | Revealing the cuproptosis-lncRNA nexus in gastric cancer: A multicohort observational study. | 10.1097/MD.0000000000048729 | False | False | True | True | False | False |
| 42104552 | 2026 | Lipid-Soluble Signaling Molecular Model Predicts Prognosis and Immunotherapy Response in Gastric Cancer. | 10.1002/lipd.70063 | True | False | False | True | True | False |
| 42193082 | 2026 | Cellular Origins and Context-Dependent Prognostic Effects of Lactate Metabolism Genes Reveal Novel Molecular Subtypes in Gastric Cancer. | 10.3390/cimb48050477 | True | False | False | False | False | False |
| 42069898 | 2026 | Fatty acid β-oxidation-associated prognostic model in gastric cancer and functional validation of IL-6 as a potential therapeutic target. | 10.1038/s41598-026-50112-1 | True | False | False | True | True | False |
| 42067686 | 2026 | Focus on M2-TAMs and gastric cancer: a Mendelian randomization and bioinformatics analysis. | 10.1007/s00438-026-02422-2 | True | False | False | False | True | False |
| 41748954 | 2026 | Predictive nomogram for postsurgical gastroparesis syndrome and survival outcomes following distal gastrectomy: an in-depth analysis. | 10.1007/s00464-026-12673-0 | True | True | False | True | False | False |
| 41740269 | 2026 | Non-invasive prediction of Ki-67 expression in gastric cancer using AI-based dual-energy CT: a multicenter study. | 10.1016/j.ejrad.2026.112742 | True | True | True | True | False | False |
| 41665852 | 2026 | Comparative Cost-Effectiveness Analysis of Multiple First-Line Treatments for HER2-Negative Unresectable Advanced or Recurrent Gastric Cancer in Japan. | 10.1007/s41669-025-00634-7 | False | False | False | False | False | False |
| 41566126 | 2026 | Establishment and Validation of Prediction Models for Lymph Node Metastasis and Long-Term Survival in Patients with T1b Early Gastric Cancer: A Retrospective Cohort Study with 10-Year Follow-Up. | 10.1245/s10434-025-18883-5 | False | True | True | True | False | False |
| 41296174 | 2026 | Development and validation of a cuproptosis-related gene signature for predicting prognosis and drug sensitivity in gastric cancer. | 10.1007/s12094-025-04132-4 | True | False | False | True | True | False |
| 42180959 | 2026 | Preoperative prediction of HER2 expression in gastric cancer based on an enhanced CT radiomics model. | 10.21037/tcr-2026-1-0034 | True | True | True | False | True | False |
| 42180937 | 2026 | Identification of a novel ion channel-related gene signature to predict prognosis and immune response of gastric cancer. | 10.21037/tcr-2025-1-2874 | False | False | False | True | True | False |
| 42180883 | 2026 | Integrating multiple omics and machine learning to reveal the prognostic value of endoplasmic reticulum stress gene FKBP10 in gastric cancer. | 10.21037/tcr-2025-1-2817 | True | False | False | False | True | False |
| 42169944 | 2026 | Identification of immunogenic cell death-related prognostic genes in gastric cancer. | 10.21037/jgo-2025-aw-831 | False | False | False | True | False | False |
| 42169909 | 2026 | Development and external validation of a prognostic nomogram for cancer-specific survival in elderly patients with early-stage gastric cancer. | 10.21037/jgo-2025-aw-815 | True | True | True | True | False | False |
| 42145746 | 2026 | A multicenter retrospective study: risk factors and nomogram construction for pathological escalation of gastric low-grade intraepithelial neoplasia. | 10.3389/fmed.2026.1800141 | True | True | True | True | False | False |
| 42056898 | 2026 | A nutrition-focused model for predicting postoperative complications in locally advanced gastric cancer after neoadjuvant chemotherapy: a retrospective cohort study. | 10.1186/s12876-026-04852-z | True | True | True | True | False | False |
| 42053674 | 2026 | Integrative systems biology and drug repurposing reveal key regulatory hubs and a prognostic signature in gastric cancer. | 10.1007/s12672-026-04989-z | False | False | False | False | False | False |
| 42032043 | 2026 | A novel risk stratification strategy for precision prevention of gastric cancer based on clinicopathological features and IGFBP7. | 10.1038/s41598-026-49722-6 | True | False | False | True | True | False |
| 41991641 | 2026 | Comparison of the long-term impact of laparoscopy and open surgery on the prognosis of patients with locally advanced gastric cancer. | 10.1038/s41598-026-48276-x | False | False | False | False | False | False |
| 41986425 | 2026 | FNDC1 is closely related to poor prognosis and immune cell infiltration in gastric cancer. | 10.1038/s41598-026-46622-7 | True | False | False | True | True | False |
| 41981105 | 2026 | Exploration of m7G-related immune genes and construction of a prognostic risk model in gastric cancer. | 10.1038/s41598-026-47456-z | False | False | False | True | False | False |
| 42039888 | 2026 | The preoperative albumin-to-carcinoembryonic antigen ratio (ACR) predicts prognosis and facilitates risk stratification in gastric cancer: a retrospective cohort study. | 10.3389/fnut.2026.1789564 | False | True | False | True | False | False |
| 42038391 | 2026 | A novel combined model integrating collagen properties, radiomics and clinical data to predict gastric cancer prognosis. | 10.3389/fonc.2026.1801350 | False | False | True | False | False | False |
| 41963816 | 2026 | Preoperative clinical-imaging nomogram for peritoneal metastasis in gastric cancer: a retrospective cohort study. | 10.1186/s12876-026-04785-7 | False | True | True | True | False | False |
| 41982336 | 2026 | Identification of an m7G-Related lncRNA Signature for Prognostic Prediction and Immune Landscape Characterization in Early Gastric Cancer. | 10.2147/OTT.S582487 | False | False | False | False | False | False |
| 42005437 | 2026 | Development and validation of a novel prognostic model for gastric signet ring cell carcinoma based on inflammation-nutrition indicators. | 10.3389/fnut.2026.1623570 | False | True | True | True | False | False |
| 41928259 | 2026 | Clinicopathological features and prognosis of HER2-low gastric cancer: a real-world retrospective cohort study. | 10.1186/s12957-026-04323-5 | False | True | False | True | False | False |
| 41942360 | 2026 | A Nomogram for Predicting Metachronous Gastric Cancer After Endoscopic Submucosal Dissection of Early Gastric Cancer Following Successful Helicobacter pylori Eradication. | 10.5230/jgc.2026.26.e21 | False | False | False | True | False | False |
| 41923711 | 2026 | Construction of a Prognostic Risk Model for Helicobacter pylori Infection in Gastric Cancer Patients and Immunological Analysis. | 10.1002/cnr2.70511 | True | False | False | False | False | False |
| 41837463 | 2026 | Pathway Mutation Accumulate Perturbation Score: A prognostic and predictive biomarker for immunotherapy in advanced gastric cancer. | 10.17219/acem/206937 | True | False | False | False | False | False |
| 41565492 | 2026 | Preoperative Prediction of Perineural Invasion and Survival in Gastric Cancer Using Extracellular Volume Fraction and Dual-Energy CT Quantitative Parameters: A Dual-Center Study. | 10.1016/j.acra.2026.01.006 | True | True | True | True | False | False |
| 41191583 | 2026 | Comparative efficacy and safety of S-1 plus oxaliplatin with sintilimab vs. paclitaxel-S-1-oxaliplatin and docetaxel-oxaliplatin-5-fluorouracil as first-line therapy for advanced gastric cancer. | 10.1097/CAD.0000000000001782 | False | False | False | False | False | False |
| 40960514 | 2026 | CT-based delta-radiomics nomogram to assess tumor regression grade in locally advanced gastric cancer patients following neoadjuvant chemotherapy. | 10.1007/s00261-025-05171-9 | True | False | False | True | False | False |
| 40392448 | 2026 | A Novel Telomere Maintenance Gene-Related Model for Prognosis Prediction in Gastric Cancer. | 10.1007/s10528-025-11132-0 | False | False | False | False | False | False |
| 41969508 | 2026 | Construction of competing-risk nomograms and identification of optimal candidates for aggressive therapy in gastric cancer with peritoneal metastasis: a population-based study. | 10.21037/tcr-2025-1-2692 | True | True | True | True | False | False |
| 41969477 | 2026 | A prediction model of gastric cancer based on M2-like tumor-associated macrophage infiltration verified by immunohistochemistry. | 10.21037/tcr-2025-aw-2213 | True | False | False | True | True | False |
| 41969473 | 2026 | A pretreatment 18F-FDG PET/CT-based nomogram for the differential diagnosis of primary gastric cancer and primary gastric diffuse large B-cell lymphoma. | 10.21037/tcr-2025-aw-2277 | False | True | True | True | False | False |
| 41969439 | 2026 | Association of CAB39L gene methylation in peripheral blood leukocytes with the risk, chemotherapy efficacy and prognosis of gastric cancer and construction of risk prediction model. | 10.21037/tcr-2025-2044 | False | False | False | True | False | False |
| 41917621 | 2026 | Collagen type I alpha 2 acts as a potential diagnostic biomarker and therapeutic targets for the prognosis in gastric cancer. | 10.1007/s12672-026-04924-2 | True | True | False | True | True | False |
| 41880007 | 2026 | A tissue-to-blood mRNA classifier enables early detection of gastric cancer risk in precancerous lesions. | 10.1007/s00114-026-02080-9 | True | False | False | True | False | False |
| 41930144 | 2026 | Single-Cell and Multi-Omics-Based Characterization of Gastric Cancer Identifies TPP1 as a Potential Target for Gastric Cancer Progression and Treatment. | 10.32604/or.2026.070208 | False | False | False | False | True | False |
| 41897678 | 2026 | A Radiomics-Clinical Nomogram for Pre-Treatment Prediction of Neoadjuvant Chemotherapy Response in Locally Advanced Gastric Cancer. | 10.3390/diagnostics16060945 | True | True | True | True | False | False |
| 41938584 | 2026 | A prognostic nomogram integrating preoperative IBI and MAR with clinicopathological factors for gastric cancer patients after radical gastrectomy. | 10.3389/fsurg.2026.1793710 | False | True | False | True | False | False |
| 41899595 | 2026 | A Pathomics-Based Prognostic Model for Disease-Free Survival in Resected Gastric Cancer. | 10.3390/cancers18060993 | True | True | True | True | False | False |
| 42007160 | 2026 | Correlation of immune-inflammatory and tumor markers with lymph node metastasis in gastric cancer. | 10.62347/OHHU1152 | True | True | True | True | False | False |
| 41909689 | 2026 | A personalized prognostic model based on preoperative body composition and nutritional parameters for gastric cancer patients receiving neoadjuvant chemotherapy. | 10.3389/fimmu.2026.1759292 | True | False | True | True | False | False |
