# Supplementary material for: Public omics data reuse in gastric cancer: from discovery abundance to translational evidence
Source: Front Oncol. 2026 Oct 26;16:1974260. doi: 10.3389/fonc.2026.1974260 (PMC13746168; doi:10.3389/fonc.2026.1974260)
Supplement: Supplementary file 1 [file DataSheet1.zip › supplementary/Supplementary_Table_S4_public_metadata_scan.docx]

**Supplementary Table S4. Public metadata scan with indexing status, search-corpus membership, and topic eligibility recorded as separate fields**

| **source** | **query** | **title** | **year** | **venue** | **doi** | **pmid_in_metadata** | **pmid_verified** | **pubmed_indexing_status** | **search_corpus_membership** | **topic_eligible_for_review** | **original_field_likely_not_in_pubmed** | **url** |
| --- | --- | --- | --- | --- | --- | --- | --- | --- | --- | --- | --- | --- |
| Crossref | gastric cancer public omics TCGA GEO bioinformatics | Identification of functional lncRNAs in gastric cancer by integrative analysis of GEO and TCGA data | 2019 | Journal of Cellular Biochemistry | 10.1002/jcb.29058 |  | 31135068 | indexed_in_pubmed | yes | yes | True | https://doi.org/10.1002/jcb.29058 |
| Crossref | gastric cancer public omics TCGA GEO bioinformatics | The expression of miR-375 in prostate cancer: A study based on GEO, TCGA data and bioinformatics analysis | 2019 | Pathology - Research and Practice | 10.1016/j.prp.2019.03.004 |  | 30879885 | indexed_in_pubmed | no | no | True | https://doi.org/10.1016/j.prp.2019.03.004 |
| Crossref | gastric cancer public omics TCGA GEO bioinformatics | Systematic Identification of Multi Omics-based Biomarkers in <i>KEAP1</i> Mutated TCGA Lung Adenocarcinoma | 2019 | Journal of Cancer | 10.7150/jca.35489 |  | 31839815 | indexed_in_pubmed | no | no | True | https://doi.org/10.7150/jca.35489 |
| Crossref | gastric cancer public omics TCGA GEO bioinformatics | Nomograms combined with SERPINE1-related module genes predict overall and recurrence-free survival after curative resection of gastric cancer: a study based on TCGA and GEO data | 2020 | Translational Cancer Research | 10.21037/tcr-20-818 |  | 35117805 | indexed_in_pubmed | yes | yes | True | https://doi.org/10.21037/tcr-20-818 |
| Crossref | gastric cancer public omics TCGA GEO bioinformatics | Computing, Bioinformatics Capabilities Kept Pan-Cancer Project Humming | 2020 | Clinical OMICs | 10.1089/clinomi.07.02.15 |  |  | not_found_by_doi_query | not_determined | no | True | https://doi.org/10.1089/clinomi.07.02.15 |
| Crossref | gastric cancer public omics TCGA GEO bioinformatics | Identifying hub genes of papillary thyroid carcinoma in the TCGA and GEO database using bioinformatics analysis | 2020 | PeerJ | 10.7717/peerj.9120 |  | 32714651 | indexed_in_pubmed | no | no | True | https://doi.org/10.7717/peerj.9120 |
| Crossref | gastric cancer public omics TCGA GEO bioinformatics | Leveraging TCGA gene expression data to build predictive models for cancer drug response | 2020 | BMC Bioinformatics | 10.1186/s12859-020-03690-4 |  | 32998700 | indexed_in_pubmed | no | no | True | https://doi.org/10.1186/s12859-020-03690-4 |
| Crossref | gastric cancer prognostic signature public database | Systematical Analysis of the Cancer Genome Atlas Database Reveals EMCN/MUC15 Combination as a Prognostic Signature for Gastric Cancer | 2020 | Frontiers in Molecular Biosciences | 10.3389/fmolb.2020.00019 |  | 32175327 | indexed_in_pubmed | yes | yes | True | https://doi.org/10.3389/fmolb.2020.00019 |
| Crossref | gastric cancer public omics TCGA GEO bioinformatics | High expression of LUM independently predicts poor prognosis in gastric cancer: a bioinformatics study combining TCGA and GEO datasets | 2021 | All Life | 10.1080/26895293.2021.2000894 |  |  | not_found_by_doi_query | not_determined | yes | True | https://doi.org/10.1080/26895293.2021.2000894 |
| Crossref | gastric cancer public omics TCGA GEO bioinformatics | The 2nd Conference and Workshop of The Cancer Genome Atlas (TCGA) in India: Towards Team Science for Multi-omics Cancer Research in South Asia | 2021 | ecancermedicalscience | 10.3332/ecancer.2021.ed111 |  | 34221123 | indexed_in_pubmed | no | no | True | https://doi.org/10.3332/ecancer.2021.ed111 |
| OpenAlex | gastric cancer prognostic signature public database | The Chinese Society of Clinical Oncology (CSCO): Clinical guidelines for the diagnosis and treatment of gastric cancer, 2021 | 2021 | Cancer Communications | 10.1002/cac2.12193 |  | 34197702 | indexed_in_pubmed | no | no | True | https://doi.org/10.1002/cac2.12193 |
| Crossref | gastric cancer prognostic signature public database | Integrating 13 Microarrays to Construct a 6 RNA-binding proteins Prognostic Signature for Gastric Cancer patients | 2021 | Journal of Cancer | 10.7150/jca.57225 |  | 34234866 | indexed_in_pubmed | yes | yes | True | https://doi.org/10.7150/jca.57225 |
| Crossref | gastric cancer prognostic signature public database | Characterization of the Prognostic m6A-Related lncRNA Signature in Gastric Cancer | 2021 | Frontiers in Oncology | 10.3389/fonc.2021.630260 |  | 33928026 | indexed_in_pubmed | yes | yes | True | https://doi.org/10.3389/fonc.2021.630260 |
| Crossref | gastric cancer prognostic signature public database | Eleven immune-gene pairs signature associated with TP53 predicting the overall survival of gastric cancer: a retrospective analysis of large sample and multicenter from public database | 2021 | Journal of Translational Medicine | 10.1186/s12967-021-02846-x |  | 33926488 | indexed_in_pubmed | yes | yes | True | https://doi.org/10.1186/s12967-021-02846-x |
| Crossref | gastric cancer prognostic signature public database | Identification and external validation of a prognostic signature associated with DNA repair genes in gastric cancer | 2021 | Scientific Reports | 10.1038/s41598-021-86504-8 |  | 33785812 | indexed_in_pubmed | yes | yes | True | https://doi.org/10.1038/s41598-021-86504-8 |
| Crossref | gastric cancer prognostic signature public database | Development of a novel autophagy-related gene prognostic signature for gastric cancer | 2021 | Translational Cancer Research | 10.21037/tcr-21-191 |  | 35116589 | indexed_in_pubmed | yes | yes | True | https://doi.org/10.21037/tcr-21-191 |
| Crossref | stomach adenocarcinoma single-cell spatial omics | Single-cell omics decipher tumor evolution | 2021 | Medicine in Omics | 10.1016/j.meomic.2021.100006 |  |  | not_found_by_doi_query | not_determined | no | True | https://doi.org/10.1016/j.meomic.2021.100006 |
| Crossref | gastric cancer public omics TCGA GEO bioinformatics | Clinical significance and prospective molecular mechanism of NUF2 in gastric cancer exploration: A comprehensive study based on the GeneChip, GEO, Oncomine, and TCGA databases | 2022 | Medicine | 10.1097/md.0000000000029802 |  | 35777029 | indexed_in_pubmed | yes | yes | True | https://doi.org/10.1097/md.0000000000029802 |
| Crossref | gastric cancer public omics TCGA GEO bioinformatics | The molecular mechanisms of CTHRC1 in gastric cancer by integrating TCGA, GEO and GSA datasets | 2022 | Frontiers in Genetics | 10.3389/fgene.2022.900124 |  | 35928443 | indexed_in_pubmed | yes | yes | True | https://doi.org/10.3389/fgene.2022.900124 |
| Crossref | gastric cancer public omics TCGA GEO bioinformatics | Prognostic Value and Biological Significance of GUCY1A2 in Gastric Cancer: A Bioinformatics Analysis Base on TCGA Database | 2022 | Trends Journal of Sciences Research | 10.31586/wjcor.2022.412 |  |  | not_found_by_doi_query | not_determined | yes | True | https://doi.org/10.31586/wjcor.2022.412 |
| Crossref | gastric cancer public omics TCGA GEO bioinformatics | Impact of Androgen Receptor Gene Expression in Gastric Cancer: a Meta-Analysis Based on the GEO Database, TCGA Database, and Literature | 2022 | Indian Journal of Surgery | 10.1007/s12262-020-02168-0 |  |  | not_found_by_doi_query | not_determined | yes | True | https://doi.org/10.1007/s12262-020-02168-0 |
| Crossref | gastric cancer prognostic signature public database | Identification of prognostic alternative splicing signature in gastric cancer | 2022 | Archives of Public Health | 10.1186/s13690-022-00894-3 |  | 35614517 | indexed_in_pubmed | yes | yes | True | https://doi.org/10.1186/s13690-022-00894-3 |
| Crossref | gastric cancer prognostic signature public database | Comprehensive bioinformatic analysis reveals a cancer-associated fibroblast gene signature as a poor prognostic factor and potential therapeutic target in gastric cancer | 2022 | BMC Cancer | 10.1186/s12885-022-09736-5 |  | 35739492 | indexed_in_pubmed | yes | yes | True | https://doi.org/10.1186/s12885-022-09736-5 |
| Crossref | gastric cancer prognostic signature public database | A nine–consensus–prognostic –gene–based prognostic signature, recognizing the dichotomized subgroups of gastric cancer patients with different clinical outcomes and therapeutic strategies | 2022 | Frontiers in Genetics | 10.3389/fgene.2022.909175 |  | 36226177 | indexed_in_pubmed | yes | yes | True | https://doi.org/10.3389/fgene.2022.909175 |
| Crossref | stomach adenocarcinoma single-cell spatial omics | Single cell‐ and spatial ‘Omics revolutionize physiology | 2022 | Acta Physiologica | 10.1111/apha.13848 |  | 35656634 | indexed_in_pubmed | no | no | True | https://doi.org/10.1111/apha.13848 |
| Crossref | stomach adenocarcinoma single-cell spatial omics | Abstract 74: Single-cell 3D spatial omics for tumor hypoxia | 2022 | Cancer Research | 10.1158/1538-7445.am2022-74 |  |  | not_found_by_doi_query | not_determined | no | True | https://doi.org/10.1158/1538-7445.am2022-74 |
| Crossref | stomach adenocarcinoma single-cell spatial omics | A Multi-Omics Study on the Effect of Helicobacter Pylori-Related Genes in the Tumor Immunity on Stomach Adenocarcinoma | 2022 | Frontiers in Cellular and Infection Microbiology | 10.3389/fcimb.2022.880636 |  | 35619651 | indexed_in_pubmed | yes | yes | True | https://doi.org/10.3389/fcimb.2022.880636 |
| Crossref | gastric cancer public omics TCGA GEO bioinformatics | TCGAplot: an R package for integrative pan-cancer analysis and visualization of TCGA multi-omics data | 2023 | BMC Bioinformatics | 10.1186/s12859-023-05615-3 |  | 38105215 | indexed_in_pubmed | no | no | True | https://doi.org/10.1186/s12859-023-05615-3 |
| Crossref | gastric cancer public omics TCGA GEO bioinformatics | Prognostic significance of JAM 3 in gastric cancer: An observational study from TCGA and GEO | 2023 | Medicine | 10.1097/md.0000000000033603 |  | 37115068 | indexed_in_pubmed | yes | yes | True | https://doi.org/10.1097/md.0000000000033603 |
| Crossref | gastric cancer public omics TCGA GEO bioinformatics | Gastric Cancer Biomarker Candidates Identified by Machine Learning and Integrative Bioinformatics: Toward Personalized Medicine | 2023 | OMICS: A Journal of Integrative Biology | 10.1089/omi.2023.0015 |  | 37229622 | indexed_in_pubmed | yes | yes | True | https://doi.org/10.1089/omi.2023.0015 |
| Crossref | gastric cancer public omics TCGA GEO bioinformatics | Bioinformatics analysis of hedgehog interacting protein in colorectal cancer: a study based on GEO data and TCGA data | 2023 | BMC Gastroenterology | 10.1186/s12876-023-02867-4 |  | 37568084 | indexed_in_pubmed | no | no | True | https://doi.org/10.1186/s12876-023-02867-4 |
| Crossref | gastric cancer public omics TCGA GEO bioinformatics | Lessons in gastric adenocarcinoma from TCGA | 2023 | Nature Reviews Cancer | 10.1038/s41568-023-00606-1 |  | 37474705 | indexed_in_pubmed | no | yes | True | https://doi.org/10.1038/s41568-023-00606-1 |
| Crossref | gastric cancer prognostic signature public database | A novel prognostic and therapeutic target biomarker based on complement-related gene signature in gastric cancer | 2023 | Translational Cancer Research | 10.21037/tcr-23-628 |  | 38192986 | indexed_in_pubmed | yes | yes | True | https://doi.org/10.21037/tcr-23-628 |
| Crossref | gastric cancer prognostic signature public database | Construction of a Six-Gene Prognostic Signature Associated with Notch Signaling Pathway in Gastric Cancer | 2023 | JOURNAL OF BIOLOGICAL REGULATORS AND HOMEOSTATIC AGENTS | 10.23812/j.biol.regul.homeost.agents.20233701.8 |  |  | not_found_by_doi_query | not_determined | yes | True | https://doi.org/10.23812/j.biol.regul.homeost.agents.20233701.8 |
| Crossref | gastric cancer prognostic signature public database | Retracted: Identification of Ferroptosis-Related Long Noncoding RNA and Construction of a Novel Prognostic Signature for Gastric Cancer | 2023 | Disease Markers | 10.1155/2023/9810178 |  | 37387742 | indexed_in_pubmed | yes | yes | True | https://doi.org/10.1155/2023/9810178 |
| Crossref | stomach adenocarcinoma single-cell spatial omics | Computational framework decomposes and annotates single-cell and spatial omics | 2023 | Nature Biotechnology | 10.1038/s41587-023-01658-2 |  | 36797495 | indexed_in_pubmed | no | no | True | https://doi.org/10.1038/s41587-023-01658-2 |
| Crossref | stomach adenocarcinoma single-cell spatial omics | SINGLE-CELL AND SPATIAL OMICS FOR MAPPING CELLULAR SENESCENCE IN HEALTH, AGING AND DISEASE | 2023 | Innovation in Aging | 10.1093/geroni/igad104.1555 |  |  | not_found_by_doi_query | not_determined | no | True | https://doi.org/10.1093/geroni/igad104.1555 |
| Crossref | stomach adenocarcinoma single-cell spatial omics | Microtechnologies for single-cell and spatial multi-omics | 2023 | Nature Reviews Bioengineering | 10.1038/s44222-023-00084-y |  |  | not_found_by_doi_query | not_determined | no | True | https://doi.org/10.1038/s44222-023-00084-y |
| Crossref | stomach adenocarcinoma single-cell spatial omics | Immunological role and clinical prognostic significance of P2RY6 in lung adenocarcinoma: a multi-omics studies and single-cell sequencing analysis | 2023 | World Journal of Surgical Oncology | 10.1186/s12957-023-03216-1 |  | 37880703 | indexed_in_pubmed | no | no | True | https://doi.org/10.1186/s12957-023-03216-1 |
| Crossref | gastric cancer prognostic signature public database | Construction of gastric cancer prognostic signature based on the E26 transcription factor and the identification of novel oncogene ELK3 | 2024 | American Journal of Cancer Research | 10.62347/rvbp7871 |  | 38726274 | indexed_in_pubmed | yes | yes | True | https://doi.org/10.62347/rvbp7871 |
| Crossref | gastric cancer prognostic signature public database | Development of an immunogenic cell death-related lncRNAs signature for prognostic risk assessment in gastric cancer | 2024 | Translational Cancer Research | 10.21037/tcr-24-344 |  | 39262480 | indexed_in_pubmed | yes | yes | True | https://doi.org/10.21037/tcr-24-344 |
| Crossref | gastric cancer prognostic signature public database | Integration of mult-omics and nucleotide metabolism reprogramming signature analysis reveals gastric cancer immunological and prognostic features | 2024 | Cancer Cell International | 10.1186/s12935-024-03396-0 |  | 38880869 | indexed_in_pubmed | yes | yes | True | https://doi.org/10.1186/s12935-024-03396-0 |
| Crossref | gastric cancer prognostic signature public database | Prognostic signature and immune landscape of 5-methylcytosine-related long non-coding RNAs in gastric cancer | 2024 | Heliyon | 10.1016/j.heliyon.2024.e37290 |  | 39323814 | indexed_in_pubmed | yes | yes | True | https://doi.org/10.1016/j.heliyon.2024.e37290 |
| Crossref | gastric cancer prognostic signature public database | Prognostic value and potential function of a novel heme-related LncRNAs signature in gastric cancer | 2024 | Cellular Signalling | 10.1016/j.cellsig.2024.111152 |  | 38548123 | indexed_in_pubmed | yes | yes | True | https://doi.org/10.1016/j.cellsig.2024.111152 |
| Crossref | stomach adenocarcinoma single-cell spatial omics | Optimal transport for single-cell and spatial omics | 2024 | Nature Reviews Methods Primers | 10.1038/s43586-024-00345-z |  |  | not_found_by_doi_query | not_determined | no | True | https://doi.org/10.1038/s43586-024-00345-z |
| Crossref | stomach adenocarcinoma single-cell spatial omics | Exploration of signature based on T cell-related genes in stomach adenocarcinoma by analysis of single cell sequencing data | 2024 | Aging | 10.18632/aging.205687 |  | 38536020 | indexed_in_pubmed | yes | yes | True | https://doi.org/10.18632/aging.205687 |
| Crossref | stomach adenocarcinoma single-cell spatial omics | Editorial: Single-cell and spatial-omics in delineating immune-related diseases | 2024 | Frontiers in Cell and Developmental Biology | 10.3389/fcell.2024.1365242 |  | 38298217 | indexed_in_pubmed | no | no | True | https://doi.org/10.3389/fcell.2024.1365242 |
| Crossref | stomach adenocarcinoma single-cell spatial omics | Optimal transport for single-cell and spatial omics | 2024 | Nature Reviews Methods Primers | 10.1038/s43586-024-00334-2 |  |  | not_found_by_doi_query | not_determined | no | True | https://doi.org/10.1038/s43586-024-00334-2 |
| Crossref | stomach adenocarcinoma single-cell spatial omics | Single-cell and spatial transcriptome characterize coinhibitory cell-cell communications during histological progression of lung adenocarcinoma | 2024 | Frontiers in Immunology | 10.3389/fimmu.2024.1430163 |  | 39474422 | indexed_in_pubmed | no | no | True | https://doi.org/10.3389/fimmu.2024.1430163 |
| Crossref | gastric cancer public omics TCGA GEO bioinformatics | VCAM-1 predicts poor prognosis and modulates immune infiltration in gastric cancer: a TCGA-based bioinformatics study | 2025 | Frontiers in Genetics | 10.3389/fgene.2025.1602929 |  | 40927361 | indexed_in_pubmed | yes | yes | True | https://doi.org/10.3389/fgene.2025.1602929 |
| Crossref | gastric cancer public omics TCGA GEO bioinformatics | Identification and validation of tumor microenvironment-related therapeutic targets in gastric cancer using integrated multi-omics and molecular docking approaches | 2025 | Frontiers in Bioinformatics | 10.3389/fbinf.2025.1654326 |  | 41451347 | indexed_in_pubmed | yes | yes | True | https://doi.org/10.3389/fbinf.2025.1654326 |
| Crossref | stomach adenocarcinoma single-cell spatial omics | Stomach encyclopedia: Combined single-cell and spatial transcriptomics reveal cell diversity and homeostatic regulation of human stomach | 2025 | Cell Reports | 10.1016/j.celrep.2025.116503 |  | 41100257 | indexed_in_pubmed | no | yes | True | https://doi.org/10.1016/j.celrep.2025.116503 |
| Crossref | stomach adenocarcinoma single-cell spatial omics | Cross-domain information fusion for enhanced immune cell population delineation in single-cell spatial-omics data 2975 | 2025 | The Journal of Immunology | 10.1093/jimmun/vkaf283.828 |  |  | not_found_by_doi_query | not_determined | no | True | https://doi.org/10.1093/jimmun/vkaf283.828 |
| Crossref | stomach adenocarcinoma single-cell spatial omics | Single-cell and spatial omics: exploring hypothalamic heterogeneity | 2025 | Neural Regeneration Research | 10.4103/nrr.nrr-d-24-00231 |  | 38993130 | indexed_in_pubmed | no | no | True | https://doi.org/10.4103/nrr.nrr-d-24-00231 |
| Crossref | stomach adenocarcinoma single-cell spatial omics | Correction to: ScInfeR: an efficient method for annotating cell types and sub-types in single-cell RNA-seq, ATAC-seq, and spatial omics | 2025 | Briefings in Bioinformatics | 10.1093/bib/bbaf337 |  | 40580031 | indexed_in_pubmed | no | no | True | https://doi.org/10.1093/bib/bbaf337 |
| Crossref | gastric cancer prognostic signature public database | Expression profiling, molecular subtyping and prognostic signature construction of chemokines and chemokine receptors in gastric cancer | 2026 | Translational Cancer Research | 10.21037/tcr-2026-0590 |  | 42445448 | indexed_in_pubmed | yes | yes | True | https://doi.org/10.21037/tcr-2026-0590 |
| Crossref | gastric cancer prognostic signature public database | Correction: Identification of cuproptosis related subtypes and construction of prognostic signature in gastric cancer | 2026 | Frontiers in Surgery | 10.3389/fsurg.2026.1809226 |  | 41907774 | indexed_in_pubmed | yes | yes | True | https://doi.org/10.3389/fsurg.2026.1809226 |
| Crossref | stomach adenocarcinoma single-cell spatial omics | Abstract 6792 Integrating Single-Cell Omics with Ion Mobility–Enabled MALDI Mass Spectrometry Imaging and Expansion-Assisted Spatial Omics | 2026 | Journal of Biological Chemistry | 10.1016/j.jbc.2026.112639 |  |  | not_found_by_doi_query | not_determined | no | True | https://doi.org/10.1016/j.jbc.2026.112639 |
| Crossref | stomach adenocarcinoma single-cell spatial omics | Progress and Prospects of Single-cell and Spatial Omics in Neurodegenerative Diseases | 2026 | Theoretical and Natural Science | 10.54254/2753-8818/2026.34153 |  |  | not_found_by_doi_query | not_determined | no | True | https://doi.org/10.54254/2753-8818/2026.34153 |
| Crossref | stomach adenocarcinoma single-cell spatial omics | Rising Star: Single Cell Omics Technologies: When Whole Omics Analysis Meets Single Cell Resolution | 2026 | Journal of Molecular Biology | 10.1016/j.jmb.2026.169670 |  | 41621785 | indexed_in_pubmed | no | no | True | https://doi.org/10.1016/j.jmb.2026.169670 |
| OpenAlex | gastric cancer public omics TCGA GEO bioinformatics | TCGA-TCIA Impact on Radiogenomics Cancer Research: A Systematic Review | 2019 | International Journal of Molecular Sciences | 10.3390/ijms20236033 | 31795520 |  | indexed_in_pubmed | no | no | False | https://doi.org/10.3390/ijms20236033 |
| OpenAlex | gastric cancer public omics TCGA GEO bioinformatics | Identification of circRNA–miRNA–mRNA regulatory network in gastric cancer by analysis of microarray data | 2019 | Cancer Cell International | 10.1186/s12935-019-0905-z | 31346318 |  | indexed_in_pubmed | yes | no | False | https://doi.org/10.1186/s12935-019-0905-z |
| OpenAlex | gastric cancer public omics TCGA GEO bioinformatics | RETRACTED ARTICLE: RNA m6A methylation regulates the epithelial mesenchymal transition of cancer cells and translation of Snail | 2019 | Nature Communications | 10.1038/s41467-019-09865-9 | 31061416 |  | indexed_in_pubmed | no | no | False | https://doi.org/10.1038/s41467-019-09865-9 |
| OpenAlex | gastric cancer public omics TCGA GEO bioinformatics | HOXC10 overexpression promotes cell proliferation and migration in gastric cancer | 2019 | Oncology Reports | 10.3892/or.2019.7164 | 31115563 |  | indexed_in_pubmed | yes | no | False | https://doi.org/10.3892/or.2019.7164 |
| OpenAlex | gastric cancer prognostic signature public database | Targeting epigenetic regulators for cancer therapy: mechanisms and advances in clinical trials | 2019 | Signal Transduction and Targeted Therapy | 10.1038/s41392-019-0095-0 | 31871779 |  | indexed_in_pubmed | no | no | False | https://doi.org/10.1038/s41392-019-0095-0 |
| OpenAlex | gastric cancer prognostic signature public database | Correlate tumor mutation burden with immune signatures in human cancers | 2019 | BMC Immunology | 10.1186/s12865-018-0285-5 | 30634925 |  | indexed_in_pubmed | no | no | False | https://doi.org/10.1186/s12865-018-0285-5 |
| OpenAlex | gastric cancer public omics TCGA GEO bioinformatics | Artificial Intelligence (AI)-Based Systems Biology Approaches in Multi-Omics Data Analysis of Cancer | 2020 | Frontiers in Oncology | 10.3389/fonc.2020.588221 | 33154949 |  | indexed_in_pubmed | no | no | False | https://doi.org/10.3389/fonc.2020.588221 |
| OpenAlex | gastric cancer public omics TCGA GEO bioinformatics | RETRACTED ARTICLE: LncRNA BCRT1 promotes breast cancer progression by targeting miR-1303/PTBP3 axis | 2020 | Molecular Cancer | 10.1186/s12943-020-01206-5 | 32384893 |  | indexed_in_pubmed | no | no | False | https://doi.org/10.1186/s12943-020-01206-5 |
| OpenAlex | gastric cancer public omics TCGA GEO bioinformatics | N6-methyladenosine regulates glycolysis of cancer cells through PDK4 | 2020 | Nature Communications | 10.1038/s41467-020-16306-5 | 32444598 |  | indexed_in_pubmed | no | no | False | https://doi.org/10.1038/s41467-020-16306-5 |
| OpenAlex | gastric cancer public omics TCGA GEO bioinformatics | Microenvironment characterization and multi-omics signatures related to prognosis and immunotherapy response of hepatocellular carcinoma | 2020 | Experimental Hematology and Oncology | 10.1186/s40164-020-00165-3 | 32509418 |  | indexed_in_pubmed | no | no | False | https://doi.org/10.1186/s40164-020-00165-3 |
| OpenAlex | gastric cancer public omics TCGA GEO bioinformatics | Bioinformatics Analysis of Key Genes and circRNA‐miRNA‐mRNA Regulatory Network in Gastric Cancer | 2020 | BioMed Research International | 10.1155/2020/2862701 | 32908877 |  | indexed_in_pubmed | yes | yes | False | https://doi.org/10.1155/2020/2862701 |
| OpenAlex | gastric cancer prognostic signature public database | Molecular profiling for precision cancer therapies | 2020 | Genome Medicine | 10.1186/s13073-019-0703-1 | 31937368 |  | indexed_in_pubmed | no | no | False | https://doi.org/10.1186/s13073-019-0703-1 |
| OpenAlex | gastric cancer prognostic signature public database | Cancer health disparities in racial/ethnic minorities in the United States | 2020 | British Journal of Cancer | 10.1038/s41416-020-01038-6 | 32901135 |  | indexed_in_pubmed | no | no | False | https://doi.org/10.1038/s41416-020-01038-6 |
| OpenAlex | gastric cancer prognostic signature public database | Current status of immune checkpoint inhibitors for gastric cancer | 2020 | Gastric Cancer | 10.1007/s10120-020-01090-4 | 32468420 |  | indexed_in_pubmed | no | no | False | https://doi.org/10.1007/s10120-020-01090-4 |
| OpenAlex | gastric cancer prognostic signature public database | ImmuCellAI: A Unique Method for Comprehensive T‐Cell Subsets Abundance Prediction and its Application in Cancer Immunotherapy | 2020 | Advanced Science | 10.1002/advs.201902880 | 32274301 |  | indexed_in_pubmed | no | no | False | https://doi.org/10.1002/advs.201902880 |
| OpenAlex | gastric cancer prognostic signature public database | EMTome: a resource for pan-cancer analysis of epithelial-mesenchymal transition genes and signatures | 2020 | British Journal of Cancer | 10.1038/s41416-020-01178-9 | 33299129 |  | indexed_in_pubmed | no | no | False | https://doi.org/10.1038/s41416-020-01178-9 |
| OpenAlex | gastric cancer prognostic signature public database | Long non-coding RNAs towards precision medicine in gastric cancer: early diagnosis, treatment, and drug resistance | 2020 | Molecular Cancer | 10.1186/s12943-020-01219-0 | 32460771 |  | indexed_in_pubmed | no | no | False | https://doi.org/10.1186/s12943-020-01219-0 |
| OpenAlex | gastric cancer prognostic signature public database | Development and validation of a hypoxia-immune-based microenvironment gene signature for risk stratification in gastric cancer | 2020 | Journal of Translational Medicine | 10.1186/s12967-020-02366-0 | 32410620 |  | indexed_in_pubmed | yes | yes | False | https://doi.org/10.1186/s12967-020-02366-0 |
| OpenAlex | gastric cancer prognostic signature public database | A DNA methylation signature to improve survival prediction of gastric cancer | 2020 | Clinical Epigenetics | 10.1186/s13148-020-0807-x | 31959204 |  | indexed_in_pubmed | yes | yes | False | https://doi.org/10.1186/s13148-020-0807-x |
| OpenAlex | gastric cancer prognostic signature public database | Six-lncRNA Immune Prognostic Signature for Cervical Cancer | 2020 | Frontiers in Genetics | 10.3389/fgene.2020.533628 | 33173530 |  | indexed_in_pubmed | no | no | False | https://doi.org/10.3389/fgene.2020.533628 |
| OpenAlex | stomach adenocarcinoma single-cell spatial omics | EPISCORE: cell type deconvolution of bulk tissue DNA methylomes from single-cell RNA-Seq data | 2020 | Genome biology | 10.1186/s13059-020-02126-9 | 32883324 |  | indexed_in_pubmed | no | no | False | https://doi.org/10.1186/s13059-020-02126-9 |
| OpenAlex | gastric cancer machine learning prognosis transcriptome | The potential role of RNA N6-methyladenosine in Cancer progression | 2020 | Molecular Cancer | 10.1186/s12943-020-01204-7 | 32398132 |  | indexed_in_pubmed | no | no | False | https://doi.org/10.1186/s12943-020-01204-7 |
| OpenAlex | gastric cancer machine learning prognosis transcriptome | Artificial intelligence (AI) and big data in cancer and precision oncology | 2020 | Computational and Structural Biotechnology Journal | 10.1016/j.csbj.2020.08.019 | 32994889 |  | indexed_in_pubmed | no | no | False | https://doi.org/10.1016/j.csbj.2020.08.019 |
| OpenAlex | gastric cancer machine learning prognosis transcriptome | Deep learning in cancer diagnosis, prognosis and treatment selection | 2021 | Genome Medicine | 10.1186/s13073-021-00968-x | 34579788 |  | indexed_in_pubmed | no | no | False | https://doi.org/10.1186/s13073-021-00968-x |
| OpenAlex | gastric cancer public omics TCGA GEO bioinformatics | Integrated single-cell and bulk RNA sequencing analysis identifies a cancer associated fibroblast-related signature for predicting prognosis and therapeutic responses in colorectal cancer | 2021 | Cancer Cell International | 10.1186/s12935-021-02252-9 | 34670584 |  | indexed_in_pubmed | no | no | False | https://doi.org/10.1186/s12935-021-02252-9 |
| OpenAlex | gastric cancer prognostic signature public database | Breast Cancer—Epidemiology, Risk Factors, Classification, Prognostic Markers, and Current Treatment Strategies—An Updated Review | 2021 | Cancers | 10.3390/cancers13174287 | 34503097 |  | indexed_in_pubmed | no | no | False | https://doi.org/10.3390/cancers13174287 |
| OpenAlex | stomach adenocarcinoma single-cell spatial omics | Application of Single-Cell Multi-Omics in Dissecting Cancer Cell Plasticity and Tumor Heterogeneity | 2021 | Frontiers in Molecular Biosciences | 10.3389/fmolb.2021.757024 | 34722635 |  | indexed_in_pubmed | no | no | False | https://doi.org/10.3389/fmolb.2021.757024 |
| OpenAlex | gastric cancer machine learning prognosis transcriptome | Using machine learning approaches for multi-omics data analysis: A review | 2021 | Biotechnology Advances | 10.1016/j.biotechadv.2021.107739 | 33794304 |  | indexed_in_pubmed | no | no | False | https://doi.org/10.1016/j.biotechadv.2021.107739 |
| OpenAlex | gastric cancer machine learning prognosis transcriptome | Clinical applications of artificial intelligence and machine learning in cancer diagnosis: looking into the future | 2021 | Cancer Cell International | 10.1186/s12935-021-01981-1 | 34020642 |  | indexed_in_pubmed | no | no | False | https://doi.org/10.1186/s12935-021-01981-1 |
| OpenAlex | gastric cancer machine learning prognosis transcriptome | Extracellular matrix and its therapeutic potential for cancer treatment | 2021 | Signal Transduction and Targeted Therapy | 10.1038/s41392-021-00544-0 | 33888679 |  | indexed_in_pubmed | no | no | False | https://doi.org/10.1038/s41392-021-00544-0 |
| OpenAlex | gastric cancer public omics TCGA GEO bioinformatics | Proteogenomic characterization of 2002 human cancers reveals pan-cancer molecular subtypes and associated pathways | 2022 | Nature Communications | 10.1038/s41467-022-30342-3 | 35562349 |  | indexed_in_pubmed | no | no | False | https://doi.org/10.1038/s41467-022-30342-3 |
| OpenAlex | stomach adenocarcinoma single-cell spatial omics | Integrated analysis of single-cell and bulk RNA sequencing data reveals a pan-cancer stemness signature predicting immunotherapy response | 2022 | Genome Medicine | 10.1186/s13073-022-01050-w | 35488273 |  | indexed_in_pubmed | no | no | False | https://doi.org/10.1186/s13073-022-01050-w |
| OpenAlex | gastric cancer public omics TCGA GEO bioinformatics | Network-based machine learning approach to predict immunotherapy response in cancer patients | 2022 | Nature Communications | 10.1038/s41467-022-31535-6 | 35764641 |  | indexed_in_pubmed | yes | no | False | https://doi.org/10.1038/s41467-022-31535-6 |
| OpenAlex | gastric cancer prognostic signature public database | A signature for pan-cancer prognosis based on neutrophil extracellular traps | 2022 | Journal for ImmunoTherapy of Cancer | 10.1136/jitc-2021-004210 | 35688556 |  | indexed_in_pubmed | no | no | False | https://doi.org/10.1136/jitc-2021-004210 |
| OpenAlex | stomach adenocarcinoma single-cell spatial omics | Single cell cancer epigenetics | 2022 | Trends in cancer | 10.1016/j.trecan.2022.06.005 | 35821003 |  | indexed_in_pubmed | no | no | False | https://doi.org/10.1016/j.trecan.2022.06.005 |
| OpenAlex | stomach adenocarcinoma single-cell spatial omics | Machine Learning: A New Prospect in Multi-Omics Data Analysis of Cancer | 2022 | Frontiers in Genetics | 10.3389/fgene.2022.824451 | 35154283 |  | indexed_in_pubmed | no | no | False | https://doi.org/10.3389/fgene.2022.824451 |
| OpenAlex | stomach adenocarcinoma single-cell spatial omics | Organoids | 2022 | Nature Reviews Methods Primers | 10.1038/s43586-022-00174-y | 37325195 |  | indexed_in_pubmed | no | no | False | https://doi.org/10.1038/s43586-022-00174-y |
| OpenAlex | gastric cancer machine learning prognosis transcriptome | Liquid biopsy: a step closer to transform diagnosis, prognosis and future of cancer treatments | 2022 | Molecular Cancer | 10.1186/s12943-022-01543-7 | 35303879 |  | indexed_in_pubmed | no | no | False | https://doi.org/10.1186/s12943-022-01543-7 |
| OpenAlex | gastric cancer machine learning prognosis transcriptome | Pan-cancer single-cell analysis reveals the heterogeneity and plasticity of cancer-associated fibroblasts in the tumor microenvironment | 2022 | Nature Communications | 10.1038/s41467-022-34395-2 | 36333338 |  | indexed_in_pubmed | no | no | False | https://doi.org/10.1038/s41467-022-34395-2 |
| OpenAlex | gastric cancer machine learning prognosis transcriptome | Machine Learning-Based Model for the Prognosis of Postoperative Gastric Cancer | 2022 | Cancer Management and Research | 10.2147/cmar.s342352 | 35027848 |  | indexed_in_pubmed | yes | no | False | https://doi.org/10.2147/cmar.s342352 |
| OpenAlex | gastric cancer machine learning prognosis transcriptome | Artificial intelligence in clinical applications for lung cancer: diagnosis, treatment and prognosis | 2022 | Clinical Chemistry and Laboratory Medicine (CCLM) | 10.1515/cclm-2022-0291 | 35771735 |  | indexed_in_pubmed | no | no | False | https://doi.org/10.1515/cclm-2022-0291 |
| OpenAlex | gastric cancer public omics TCGA GEO bioinformatics | Novel biomarkers for early detection of gastric cancer | 2023 | World Journal of Gastroenterology | 10.3748/wjg.v29.i17.2515 | 37213407 |  | indexed_in_pubmed | no | yes | False | https://doi.org/10.3748/wjg.v29.i17.2515 |
| OpenAlex | gastric cancer public omics TCGA GEO bioinformatics | Proteogenomic data and resources for pan-cancer analysis | 2023 | Cancer Cell | 10.1016/j.ccell.2023.06.009 | 37582339 |  | indexed_in_pubmed | no | no | False | https://doi.org/10.1016/j.ccell.2023.06.009 |
| OpenAlex | gastric cancer public omics TCGA GEO bioinformatics | ADAR1-mediated RNA editing of SCD1 drives drug resistance and self-renewal in gastric cancer | 2023 | Nature Communications | 10.1038/s41467-023-38581-8 | 37208334 |  | indexed_in_pubmed | no | no | False | https://doi.org/10.1038/s41467-023-38581-8 |
| OpenAlex | stomach adenocarcinoma single-cell spatial omics | Evolution of immune and stromal cell states and ecotypes during gastric adenocarcinoma progression | 2023 | Cancer Cell | 10.1016/j.ccell.2023.06.005 | 37419119 |  | indexed_in_pubmed | no | no | False | https://doi.org/10.1016/j.ccell.2023.06.005 |
| OpenAlex | gastric cancer machine learning prognosis transcriptome | Neoantigens: promising targets for cancer therapy | 2023 | Signal Transduction and Targeted Therapy | 10.1038/s41392-022-01270-x | 36604431 |  | indexed_in_pubmed | no | no | False | https://doi.org/10.1038/s41392-022-01270-x |
| OpenAlex | gastric cancer prognostic signature public database | Cuproptosis: mechanisms and links with cancers | 2023 | Molecular Cancer | 10.1186/s12943-023-01732-y | 36882769 |  | indexed_in_pubmed | no | no | False | https://doi.org/10.1186/s12943-023-01732-y |
| OpenAlex | stomach adenocarcinoma single-cell spatial omics | Spatially resolved multi-omics highlights cell-specific metabolic remodeling and interactions in gastric cancer | 2023 | Nature Communications | 10.1038/s41467-023-38360-5 | 37164975 |  | indexed_in_pubmed | yes | yes | False | https://doi.org/10.1038/s41467-023-38360-5 |
| OpenAlex | stomach adenocarcinoma single-cell spatial omics | Understanding tumour endothelial cell heterogeneity and function from single-cell omics | 2023 | Nature reviews. Cancer | 10.1038/s41568-023-00591-5 | 37349410 |  | indexed_in_pubmed | no | no | False | https://doi.org/10.1038/s41568-023-00591-5 |
| OpenAlex | stomach adenocarcinoma single-cell spatial omics | Single-Cell RNA Sequencing Unifies Developmental Programs of Esophageal and Gastric Intestinal Metaplasia | 2023 | Cancer Discovery | 10.1158/2159-8290.cd-22-0824 | 36929873 |  | indexed_in_pubmed | no | yes | False | https://doi.org/10.1158/2159-8290.cd-22-0824 |
| OpenAlex | stomach adenocarcinoma single-cell spatial omics | SCAR: Single-cell and Spatially-resolved Cancer Resources | 2023 | Nucleic Acids Research | 10.1093/nar/gkad753 | 37739405 |  | indexed_in_pubmed | no | no | False | https://doi.org/10.1093/nar/gkad753 |
| OpenAlex | stomach adenocarcinoma single-cell spatial omics | Early stage gastric adenocarcinoma: clinical and molecular landscapes | 2023 | Nature Reviews Clinical Oncology | 10.1038/s41571-023-00767-w | 37264184 |  | indexed_in_pubmed | no | no | False | https://doi.org/10.1038/s41571-023-00767-w |
| OpenAlex | gastric cancer machine learning prognosis transcriptome | From patterns to patients: Advances in clinical machine learning for cancer diagnosis, prognosis, and treatment | 2023 | Cell | 10.1016/j.cell.2023.01.035 | 36905928 |  | indexed_in_pubmed | no | no | False | https://doi.org/10.1016/j.cell.2023.01.035 |
| OpenAlex | gastric cancer machine learning prognosis transcriptome | Biomarkers in Cancer Detection, Diagnosis, and Prognosis | 2023 | Sensors | 10.3390/s24010037 | 38202898 |  | indexed_in_pubmed | no | no | False | https://doi.org/10.3390/s24010037 |
| OpenAlex | gastric cancer machine learning prognosis transcriptome | Transfer learning enables predictions in network biology | 2023 | Nature | 10.1038/s41586-023-06139-9 | 37258680 |  | indexed_in_pubmed | no | no | False | https://doi.org/10.1038/s41586-023-06139-9 |
| OpenAlex | gastric cancer machine learning prognosis transcriptome | Machine Learning Methods for Cancer Classification Using Gene Expression Data: A Review | 2023 | Bioengineering | 10.3390/bioengineering10020173 | 36829667 |  | indexed_in_pubmed | no | no | False | https://doi.org/10.3390/bioengineering10020173 |
| OpenAlex | gastric cancer machine learning prognosis transcriptome | Machine Learning Models for the Identification of Prognostic and Predictive Cancer Biomarkers: A Systematic Review | 2023 | International Journal of Molecular Sciences | 10.3390/ijms24097781 | 37175487 |  | indexed_in_pubmed | no | no | False | https://doi.org/10.3390/ijms24097781 |
| OpenAlex | gastric cancer machine learning prognosis transcriptome | Machine learning algorithms reveal potential miRNAs biomarkers in gastric cancer | 2023 | Scientific Reports | 10.1038/s41598-023-32332-x | 37061507 |  | indexed_in_pubmed | yes | yes | False | https://doi.org/10.1038/s41598-023-32332-x |
| OpenAlex | gastric cancer machine learning prognosis transcriptome | Integrated multiomics analysis and machine learning refine molecular subtypes and prognosis for muscle-invasive urothelial cancer | 2023 | Molecular Therapy — Nucleic Acids | 10.1016/j.omtn.2023.06.001 | 37449047 |  | indexed_in_pubmed | no | no | False | https://doi.org/10.1016/j.omtn.2023.06.001 |
| OpenAlex | gastric cancer public omics TCGA GEO bioinformatics | Bioinformatics Analysis and Validation of Potential Markers Associated with Prediction and Prognosis of Gastric Cancer | 2024 | International Journal of Molecular Sciences | 10.3390/ijms25115880 | 38892067 |  | indexed_in_pubmed | yes | yes | False | https://doi.org/10.3390/ijms25115880 |
| OpenAlex | gastric cancer public omics TCGA GEO bioinformatics | Integrating Molecular Perspectives: Strategies for Comprehensive Multi-Omics Integrative Data Analysis and Machine Learning Applications in Transcriptomics, Proteomics, and Metabolomics | 2024 | Biology | 10.3390/biology13110848 | 39596803 |  | indexed_in_pubmed | no | no | False | https://doi.org/10.3390/biology13110848 |
| OpenAlex | gastric cancer prognostic signature public database | Gastric cancer immunosuppressive microenvironment heterogeneity: implications for therapy development | 2024 | Trends in cancer | 10.1016/j.trecan.2024.03.008 | 38600020 |  | indexed_in_pubmed | yes | no | False | https://doi.org/10.1016/j.trecan.2024.03.008 |
| OpenAlex | gastric cancer prognostic signature public database | Integrated analysis of public datasets for the discovery and validation of survival-associated genes in solid tumors | 2024 | The Innovation | 10.1016/j.xinn.2024.100625 | 38706955 |  | indexed_in_pubmed | no | no | False | https://doi.org/10.1016/j.xinn.2024.100625 |
| OpenAlex | stomach adenocarcinoma single-cell spatial omics | Applications of single‑cell omics and spatial transcriptomics technologies in gastric cancer (Review) | 2024 | Oncology Letters | 10.3892/ol.2024.14285 | 38406595 |  | indexed_in_pubmed | yes | yes | False | https://doi.org/10.3892/ol.2024.14285 |
| OpenAlex | stomach adenocarcinoma single-cell spatial omics | Informing immunotherapy with multi-omics driven machine learning | 2024 | npj Digital Medicine | 10.1038/s41746-024-01043-6 | 38486092 |  | indexed_in_pubmed | no | no | False | https://doi.org/10.1038/s41746-024-01043-6 |
| OpenAlex | stomach adenocarcinoma single-cell spatial omics | Single-cell and spatial transcriptome analyses reveal tertiary lymphoid structures linked to tumour progression and immunotherapy response in nasopharyngeal carcinoma | 2024 | Nature Communications | 10.1038/s41467-024-52153-4 | 39231979 |  | indexed_in_pubmed | no | no | False | https://doi.org/10.1038/s41467-024-52153-4 |
| OpenAlex | stomach adenocarcinoma single-cell spatial omics | Spatial and single-cell analyses uncover links between ALKBH1 and tumor-associated macrophages in gastric cancer | 2024 | Cancer Cell International | 10.1186/s12935-024-03232-5 | 38317214 |  | indexed_in_pubmed | yes | yes | False | https://doi.org/10.1186/s12935-024-03232-5 |
| OpenAlex | stomach adenocarcinoma single-cell spatial omics | Macrophage diversity in human cancers: New insight provided by single-cell resolution and spatial context | 2024 | Heliyon | 10.1016/j.heliyon.2024.e28332 | 38571605 |  | indexed_in_pubmed | no | no | False | https://doi.org/10.1016/j.heliyon.2024.e28332 |
| OpenAlex | stomach adenocarcinoma single-cell spatial omics | scCancerExplorer: a comprehensive database for interactively exploring single-cell multi-omics data of human pan-cancer | 2024 | Nucleic Acids Research | 10.1093/nar/gkae1100 | 39558175 |  | indexed_in_pubmed | no | no | False | https://doi.org/10.1093/nar/gkae1100 |
| OpenAlex | stomach adenocarcinoma single-cell spatial omics | Pan‐Cancer Single‐Cell and Spatial‐Resolved Profiling Reveals the Immunosuppressive Role of APOE+ Macrophages in Immune Checkpoint Inhibitor Therapy | 2024 | Advanced Science | 10.1002/advs.202401061 | 38569519 |  | indexed_in_pubmed | no | no | False | https://doi.org/10.1002/advs.202401061 |
| OpenAlex | gastric cancer machine learning prognosis transcriptome | Cancer biomarkers: Emerging trends and clinical implications for personalized treatment | 2024 | Cell | 10.1016/j.cell.2024.02.041 | 38552610 |  | indexed_in_pubmed | no | no | False | https://doi.org/10.1016/j.cell.2024.02.041 |
| OpenAlex | gastric cancer machine learning prognosis transcriptome | Prognosis and Treatment of Gastric Cancer: A 2024 Update | 2024 | Cancers | 10.3390/cancers16091708 | 38730659 |  | indexed_in_pubmed | yes | no | False | https://doi.org/10.3390/cancers16091708 |
| OpenAlex | gastric cancer prognostic signature public database | Helicobacter pylori and gastric cancer: mechanisms and new perspectives | 2025 | Journal of Hematology & Oncology | 10.1186/s13045-024-01654-2 | 39849657 |  | indexed_in_pubmed | no | no | False | https://doi.org/10.1186/s13045-024-01654-2 |
| OpenAlex | stomach adenocarcinoma single-cell spatial omics | Application of single-cell and spatial omics in deciphering cellular hallmarks of cancer drug response and resistance | 2025 | Journal of Hematology & Oncology | 10.1186/s13045-025-01722-1 | 40605007 |  | indexed_in_pubmed | no | no | False | https://doi.org/10.1186/s13045-025-01722-1 |
| Crossref | gastric cancer prognostic signature public database | Liquid-liquid phase separation-related gene signature characterizes prognostic subtypes and therapeutic sensitivities in gastric cancer | 2026 | Translational Cancer Research | 10.21037/tcr-2025-1-2846 |  | 42305463 | indexed_in_pubmed | yes | yes | False | https://doi.org/10.21037/tcr-2025-1-2846 |

Note. Absence from the retrieved search corpus is not the same as absence from PubMed. On re-checking, 121 of 135 records previously flagged as possibly absent from PubMed proved to be indexed there.
