# Supplementary material for: Public omics data reuse in gastric cancer: from discovery abundance to translational evidence
Source: Front Oncol. 2026 Oct 26;16:1974260. doi: 10.3389/fonc.2026.1974260 (PMC13746168; doi:10.3389/fonc.2026.1974260)
Supplement: Supplementary file 1 [file DataSheet1.zip › supplementary/Supplementary_Table_S5_prognostic_signature_comparison.docx]

**Supplementary Table S5. Validation design of representative gastric cancer prognostic-signature studies, verified against the full text of each cited article**

| **Study (Reference)** | **Theme** | **Development cohort** | **Validation cohort / design** | **Endpoint** | **Patient independence** | **Clinical comparison** | **Reported calibration** | **Reported decision curve** | **Where documented in source** | **Source** |
| --- | --- | --- | --- | --- | --- | --- | --- | --- | --- | --- |
| Chang 2023 (37) | Mitochondrial-related genes | TCGA-STAD (training) | External: GSE66229, GSE15459 | Overall survival | Independent external cohorts | TNM stage / clinical variables | Yes | Yes | Methods (data and cohort description); Results and figures for the training and validation cohorts | PMID 36915111 |
| Huang 2023 (38) | CHSY3 / immune infiltration | TCGA plus GEO expression datasets | Multi-dataset expression and prognosis analysis (GSE66229, GSE65801, GSE63089, GSE54129, GSE51575, GSE26901, GSE84433); no single locked signature model | Overall survival | Multiple independent public datasets; no locked model applied to a held-out cohort | Clinicopathological variables | Yes | Not reported | Methods (dataset description); Results (expression and survival analyses) | PMID 37461041 |
| Zhu 2024 (39) | Amino acid metabolism | TCGA-STAD | External: GSE84433, GSE84437, GSE84426; Mendelian randomization component | Overall survival | Independent external cohorts | Not compared | Yes | Yes | Methods (data sources); Results (validation and nomogram) | PMID 39353384 |
| Li 2024 (40) | Pyroptosis / single-cell-informed | TCGA-STAD plus scRNA-seq GSE183904 | External: GSE62254 (Asian Cancer Research Group cohort, n = 300) | Overall survival, disease-free survival | Independent external cohort; scRNA-seq data used for contextualisation rather than validation | TNM stage / clinical variables | Yes | Not reported | Methods (scRNA-seq and bulk data); Results (GSE62254 validation) | PMID 38404710 |
| Cai 2024 (41) | lncRNA / peritoneal recurrence | SYSUCC discovery cohort (12 patients; paired peritoneal, primary tumour, and normal tissue) | SYSUCC validation cohort (231 stage II-III patients) plus interactive analysis against TCGA | Peritoneal recurrence / prognosis | Validation uses a separate patient set at the same institution; not an external multicentre cohort | Clinical variables | Not reported | Not reported | Methods (SYSUCC discovery and validation cohorts); Results (key lncRNA identification) | PMID 39736670 |
| Jiang 2025 (42) | Migrasome-related lncRNAs | TCGA-STAD | Internal only: random training/testing split of the same TCGA cohort | Overall survival | NOT independent: testing set drawn from the same cohort as the training set | TNM stage / clinical variables | Yes | Yes | Methods (random assignment to training and testing sets); Results | PMID 40281132 |
| Zhang 2025 (43) | Antigen-presenting fibroblasts / single-cell | TCGA-STAD | External: GSE62254, GSE15459 | Overall survival | Independent external cohorts | TNM stage / clinical variables | Yes | Not reported | Methods (validation datasets); Results (validation KM and ROC) | PMID 40544264 |
| Yang 2025 (44) | Hypoxia-associated genes | TCGA-STAD | External: GSE15460 (n = 248) | Overall survival | Independent external cohort | Clinicopathological variables | Not reported | Not reported | Methods (validation set); Results (model validation) | PMID 40129974 |
| Jin 2025 (45) | Intratumoral microbiome | TCGA-STAD (training set) | Datasets reported include GSE183904 and GSE62254; external-validation status not explicitly delineated | Overall survival | Not clearly delineated in the source article | TNM stage / clinical variables | Yes | Not reported | Methods (data sources); Results (training-set risk model) | PMID 40677704 |
| Yang 2025 (46) | Multiple machine-learning algorithms | TCGA-STAD | External: GSE84437, GSE26253 | Overall survival | Independent external cohorts | Univariate and multivariate Cox analysis with clinical variables | Not reported | Not reported | Methods (GEO datasets); Results (model construction and verification) | PMID 41085794 |
| Wang 2025 (47) | Multi-omics plus deep learning | TCGA-STAD, CPTAC, scRNA-seq (HRA004702, GSE183904, GSE163558, GSE308231) | Internal held-out validation set for the image-based deep-learning model; multi-omics integration for the prognostic framework | Prognosis | Internal held-out split for the image model; not an external cohort | Not reported | Not reported | Not reported | Methods (dataset collection and train/validation split); Results (model development and validation) | PMID 41360923 |

Note. Entries were verified against the full text of each article; the PubMed identifier of each source is in the final column. 'Not reported' means the item was not described in the cited article and does not establish that the analysis was not performed.
