# Supplementary material for: Public omics data reuse in gastric cancer: from discovery abundance to translational evidence
Source: Front Oncol. 2026 Oct 26;16:1974260. doi: 10.3389/fonc.2026.1974260 (PMC13746168; doi:10.3389/fonc.2026.1974260)
Supplement: Supplementary file 1 [file DataSheet1.zip › supplementary/Supplementary_Table_S6_single_cell_spatial_comparison.docx]

**Supplementary Table S6. Single-cell and spatial comparison.**

| **Method / Platform** | **Modality** | **Resolution** | **Gene / Feature Coverage** | **Key Gastric Cancer Application** | **Limitations in Secondary Analysis** |
| --- | --- | --- | --- | --- | --- |
| 10x Genomics scRNA-seq | Transcriptomics | Single cell | ~3,000-5,000 genes/cell | Tumor cell heterogeneity, TME cell atlas, lineage states (17-22) | Dissociation bias, cell-type annotation uncertainty, batch effects across datasets |
| Smart-seq2 | Transcriptomics | Single cell | Full-length (higher sensitivity) | Alternative splicing, allele-specific expression | Lower throughput, limited public data availability for gastric cancer |
| Visium (10x) | Spatial transcriptomics | ~55 μm spots (multicell) | ~18,000 genes (whole transcriptome) | Tissue architecture, tumor-stroma interface, metabolic niches (23) | Multicell resolution per spot, limited tissue regions, complex normalization |
| Xenium (10x) | Spatial in situ | Subcellular | ~300-500 targeted genes | Cell-type mapping in tissue context, ligand-receptor localization | Limited gene panel, narrower scope for discovery analyses |
| CosMx (NanoString) | Spatial in situ | Subcellular | ~1,000 genes | High-plex cell typing in gastric cancer tissue | Limited gene panel, platform-specific preprocessing |
| MERFISH (Vizgen) | Spatial transcriptomics | Subcellular | ~500-10,000 genes | Spatial organization of tumor and immune cells | Complex probe design, limited public gastric cancer datasets |
| Stereo-seq | Spatial transcriptomics | ~500 nm (subcellular) | Genome-wide | Whole-transcriptome spatial mapping at near-single-cell resolution | Very large data size, early-stage technology, limited replication studies |
| Multiplex IHC/IF | Protein (tissue) | Subcellular | ~5-60 protein markers | Immune cell quantification, spatial neighborhoods, TME validation (25,26) | Lower multiplexing than transcriptomic methods, antibody dependency |
| CellChat / NicheNet | Computational (L-R inference) | Cell cluster level | Ligand-receptor databases | Cell-cell communication hypothesis generation (59,60) | Co-expression-based inference, not direct signaling measurement; requires orthogonal validation |
| Geneformer / scGPT | Foundation model | Single cell (embedding) | Pretrained on millions of cells | Cell state annotation, perturbation prediction, integration (27-31) | Not gastric cancer-specific; benchmark against transparent baselines needed; transferability requires evaluation |
| scVI / scANVI | Probabilistic model | Single cell | Latent variable model | Integration, batch correction, differential expression | Assumptions about data distribution; requires careful parameter tuning |
