# Supplementary material for: Public omics data reuse in gastric cancer: from discovery abundance to translational evidence
Source: Front Oncol. 2026 Oct 26;16:1974260. doi: 10.3389/fonc.2026.1974260 (PMC13746168; doi:10.3389/fonc.2026.1974260)
Supplement: Supplementary file 1 [file DataSheet1.zip › supplementary/Supplementary_Table_S7_marker_level_details.docx]

**Supplementary Table S7. Marker-level detail: treatment setting, assay, cutoff, jurisdiction and evidence date for gastric cancer treatment-selection markers**

| **Marker** | **Treatment setting** | **Assay** | **Cutoff** | **Jurisdiction (approval)** | **Evidence date** |
| --- | --- | --- | --- | --- | --- |
| HER2 amplification | Advanced or metastatic gastric / gastro-oesophageal junction adenocarcinoma | Immunohistochemistry with in-situ hybridisation confirmation | IHC 3+, or IHC 2+ with ISH positivity | FDA; EMA; NMPA | Reviewed to 2026 |
| MSI-H / dMMR | Advanced solid tumours including gastric cancer; second-line or later for some regimens | PCR; next-generation sequencing; immunohistochemistry | Categorical (MSI-H or dMMR); no numerical cutoff | FDA; EMA; NMPA | Reviewed to 2026 |
| PD-L1 combined positive score (CPS) | Advanced or metastatic gastric / gastro-oesophageal junction adenocarcinoma; defined first-line settings | Immunohistochemistry (22C3 or 28-8 assay) | CPS ≥ 1 or CPS ≥ 5, depending on regimen and jurisdiction | FDA; EMA; NMPA | Reviewed to 2026 |
| Claudin 18.2 (CLDN18.2) | First-line HER2-negative advanced gastric / gastro-oesophageal junction adenocarcinoma | Immunohistochemistry | Moderate-to-strong membranous staining in ≥ 75% of tumour cells | Japan; China (review ongoing elsewhere) | Reviewed to 2026 |
| FGFR2b | Investigational in gastric cancer; no approved treatment-selection indication | Immunohistochemistry and/or circulating tumour DNA (research use) | Not standardised | None (investigational) | Reviewed to 2026 |
| Epstein-Barr virus (EBV) status | Not a treatment-selection setting; used as a molecular-subtype and prognostic classifier | In-situ hybridisation for EBV-encoded RNA | Categorical (positive or negative) | Not applicable | Reviewed to 2026 |
| Tumour mutational burden (TMB) | Investigational for treatment selection in gastric cancer | Next-generation sequencing (panel-dependent) | Panel-dependent; not harmonised across platforms | Not approved for gastric cancer treatment selection | Reviewed to 2026 |

Note. Values reflect the evidence reviewed for this article. Inclusion of a marker does not imply that a public-omics association is sufficient for clinical use.
